# Supplementary material for: Desmopressin treatment combined with clotting factor VIII concentrates in patients with non-severe haemophilia A: protocol for a multicentre single-armed trial, the DAVID study
Source: BMJ Open. 2019 Apr 23;9(4):e022719. doi: 10.1136/bmjopen-2018-022719 (PMC6500101; doi:10.1136/bmjopen-2018-022719)
Supplement: Supplementary file 1 [file bmjopen-2018-022719supp001.pdf]

Supplementary data

1) Steering committee
2) Participating centres
3) Handling and storage of data and documents
4) Amendments
5) Compensation for injury
6) Access to final trial dataset
7) Model for patient information and informed consent form (Dutch only)

1) **Steering committee**

Function	Contact data
Project Leader	Dr. M.J.H.A. Kruip, haematologist<br>T: 010-7033123, E: m.kruip@erasmusmc.nl<br>Department of Haematology, Erasmus University Medical Centre<br>Postbus 2040, 3000 CA Rotterdam
Sponsor	Prof. Dr. F.W.G. Leebeek, haematologist<br>T:010-7033123, E:f.leebeek@erasmusmc.nl<br>Department of Haematology, Erasmus University Medical Centre
Co-project leader	Dr. M.H. Cnossen, paediatric haematologist<br>T:010-7036691, E: m.cnossen@erasmusmc.nl<br>Department of Paediatric Haematology, Erasmus University Medical Centre
Co-project leader	Prof.dr. R.A.A. Mathôt, clinical pharmacologist<br>T:020-5663474, E:r.mathot@amc.uva.nl<br>Pharmacy, Amsterdam UMC
Project Advisor	Dr. M.H.E. Driessens<br>T:06-12192825020-6599021, E: m.driessens@nvhp.nl<br>Dutch Society of Haemophilia Patients
Promotor	Prof. dr. F.W.G. Leebeek, haematologist<br>T:010-7033123, E:f.leebeek@erasmus.nl<br>Department of Haematology, Erasmus University Medical Centre
Member of the steering committee	Dr. C.J. Fijn Van Draat<br>T: 020-5669111, E: c.j.fijnvandraat@amc.uva.nl<br>Department of Paediatric Haematology, Amsterdam UMC
Member of the steering committee	Dr. S. Polinder<br>T: 010-7043954, E:s.polinder@erasmusmc.nl<br>Department of Public Health, Erasmus University Medical Centre

2) List of participating centres

Medical Centre	Member and contact data
**Erasmus University Medical Centre**	Dr. M.J.H.A. Kruip, hematologist<br>T: 010-7033123, E: m.kruip@erasmusmc.nl<br>Department of Haematology<br>Postbus 2040, 3000 CA Rotterdam
**Amsterdam University Medical Centres**	Dr. M. Coppens<br>Department of haematology<br>m.coppens@amc.uva.nl
**Leiden University Medical Centre**	Prof. Dr. H.C.J. Eikenboom<br>Department of internal medicine, division of Thrombosis and Haemostasis<br>h.c.j.eikenboom@lumc.nl
**University Medical Centre Groningen**	Prof. dr. K. Meijer<br>Department of Haematology<br>k.meijer@umcg.nl
**Radboud University Medical Centre, Nijmegen**	Dr. B.A.P. Laros-van Gorkom<br>Department of Haematology<br>britta.laros-vangorkom@radboudumc.nl
**Maxima Medical Centre, Eindhoven**	Dr. L. Nieuwenhuizen<br>Department of Haematology<br>l.nieuwenhuizen@mmc.nl
**University Medical Centre Utrecht**	Dr. E.P. Mauser-Bunschoten<br>Van Creveldkliniek, Department of Haematology<br>e.mauserbunschoten@umcutrecht.nl
**Maastricht University Medical Centre**	Dr. E.A.M. Beckers<br>Department of Haematology<br>eam.beckers@mumc.nl

3) Handling and storage of data and documents

All data will be handled confidentially. To assure anonymity of all participating subjects, data will be coded. Only the different investigators at the participating sites and the treating physician will be able to match the study number to the patient’s file by matching this number to the patient’s hospital registration number. Handling of data will be done according to the Dutch Personal Data Protection Act. DNA and blood samples will be handled confidentially and stored in the hemostasis laboratory at the Erasmus MC. The material will be stored for a maximum of fifteen years.

4) Amendments

Amendments are changes made to the research after a favourable opinion by the accredited METC has been given. All substantial amendments will be notified to the METC and to the competent authority. Non-substantial amendments will not be notified to the accredited METC and the competent authority, but will be recorded and filed by the sponsor. All amendments will be communicated to all participating centres by the trial coordinator. Changes regarding inclusion and exclusion criteria or study design, will also be communicated to www.trialregister.nl (NTR 5383).

5) Compensation for injury

The sponsor/investigator has a liability insurance which is in accordance with article 7, subsection 9 of the WMO. The sponsor/investigator has a liability insurance which is in accordance with article 7 of the WMO. The sponsor (also) has an insurance which is in accordance with the legal requirements in the Netherlands (Article 7 WMO). This insurance provides cover for damage to research subjects through injury or death caused by the study. The insurance applies to the damage that becomes apparent during the study or within 4 years after the end of the study.

6) Access to final trial dataset

The sponsor of the DAVID study will have access to the final dataset and will be the owner of all study data. However, all participating centres have access to their own data and will have permission to use these data for non-commercial research within their own centre and to improve patient care. All publications regarding the DAVID study will be coordinated by the project leader. Other centres than the sponsor site may only publish data after permission from the sponsor and if the sponsor is not publishing the data within a fair amount of time after the study and data analysis has ended. All participating centres have contractual agreements with the sponsor regarding these matters.

In addition the sponsor has an agreement with the funder. The funder will be co-owner of the final dataset. All data has to be available for future research. The sponsor is obliged to publish all study data unless publication does not serve any public interest.

7) Model for patient information and informed consent form (Dutch only)

Informatiebrief DAVID-studie
Geachte patiënt,

Graag willen wij u met deze brief informeren over de DAVID-studie. DAVID staat voor *D**DAV**P treatment comb**I**ne**D** with factor VIII clotting factor concentrates in patients with mild hemophilia A*. Dit betekent: combinatiebehandeling van DDAVP en factor VIII-concentraten in patiënten met milde en matige hemofilie A.

U krijgt deze brief, omdat we willen vragen of u deel wilt nemen aan de DAVID-studie.
Lees deze brief rustig door en neem de tijd om na te denken over het al dan niet deelnemen aan de DAVID-studie. Bespreek het eventueel ook met uw partner, vrienden of familie. Lees ook de algemene brochure. Daarin staat veel algemene informatie over medisch-wetenschappelijk onderzoek. Heeft u na het lezen nog vragen? Dan kunt u altijd terecht bij uw behandelend arts of de onderzoeker. Ook is er een onafhankelijke arts, die veel weet van het onderzoek. Aan het einde van deze brief vindt u de contactgegevens.

Inleiding
U bent onder behandeling in verband met milde of matige hemofilie A, een zeldzame, erfelijke stoornis in de bloedstolling. Hierbij heeft u te weinig stollingsfactor VIII (factor VIII). Daardoor loopt u meer risico op bloedingen, zoals bij een kleine verwonding of tijdens een operatie. De behandeling hiervoor bestaat meestal uit het geven van factor VIII-concentraat, de ontbrekende stollingsfactor. Factor VIII-concentraat gedraagt zich in ieder lichaam anders. Dit betekent dat de ene patiënt meer factor VIII-concentraat nodig heeft dan de andere patiënt. Daar wordt nu niet genoeg rekening mee gehouden. Hierdoor krijgen sommige patiënten mogelijk te veel factor VIII-concentraat toegediend, terwijl andere patiënten juist te weinig krijgen.
Patiënten met milde en matige hemofilie A hebben nog een kleine hoeveelheid eigen factor VIII in het bloed en worden in sommige gevallen met DDAVP behandeld. Dit medicijn zorgt ervoor dat het eigen, opgeslagen factor VIII vrijkomt in het bloed. DDAVP is vaak niet voldoende om het factor VIII-gehalte in het bloed hoog genoeg te krijgen bij operaties. Een oplossing is om DDAVP te combineren met factor VIII-concentraat. Het factor VIII-gehalte stijgt dan in eerste instantie door de DDAVP. Vervolgens wordt er zo veel factor VIII-concentraat bijgegeven dat er voldoende factor VIII in het bloed zit. In deze studie willen wij bestuderen of de combinatiebehandeling goed werkt.

1. Wat is het doel van het onderzoek?
We willen onderzoeken of patiënten rondom operaties en bloedingen de juiste factor VIII-waarden in het bloed bereiken wanneer ze behandeld worden met een combinatiebehandeling van DDAVP en factor VIII-concentraat. Als dit het geval is, dan kan de combinatiebehandeling in de toekomst standaard gebruikt gaan worden. Dit zal er hopelijk toe leiden dat er minder factor VIII-concentraat nodig is. Wij willen dit bereiken zonder dat de behandeling slechter of minder veilig wordt.
Daarnaast willen we nagaan of de benodigde hoeveelheid factor VIII-concentraat per patiënt kan worden bepaald door van alle deelnemers aan de DAVID-studie verschillende gegevens te verzamelen.

2. Welke behandeling wordt onderzocht?
Tijdens dit onderzoek wordt de combinatiebehandeling van DDAVP en factor VIII-concentraat onderzocht. Beide medicijnen worden al gebruikt bij milde en matige hemofilie A-patiënten en u heeft ze mogelijk al eens gehad.

3. Hoe wordt het onderzoek uitgevoerd?

Iedereen die deelneemt aan dit onderzoek krijgt minimaal 2 dagen een combinatiebehandeling van DDAVP en factor VIII-concentraat rondom een operatie of een bloeding.
De combinatiebehandeling begint bij voorkeur vlak voor de ingreep. In het geval van een spoedingreep of bloeding kan het zijn dat u eerst maximaal 24 uur behandeld wordt met FVIII-concentraat voordat de combinatiebehandeling begint. De combinatiebehandeling duurt in eerste instantie maximaal drie aaneengesloten dagen. Dagelijks krijgt u eenmaal DDAVP toegediend via een infuus. Aansluitend krijgt u factor VIII-concentraat toegediend via een infuus, om de juiste factor VIII-waarden te bereiken. Afhankelijk van hoe lang u behandeling nodig heeft, beslist uw eigen arts of u van dag 6 t/m 8 opnieuw de combinatiebehandeling krijgt. Op alle overige behandeldagen ontvangt u alleen factor VIII-concentraat.

<u>DDAVP</u>

DDAVP krijgt u in de standaarddosering via een infuus. Om er zeker van te zijn dat dit middel bij u werkt, hebben we de resultaten van een DDAVP-test nodig. Dit kunnen resultaten zijn van een test die eerder is uitgevoerd. Indien er voor een geplande operatie geen testresultaten beschikbaar zijn of zijn ze niet geschikt, dan zal uw arts met u een nieuwe test afspreken voorafgaand aan de operatie. Tijdens de test zal u 1 dosis DDAVP toegediend krijgen via een infuus. Zowel voor als 2-3 keer na de toediening van DDAVP zal er bloed worden afgenomen om uw reactie op DDAVP te meten. Meer uitleg over de gang van zaken tijdens de test ontvangt u van uw eigen arts.
Hierboven heeft u kunnen lezen dat u maximaal 3 aaneengesloten dagen DDAVP krijgt. Dit doen we, omdat de voorraad van factor VIII na meerdere DDAVP-toedieningen minder wordt. Daarom is het belangrijk dat uw lichaam na 3 aaneengesloten dagen, 3 dagen ‘rust’ krijgt, waarin u geen DDAVP krijgt. Na 3 ‘rust’-dagen kunt u weer DDAVP ontvangen.

<u>Factor VIII-concentraten</u>

Gedurende het onderzoek krijgt u hetzelfde factor VIII-concentraat (bijvoorbeeld Novoeight of Kogenate) dat u anders ook zou krijgen. Op basis van de resultaten van de DDAVP-test en de gemeten factor VIII-waarden wordt er rondom de operatie of bloeding dagelijks een persoonlijke dosering bepaald en doorgegeven aan uw eigen arts. Uw eigen arts beslist hoe lang u factor VIII-concentraat toegediend krijgt en of het factor VIII-concentraat continu of in losse giften per dag gegeven wordt.

<u>Vragenlijst</u>

U krijgt voor de start van de combinatiebehandeling en 3 dagen erna een korte vragenlijst met 14 vragen over hoe u de zorg rondom de operatie of bloeding hebt ervaren. Deze vragenlijst richt zich op de behandeling van uw hemofilie A.

4. Wat wordt er van u verwacht?

Tijdens het onderzoek vragen wij u de voorschriften van uw arts te volgen. Dit houdt onder andere in dat u gedurende de behandeling met DDAVP niet meer vocht binnenkrijgt dan 1,5 liter per dag. Verder is er een extra ziekenhuisbezoek nodig, indien u een DDAVP-test nodig hebt. Dit hoort u van uw eigen arts.

5. Wat is er meer of anders dan de reguliere behandeling?

De reguliere behandeling rondom operaties en bloedingen bij patiënten met hemofilie A bestaat uit het geven van factor VIII-concentraat. Tijdens het onderzoek krijgt u daarnaast DDAVP. U ontvangt de combinatiebehandeling maximaal twee perioden van 3 aaneengesloten dagen. Tussen deze twee

perioden, zitten 3 dagen zonder DDAVP. Indien nodig krijgt u voor een geplande operatie een DDAVP-test om te zien hoe u op deze medicatie reageert.
Daarnaast wordt onderzocht of u voldoende reageert op de combinatiebehandeling en of u de juiste dosering krijgt. Hiervoor nemen we tijdens de combinatiebehandeling, afhankelijk van de dag, 1-2 keer per dag vaker bloed af, 15 milliliter per keer. Zie hiervoor bijlage 2.
Verder onderzoeken we of veranderingen of verschillen in het DNA mogelijk samenhangen met de manier waarop u op de behandeling reageert. Hiervoor wordt eenmalig bloed afgenomen. Dit gebeurt alleen als u hiervoor toestemming geeft.
In bijlage 2 vindt u een schematisch overzicht van de onderzoeken. Hierop staat aangegeven welke behandelingen en onderzoeken extra zijn ten opzichte van de reguliere behandeling.

6. Wat zijn de mogelijke andere behandelingen?
Wanneer u besluit niet deel te nemen aan het onderzoek, wordt u behandeld met factor VIII-concentraat in de standaarddosering.

7. Welke bijwerkingen kunt u verwachten?
Bijwerkingen van DDAVP zijn van voorbijgaande aard en over het algemeen mild. De meest voorkomende bijwerkingen zijn een tijdelijke daling van de bloeddruk, een stijging van de hartslag, blozen, hoofdpijn en jeukende ogen. In zeldzame gevallen houdt het lichaam te veel vocht vast, waardoor het zoutgehalte in het bloed te laag kan worden.
De belangrijkste bijwerking van factor VIII-concentraten is een verhoogd risico op het ontwikkelen van remmers tegen factor VIII. Remmers zijn stoffen waardoor mogelijk de werkzaamheid van factor VIII-concentraten bij u in het vervolg verminderd kan zijn.

Niet alle bijwerkingen die bekend zijn, worden hier vermeld. Het is ook niet zo dat alle genoemde bijwerkingen bij elke patiënt zullen optreden. Wanneer u klachten krijgt, vragen wij u dit altijd aan uw eigen arts te melden.

8. Wat zijn de mogelijke voor- en nadelen van deelname?
Aan deelname zijn voor u geen directe voordelen verbonden. Met uw deelname kunt u wel de behandeling van milde en matige hemofilie A-patiënten helpen verbeteren.
Waarschijnlijk heeft u minder factor VIII-concentraat nodig dan u buiten de studie nodig zou hebben, omdat u ook DDAVP zal krijgen. Mogelijk kan dit het risico op het ontwikkelen van remmers tegen factor VIII verminderen. De ontwikkeling van remmers zorgt ervoor dat de behandeling met FVIII-concentraat minder effectief zal worden.
Het nadeel van deelname is dat er ten opzichte van de reguliere behandeling dagelijks 1 tot 2 extra bloedafnames plaatsvinden gedurende de ziekenhuisopname. Daarnaast moet u een DDAVP-test ondergaan als er geen geschikte eerdere resultaten beschikbaar zijn. Deze resultaten kunnen ook buiten de studie van nut zijn voor uw behandeling. Tot slot kunt u mogelijk bijwerkingen krijgen van de DDAVP.

9. Wat gebeurt er als u niet wenst deel te nemen aan het onderzoek?
Het is uw beslissing om wel of niet aan deze studie deel te nemen. U kunt besluiten om niet deel te nemen of uw toestemming tot deelname aan de studie op elk moment intrekken, zonder dat dit de zorg die u van artsen of verpleging in het ziekenhuis krijgt, beïnvloedt.

10. Wat gebeurt er als het onderzoek is afgelopen?
Het kan voorkomen dat u of uw behandelend arts besluit uw deelname aan het onderzoek te stoppen. U kunt dat zonder opgave van redenen en op ieder moment beslissen. U zal dan overgaan op de reguliere

behandeling die bestaat uit het toedienen van uitsluitend factor VIII-concentraat. Uw behandelend arts zal uw deelname aan het onderzoek alleen stoppen als dat noodzakelijk wordt geacht, bijvoorbeeld vanwege uw gezondheid.

11. Bent u verzekerd wanneer u aan het onderzoek meedoet?

Voor iedereen die aan dit onderzoek deelneemt, is een verzekering afgesloten. De verzekering dekt de schade als gevolg van het onderzoek. Dit geldt voor schade die tijdens het onderzoek optreedt. In bijlage 3 vindt u de verzekeringsgegevens.

12. Wordt u geïnformeerd als er tussentijds voor u relevante informatie over de studie bekend wordt?

Het onderzoek zal zo nauwkeurig mogelijk volgens plan verlopen. De situatie kan echter veranderen, bijvoorbeeld doordat er nieuwe informatie wordt verkregen. In dat geval wordt dat met u besproken. U beslist dan zelf of u met het onderzoek wilt stoppen of doorgaan. Als uw veiligheid of welbevinden in gevaar is, stoppen we direct met het onderzoek.

13. Wat gebeurt er met uw gegevens en lichaamsmateriaal?

In de algemene brochure is uitgelegd dat de onderzoeker gegevens over u verzamelt en deze vertrouwelijk behandelt. Dit betekent dat een aantal personen uw medische status en de gegevens van het onderzoek mogen inzien. Deze personen mogen de gegevens gebruiken voor dit onderzoek, maar zij mogen deze gegevens alleen bekend maken zonder daarbij uw naam of andere persoonlijke gegevens te vermelden. Uw identiteit blijft dus altijd geheim. De onderzoeker bewaart de gegevens met een code. Dit betekent dat op de studie- documenten in plaats van uw naam alleen een letter-cijfercode staat. Alleen de onderzoeker houdt een lijst bij waarop staat welke letter- cijfercode bij welke naam hoort.

Normaal gesproken heeft alleen uw behandelend arts en zijn/ haar team inzage in uw gegevens. Als u meedoet aan deze studie krijgen meer mensen inzage in uw medische gegevens en studiegegevens. De personen die inzage kunnen krijgen in uw gegevens zijn:

- de medewerkers van het onderzoeksteam,
- de leden van de toetsingscommissie die de studie heeft goedgekeurd,
- de bevoegde medewerkers van de Inspectie voor de Gezondheidszorg

Na de studie worden de gecodeerde gegevens gedurende 15 jaar bewaard. Dit is nodig om alles goed te kunnen controleren. Bovendien willen wij graag uw gegevens gebruiken voor andere onderzoeken die worden uitgevoerd naar milde en matige hemofilie A.
Deze onderzoeken hebben dus eenzelfde doel als het onderzoek waarvoor u nu wordt gevraagd. Het is dus niet zo dat uw gegevens ook zullen worden gebruikt voor onderzoek naar een geheel andere aandoening of een heel ander probleem. Vanzelfsprekend blijft de vertrouwelijkheid die we hierboven hebben beschreven altijd gelden. Vindt u het goed als wij uw gegevens gebruiken? Als u dat niet wilt, respecteren wij dat natuurlijk. U kunt uw keuze op het toestemmingsformulier aangeven.
Na de studie wordt uw lichaamsmateriaal (bloedmonsters en eventueel DNA) in gecodeerde vorm gedurende 15 jaar bewaard. Na een tijdelijke opslag in uw eigen ziekenhuis, zal het materiaal worden opgeslagen in het Erasmus Universitair Medisch Centrum Rotterdam. Wij willen dit materiaal graag gebruiken voor andere onderzoeken die worden uitgevoerd naar milde en matige hemofilie A. Deze onderzoeken hebben dus eenzelfde doel als het onderzoek waarvoor u nu wordt gevraagd. Het is dus niet zo dat uw materiaal ook zal worden gebruikt voor onderzoek naar een geheel andere aandoening of een heel ander probleem. Vanzelfsprekend blijft de vertrouwelijkheid die we hierboven hebben

beschreven altijd gelden. Vindt u het goed als wij uw materiaal bewaren en gebruiken? Als u dat niet wilt, respecteren wij dat natuurlijk. U kunt uw keuze op het toestemmingsformulier aangeven.

14. Wordt uw huisarts en/of behandelend specialist geïnformeerd bij deelname?
Wij laten uw huisarts schriftelijk weten dat u meedoet aan het onderzoek. Dit is voor uw eigen veiligheid. U moet hiervoor toestemming geven op het toestemmingsformulier. Als u geen toestemming geeft, kan u niet meedoen aan het onderzoek.

15. Krijgt u een vergoeding voor deelname?
Indien u voorafgaand aan de deelname aan de studie nog geen DDAVP-test gehad en u hiervoor een extra keer naar het hemofiliebehandelcentrum moet komen, dan krijgt u een vergoeding voor de reis- en eventuele parkeerkosten. Verder is er aan deelname aan de studie geen vergoeding verbonden.

16. Welke medisch-ethische toestingscommissie heeft dit onderzoek goedgekeurd?
Toetsingscommissie METC Erasmus MC heeft dit onderzoek goedgekeurd. Meer informatie over de goedkeuring vindt u in de algemene brochure.

17. Waar kunt u met vragen over het onderzoek terecht?
Met vragen over het onderzoek kunt u terecht bij uw hemofiliebehandelcentrum. In bijlage 1 vindt u de contactgegevens. Tevens vindt u daar de gegevens van een onafhankelijk arts aan wie u ook vragen kunt stellen over deelname aan het onderzoek.
Als u niet tevreden bent over het onderzoek of de behandeling, kunt u een klacht indienen bij de klachtencommissie. In bijlage 1 vindt u de benodigde contactgegevens.

Indien u na zorgvuldige overweging besluit deel te nemen aan dit wetenschappelijk onderzoek, dan vragen we u om samen met de onderzoeker het toestemmingsformulier te ondertekenen en te dateren.

Met vriendelijke groet,

Namens het onderzoeksteam,

Dr. Marieke J.H.A. Kruip en drs. Lisette M. Schütte

Bijlagen

1. Contactgegevens
2. Schema met onderzoeken
3. Informatie over de verzekering
4. Toestemmingsverklaring
5. Algemene brochure medisch-wetenschappelijk onderzoek met mensen

Bijlage 1 – contactgegevens

U kunt meer informatie over het onderzoek krijgen bij de onderzoeker of bij één van uw behandelaren:

Hematologen:
<Namen >

Verpleegkundig specialist hemofilie:
<Namen >

Hemofilieverpleegkundigen:
<Namen >

Contactgegevens:
Hemofiliebehandelcentrum <naam zh>:
<adres HBC>
e-mail: <emailadres HBC >
david@erasmusmc.nl

Secretariaat hematologie: <tel>

Onderzoekers:
Dr. Marieke J.H.A. Kruip
Drs. L.M. Schütte

Onafhankelijk deskundige

Als u twijfelt over deelname aan dit onderzoek, kunt u ook een onafhankelijk arts raadplegen, die zelf niet bij het onderzoek is betrokken, maar wel deskundig is op dit gebied:
Dr. P.A.W. te Boekhorst, internist-hematoloog, tel: 010-7033123
Ook als u vragen voor of tijdens het onderzoek hebt, die u liever niet aan de onderzoekers stelt, kunt u contact opnemen met de onafhankelijke arts.

Klachten

Een klacht kunt u indienen bij
<Onafhankelijke klachtencommissie of andere procedure>

Bijlage 2 – Schema met onderzoeken; zodra de behandeling is afgerond, zijn bloedafnames niet meer nodig

In het geval van een geplande ingreep

	Screening	Opnameperiode					Vervolg periode
Onderzoek	Voor de operatie	Dag van de operatie	Dag 1 na de operatie	Dag 2 na de operatie indien nodig	Dag 3-5 na de operatie indien nodig	Dag 6-8 na de operatie indien nodig	8-10 weken na operatie
DNA-afname*	V						
DDAVP-test*	V						
Bloedonderzo	V	X-V-X-X	X-V-V	X-V-V	X	X-V-V	

ek**/***							
DDAVP-toediening**		V	V	V		V	
factor VIII-concentraat volgens doseeradvies**		X	X	X	X	X	
Vragenlijst	V				V		
Bloedonderzoek remmers							X

*alleen indien nodig; zie eerdere informatie in de brief
**alleen indien behandeling nog noodzakelijk is; dit hoort u van uw eigen arts
***hier zijn alle bloedafnamen genoemd, ook de standaard afnames buiten de studie.
X: Deze onderzoeken en behandelingen zijn standaard.
V: Deze onderzoeken en behandelingen zijn extra voor de studie.

In het geval van een spoedingreep of een bloeding

	Opnameperiode					Vervolg periode
Onderzoek	Dag van start DDAVP	Dag 1 na start DDAVP	Dag 2 na start DDAVP indien nodig	Dag 3-5 na start DDAVP indien nodig	Dag 6-8 na start DDAVP indien nodig	8-10 weken na operatie /bloeding
DNA-afname*	V					
Bloedonderzoek**/***	X-V-X-X	X-V-V	X-V-V	X	X-V-V	
DDAVP-toediening**	X	X	X		X	
factor VIII-concentraten volgens doseeradvies**	X	X	X	X	X	
Vragenlijst	V			V		
Bloedonderzoek remmers	V					X

*alleen indien nodig; zie eerdere informatie in de brief
** alleen indien behandeling nog noodzakelijk is; dit hoort diegene die u vertegenwoordigt, van zijn eigen arts
*** hier zijn alle bloedonderzoeken genoemd, ook de bloedonderzoeken die behoren tot de standaardzorg.
X: Deze onderzoeken en behandelingen zijn standaard.
V: Deze onderzoeken en behandelingen zijn extra voor de studie

Bijlage 3 – Informatie over de verzekering
Voor iedereen die meedoet aan dit onderzoek, heeft het Erasmus MC een verzekering afgesloten. De verzekering dekt schade door deelname aan het onderzoek. Dit geldt voor schade tijdens het onderzoek of binnen vier jaar na het einde ervan. Schade moet u binnen die vier jaar aan de verzekeraar hebben gemeld.
De verzekering dekt niet alle schade. Onderaan deze tekst staat in het kort welke schade niet wordt gedekt.
Deze bepalingen staan in het Besluit verplichte verzekering bij medisch-wetenschappelijk onderzoek met mensen. Dit besluit staat op www.ccmo.nl, de website van de Centrale Commissie Mensgebonden Onderzoek (zie 'Bibliotheek' en dan 'Wet- en regelgeving').

Bij schade kunt u direct contact leggen met:

Van Ameyde
Ter attentie van mw. K. Roberts
Postbus 3038
2280 GA Rijswijk
T +31 (0) 70 413 7300
marketform@vanameyde.com

De verzekering biedt een dekking van € 650.000 per proefpersoon en € 5.000.000 voor het hele onderzoek (en € 7.500.000 per jaar voor alle onderzoeken van dezelfde opdrachtgever).

De verzekering dekt de volgende schade niet:
- schade door een risico waarover u in de schriftelijke informatie bent ingelicht. Dit geldt niet als het risico zich ernstiger voordoet dan was voorzien of als het risico heel onwaarschijnlijk was;
- schade aan uw gezondheid die ook zou zijn ontstaan als u niet aan het onderzoek had meegedaan;
- schade door het niet (volledig) opvolgen van aanwijzingen of instructies;
- schade aan uw nakomelingen, als gevolg van een negatief effect van het onderzoek op u of uw nakomelingen;
- schade door een bestaande behandelmethode bij onderzoek naar bestaande behandelmethoden.

Betreft: DAVID-studie

Toestemmingsformulier voor volwassen deelnemers

Naam: ………………….. Geboortedatum: __ / __ / __

Ik bevestig dat ik de informatiebrief voor deelnemers heb gelezen. Ik begrijp de informatie. Ik heb de gelegenheid gehad om aanvullende vragen te stellen. Deze vragen zijn naar tevredenheid beantwoord. Ik heb voldoende tijd gehad om over deelname na te denken.

Ik weet dat mijn deelname geheel vrijwillig is en dat ik mijn toestemming op ieder moment kan intrekken, zonder dat ik daarvoor een reden hoef te geven.

Ik geef toestemming voor deelname aan bovengenoemd onderzoek.

Ik geef toestemming om mijn huisarts te vertellen dat ik meedoe aan dit onderzoek.

Ik geef toestemming aan bevoegde personen van het onderzoeksteam, medewerkers van de Inspectie voor de Gezondheidszorg en leden van de medisch-ethische toetsingscommissie voor inzage in mijn medische gegevens en onderzoeksgegevens.

Ik geef toestemming om de gegevens te verwerken voor de doeleinden zoals beschreven in de informatiebrief.

Ik geef toestemming voor het bewaren van de onderzoeksgegevens na afloop van het onderzoek voor een periode van 15 jaar.

Ik geef toestemming om mijn huisarts in te lichten over deelname aan dit onderzoek.

U wordt gevraagd in onderstaande zinnen door te halen wat niet van toepassing is.

Ik geef **WEL/GEEN** toestemming om de onderzoeksgegevens te gebruiken voor toekomstig onderzoek.

Ik geef **WEL/GEEN** toestemming om lichaamsmateriaal te gebruiken voor toekomstig onderzoek en te bewaren voor een periode van maximaal 15 jaar.

Ik geef **WEL/GEEN** toestemming om DNA-materiaal te gebruiken voor toekomstig onderzoek en te bewaren voor een periode van maximaal 15 jaar.

Ik wil meedoen aan dit onderzoek.

Naam deelnemer :

Handtekening : Datum : __ / __ / __

Hierbij verklaar ik, de onderzoeker, dat ik de deelnemer in de gelegenheid heb gesteld om aanvullende vragen te stellen, welke ik alle naar waarheid heb beantwoord.
Alle gegevens die mij inzake het onderzoek ter inzage zullen komen, zal ik geheimhouden en met respect behandelen.

Naam onderzoeker (of diens vertegenwoordiger) :

Handtekening : Datum : __ / __ / __
