# Supplementary material for: Desmopressin treatment combined with clotting factor VIII concentrates in patients with non-severe haemophilia A: protocol for a multicentre single-armed trial, the DAVID study
Source: BMJ Open. 2019 Apr 23;9(4):e022719. doi: 10.1136/bmjopen-2018-022719 (PMC6500101; doi:10.1136/bmjopen-2018-022719)
Supplement: Author's manuscript [file bmjopen-2018-022719.draft_revisions.pdf]

BMJ Open is committed to open peer review. As part of this commitment we make the peer review history of every article we publish publicly available.

When an article is published we post the peer reviewers' comments and the authors' responses online. We also post the versions of the paper that were used during peer review. These are the versions that the peer review comments apply to.

The versions of the paper that follow are the versions that were submitted during the peer review process. They are not the versions of record or the final published versions. They should not be cited or distributed as the published version of this manuscript.

BMJ Open is an open access journal and the full, final, typeset and author-corrected version of record of the manuscript is available on our site with no access controls, subscription charges or pay-per-view fees (http://bmjopen.bmj.com).

If you have any questions on BMJ Open's open peer review process please email info.bmjopen@bmj.com

BMJ Open

Desmopressin treatment combined with clotting factor VIII concentrates in patients with non-severe haemophilia A: a multicentre single-armed trial, the DAVID study

Journal:	*BMJ Open*
Manuscript ID	bmjopen-2018-022719
Article Type:	Protocol
Date Submitted by the Author:	07-Mar-2018

Complete List of Authors:	Schütte, Lisette; Erasmus MC, Haematology<br>Cnossen, Marjon; Erasmus MC Sophia, Pediatric Hematology<br>Van Hest, Reinier; AMC, Hospital Pharmacy<br>Driessens, Mariette; Netherlands Haemophilia Patient Society<br>Fijnvandraat, Karin; Emma Kinderziekenhuis AMC, Pediatric Hematology; Sanquin Research, Plasma Proteins<br>Polinder, Suzanne; Erasmus MC, Public Health<br>Beckers, Erik; Maastricht University Medical Centre, Haematology<br>Coppens, Michiel; Academic Medical Center, Vascular Medicine<br>Eikenboom, Jeroen; Leids Universitair Medisch Centrum, Thrombosis and Haemostasis<br>Laros-van Gorkom, Britta; Radboudumc, Haematology<br>Meijer, Karina; Universitair Medisch Centrum Groningen, Department of Haematology<br>Nieuwenhuizen, Laurens; Maxima Medisch Centrum, Haematology<br>Mauser-Bunschoten, Evelien; University Medical Centre Utrecht, Van Creveldclinic<br>Leebeek, Frank; Erasmus MC, Hematology<br>Mathôt, Ron; Academic Medical Center, Hospital Pharmacy<br>Kruip, Marieke; Erasmus MC, Hematology

Keywords:	Haemophilia A, Desmopressin, FVIII concentrate, SURGERY, Pharmacokinetic modelling

SCHOLARONE™
Manuscripts

Desmopressin treatment combined with clotting factor VIII concentrates in patients with non-severe haemophilia A: a multicentre single-armed trial, the DAVID study

L.M. Schütte[1], M.H. Cnossen[2], R.M. van Hest[3], M.H.E. Driessens[4], K. Fijnvandraat[5], S. Polinder[6], E.A.M. Beckers[7], M. Coppens[8], J. Eikenboom[9], B.A.P. Laros-van Gorkom[10], K. Meijer[11], L. Nieuwenhuizen[12], E.P. Mauser-Bunschoten[13], F.W.G. Leebeek[1], R.A.A. Mathôt[3], M.J.H.A. Kruip[1]

Corresponding Author:
Dr. Marieke J.H.A. Kruip
Room Na-823
P.O. Box 2040
3000 CA Rotterdam
The Netherlands
e-mail: m.kruip@erasmusmc.nl
telephone number: +31 10 7033123

Affiliations
[1]Department of Haematology, Erasmus University Medical Centre, Rotterdam, the Netherlands; [2]Department of Paediatric Haematology, Erasmus University Medical Centre - Sophia Children's Hospital, Rotterdam, the Netherlands; [3]Department of Hospital Pharmacy, Academic Medical Centre, Amsterdam, the Netherlands; [4]Netherlands Haemophilia Patient Society, Nijkerk, the Netherlands; [5]Department of Paediatric Haematology, Academic Medical Centre, Amsterdam, The Netherlands; Department of Plasma Proteins, Sanquin Research, Amsterdam, The Netherlands; [6]Department of Public Health, Erasmus University Medical Centre, Rotterdam, the Netherlands; [7]Department of Haematology, Maastricht University Medical Centre, Maastricht, the Netherlands; [8]Department of Vascular Medicine, Academic Medical Centre, Amsterdam, the Netherlands; [9]Department of Thrombosis and Haemostasis, Leiden University Medical Centre, Leiden, the Netherlands; [10]Department of Haematology, Radboud University Medical Centre, Nijmegen, the Netherlands; [11]Department of Haematology, University Medical Centre Groningen, Groningen, the Netherlands; [12]Department of Haemotology, Maxima Medical Centre, Eindhoven, the Netherlands; [13]Van Creveldkliniek, Utrecht, the Netherlands

Word count: 3391

For peer review only

Abstract

Introduction:

Haemophilia A is an inherited bleeding disorder characterised by factor VIII (FVIII) deficiency. In non-severe haemophilia A patients, surgery and bleeding are the main indications for treatment with FVIII concentrate. A recent study reported that standard dosing frequently results in FVIII levels (FVIII:C) below or above FVIII target ranges, leading to respectively a bleeding risk or excessive costs. In addition, FVIII concentrate treatment carries a risk of development of neutralizing antibodies.

An alternative is desmopressin, which releases endogenous FVIII and von Willebrand factor. In most non-severe haemophilia A patients, desmopressin alone is not enough to achieve FVIII target levels during surgery or bleeding. We hypothesize that combined pharmacokinetic (PK) guided administration of desmopressin and FVIII concentrate may improve dosing accuracy and reduces FVIII concentrate consumption.

Methods and analysis:

In the DAVID study, fifty non-severe haemophilia A patients (FVIII:C ≥0.01 IU/ml) with a bleeding episode or undergoing surgery will receive desmopressin and FVIII concentrate combination treatment. The necessary dose of FVIII concentrate to reach FVIII target levels after desmopressin administration, will be calculated with a population PK model. The primary endpoint is the proportion of patients reaching FVIII target levels during the first 72 hours after start of the combination treatment. This approach was successfully tested in one pilot patient who received perioperative combination treatment.

Ethics and dissemination:

The DAVID study was approved by the medical ethics committee of the Erasmus MC. Results of the study will be communicated trough publication in international scientific journals and presentation at (inter)national conferences.

Trial registration: NTR5383 (www.trialregister.nl)

For peer review only

Strengths and limitations of this study

- The DAVID-study is a multicentre, prospective trial including patients from all Haemophilia Treatment Centres in the Netherlands.
- Desmopressin and FVIII concentrate combination treatment is an innovative treatment option for non-severe haemophilia A patients.
- By using population pharmacokinetic modelling the dosing of FVIII concentrate may be improved, with less FVIII levels above and below target.
- The DAVID study is a single-armed trial.
- The study population is heterogeneous as all types of surgery and bleeding episodes may be included.

For peer review only

Introduction

Haemophilia A is an X-linked bleeding disorder characterized by a factor VIII (FVIII) deficiency. Non-severe haemophilia A patients (FVIII:C ≥ 0.01-0.40 IU/ml) suffer from bleeding in the perioperative setting and after (minor) trauma. Treatment consists of either desmopressin or FVIII concentrate.

FVIII concentrate is an effective, but expensive treatment option. With current dosing based on body weight, baseline FVIII:C and target FVIII:C, FVIII:C both below (7-45%) and above FVIII (32-81%) target levels have been observed.[1-3] FVIII:C below target may lead to an increased bleeding risk, whereas levels above target increase the costs. Excessively high FVIII:C may also be associated with thrombosis.[4-6] In addition, high FVIII concentrate doses may induce the development of FVIII neutralizing antibodies, causing ineffectiveness of treatment with FVIII concentrate. In cases where antibodies cross-react with patient's endogenous FVIII they can even cause a severe phenotype with spontaneous bleeding.[7-9] Therefore, dosing within the target range with restriction of FVIII concentrate use is important.

An alternative to FVIII concentrate is desmopressin, a synthetic analogue of vasopressin. It releases von Willebrand factor (VWF) and FVIII from the endothelium and thereby improves haemostasis.[10-12] Although treatment is often effective, FVIII:C response to desmopressin exhibits a high variability between haemophilia A patients. Contributing factors reported in literature are age, baseline FVIII:C and the *F8*-gene mutation.[13-15] However, these factors do not explain the observed variability entirely. In addition, in most patients FVIII:C does not increase sufficiently to prevent perioperative bleeding.

Other limitations of desmopressin are the experienced side effects and tachyphylaxis. Most side effects are mild and transient, such as vasodilation. More severe side-effects like hyponatremia are rare and can usually be prevented by a fluid restriction.[16] Tachyphylaxis occurs when repeated dosages of desmopressin are given with short time intervals (12-24 hours). The decrease in FVIII:C response is approximately 30% from the second dose onwards and is believed to be caused by a temporary depletion of VWF and FVIII from the endothelium.[17]

Combined administration of desmopressin and FVIII concentrate may be able to overcome several of the drawbacks of both separate treatment options. However, there is a lack of experience and knowledge with regard to the efficacy and safety of combination treatment. Moreover, optimization of dosing is necessary to get and keep FVIII:C within the target range.

A valuable approach could be pharmacokinetic (PK) guided dosing, where FVIII:C responses of future dosages are predicted based on a population pharmacokinetic model in combination with a limited number (2-3) of FVIII:C measurements, obtained after

For peer review only - http://bmjopen.bmj.com/site/about/guidelines.xhtml

administration of desmopressin and/or FVIII concentrate. This technique was previously shown to be effective in both the prophylactic and perioperative setting in severe and moderate haemophilia A patients.[18-21]

Therefore, we hypothesize that combined PK guided dosing of desmopressin and FVIII concentrate may be a feasible treatment option in non-severe haemophilia A patients with a bleeding episode or undergoing surgery. We aim to prove that the proportion of patients with FVIII:C within the FVIII target ranges can be increased by the use of the combination treatment and PK-guided dosing. In addition, the consumption of FVIII concentrate will be reduced. This innovative approach in haemophilia treatment may be a promising alternative with an increase in quality of care and a concomitant cost reduction.

Methods and analysis

Objectives

The primary objective of the DAVID study is to evaluate if combination treatment of desmopressin and PK-guided dosing of FVIII concentrate is able to increase the proportion of non-severe haemophilia A patients with FVIII:C within the target range during the first 72 hours after start of combination treatment compared to historical controls.

Secondary objectives:

1. To assess FVIII concentrate consumption in all patients.
2. To establish (possible) adverse events of combination treatment; e.g. side effects of desmopressin, bleeding episodes, development of neutralizing antibodies, and thrombotic events.
3. To evaluate the extent of tachyphylaxis after desmopressin treatment.
4. To perform an economical evaluation to quantify the potential cost reduction of the combination treatment.
5. To evaluate the experienced quality of patient care.

Design

The DAVID study is a multicentre single-arm trial in non-severe haemophilia A patients with a bleeding episode or if they need a surgical procedure. Patients will receive combination treatment of desmopressin and FVIII concentrate instead of the conventional treatment which consists of FVIII concentrate monotherapy.

Patient selection

Non-severe haemophilia A patients ≥ 12 years of age with a bleeding episode or undergoing a surgical procedure, requiring FVIII replacement therapy and having a sufficient response to

For peer review only

desmopressin, will be included. Patients will be recruited from one of the haemophilia treatment centres in the Netherlands: Erasmus University Medical Centre Rotterdam, Academic Medical Centre Amsterdam, Leiden University Medical Centre, University Medical Centre Groningen, Radboud university medical centre Nijmegen, University Medical Centre Utrecht, Maxima Medical Centre Eindhoven and Maastricht University Medical Centre.

Inclusion criteria:

- Non-severe haemophilia A patients (FVIII:C ≥0.01 IU/ml)
- Requiring a surgical procedure or having a bleeding episode
- Requiring replacement therapy with FVIII concentrate for at least 48 hours
- Age between 12 and 70 years at study inclusion date
- (Parental) informed consent
- Results of a desmopressin test available (minimal absolute FVIII:C increase >0.2 IU/ml)

Exclusion criteria:

- Patients with other congenital or acquired haemostatic abnormalities
- Inadequate response to desmopressin (absolute increase in FVIII:C <0.2 IU/ml) during a previous desmopressin test
- FVIII neutralizing antibodies (in medical history), unless successfully treated with immunotolerance induction therapy
- Initiation of FVIII concentrate treatment >24 hours before study inclusion
- Patients not eligible for desmopressin treatment due to contraindications, e.g.: intolerance, interactions with co-medication or due to type of surgery

<u>Interventions and study procedures</u>

All patients will receive combination treatment of desmopressin and FVIII concentrate during a bleeding episode or in the perioperative setting for at least 48 hours. All patients will receive a standard dose of desmopressin intravenously (0.3 μg/kg; no maximum dose). In order to combine both medication regimens, an individualized dosing advice for FVIII concentrate will be provided by the clinical pharmacologist. The initial dosing advice will be based on the FVIII:C response observed after a test administration of desmopressin (see below) and previously collected patient and population pharmacokinetic data after administration of desmopressin (endogenous FVIII) and FVIII concentrate (exogenous FVIII). The treating physician states the duration of combination treatment, the mode of administration of FVIII concentrate (continuous or intermittent administration) and determines FVIII target ranges. During combination treatment FVIII:C will be assessed regularly. Accordingly dose adjustments for FVIII concentrate will be made iteratively based on the

For peer review only

results of a desmopressin test administration and using Bayesian analysis. Bayesian analysis will be performed with the NONMEM® software using a dedicated integrated population model describing the PK of FVIII following both the administration of desmopressin and FVII concentrate. As bleeding or acute surgery calls for immediate treatment and constructing a dosing advice takes time, a patient may be included in the DAVID-study until 24 hours after the start of FVIII concentrate monotherapy.

Desmopressin test administration

All patients must have undergone a desmopressin test with at least three FVIII:C measurements. If performed during childhood (<18 years), the test is only admissible when performed ≤4 years before study inclusion. When no desmopressin test has been performed meeting our criteria, the test should be performed with a standard intravenous desmopressin dose of 0.3 µg/kg infused over 30 minutes with a minimum of three FVIII:C measurements: before the administration of desmopressin, 1 hour after desmopressin administration for peak measurement and at least one sample thereafter, for example after 4 hours. All time points of blood sampling and end of desmopressin administration should be documented precisely, as well as exact desmopressin dose and duration of infusion.

Pharmacokinetic (PK) guided dosing

In order to provide an individualized dosing advice, a Bayesian analysis will be performed on the basis of an integrated population PK model. This population model has been constructed based on both endogenous FVIII:C after desmopressin administration and exogenous FVIII:C after FVIII concentrate administration. The population model describes the average PK, including the variability of the FVIII:C response between and within patients following both the administration of desmopressin and the administration of FVIII concentrate. The individualized advice for the first dose of FVIII concentrate will be provided based on this model along with the results of the desmopressin test administration and the perioperative FVIII target levels. Further dosing of FVIII concentrates will be adjusted daily by iterative Bayesian analysis. In this analysis the following information will be included: 1) FVIII release in response to desmopressin after the test dose 2) baseline FVIII:C and 3) measured FVIII:C (both trough and peak levels)(figure 1 and table 1). Dose adjustments based on this iterative Bayesian analysis will be performed in NONMEM® software. All dosing advices will be given one day in advance.

Experienced quality of patient care

To assess experienced quality of care during this innovative intervention, two questionnaires will be given to the patients. Side effects will be assessed using the questionnaire previously

For peer review only - http://bmjopen.bmj.com/site/about/guidelines.xhtml

developed and used by Stoof et al..[16] This questionnaire includes the occurrence of ten different self-reported side effects on a five-point scale. Side effects will be evaluated at two time points: before surgery and three days after surgery. In the second questionnaire (three days after surgery) experienced quality of care will be evaluated with the addition of three questions dedicated to desmopressin and FVIII concentrate combination treatment. Patients will report their satisfaction with the combination treatment on a scale of 1 to 100. They will have the opportunity to explain what is needed to improve the given grade. Finally, they can state their preference for one of the treatment options: FVIII concentrate monotherapy or combination treatment.

Sample size

In the DAVID study, the proportion of patients that reach FVIII target levels with combination treatment in the first 72 hours postoperatively (without adding off-protocol FVIII concentrates) will be assessed. Historical data on current FVIII treatment show a proportion of 0.3 of non-severe haemophilia A patients with FVIII:C within target ranges in this time period.[3] A doubling of this proportion, leading to a proportion of patients of 0.6, is believed to be clinically relevant. To study this with a power of 90% and a two-sided significance level of 0.05, a sample size of minimally 25 patients is needed. In order to allow for dropouts and to overcome the heterogeneity within our study population, we aim to include 50 patients.

Outcome measures

Primary outcome:

The proportion of patients with FVIII trough levels within the FVIII target range during the first 72 hours after start of combination treatment. If a patient has one trough level outside the target range or needs off-protocol FVIII concentrate within the first 72 hours of combination the treatment, the primary endpoint is not reached in that patient. Off-protocol FVIII concentrate is defined as all administered FVIII concentrate outside the PK-guided dosing advice as given by the clinical pharmacologist. FVIII:C will be targeted according to the Dutch Treatment Guideline (Table 2).[17] The treating physician may deviate from the guideline and set different target ranges, based on bleeding phenotype/history or type of surgery.

Secondary outcomes:

- FVIII concentrate consumption, expressed as the total amount of administered units of FVIII concentrate per kilogram per patient;
- Number and nature of adverse events during combined treatment;
- Incidence and severity of bleeding, where the severity of bleeding will be graded according to the ISTH criteria for major and minor bleeding;[22 23]

For peer review only

- Incidence of FVIII neutralizing antibodies;
- Incidence of thrombosis, where thrombosis will be defined according to the Dutch guidelines on thrombosis, myocardial infarctions and strokes;[24 25]
- Incidence and extent of tachyphylaxis, defined as a reduction in the absolute increase in FVIII:C after the second and third desmopressin infusion;
- Medical costs and an economic evaluation;
- Experience quality of patient care, measured by a questionnaire

Data analysis plan

All baseline characteristics will be described as means and standard deviations or medians with interquartile ranges, dependent on whether the parameter is normally distributed. The primary outcome, the proportion of patients within FVIII target ranges, will be analysed by a chi-square test. Retrospective data will be used as a reference, as the DAVID-study only has one study arm.[3] Pharmacokinetic data will be analysed using the NONMEM® software package. Tachyphylaxis of FVIII:C response to desmopressin will be analysed with a paired t-test. Other secondary outcomes will be documented in a descriptive manner, except for the economic analysis.

Economic analysis

An economic evaluation will be performed from a health care perspective from the day of surgery to 90 days postoperatively. The cost-effectiveness of combined treatment will be assessed by calculating the incremental cost-effectiveness ratio (ICER), defined as the difference in costs of combined treatment, compared to usual care, divided by the average change in effectiveness. Actual medical costs will be calculated by multiplying the volumes of healthcare use with the corresponding unit prices. Perioperative data resource use (desmopressin, FVIII concentrates, additional FVIII:C and VWF measurements, PK-profiling) will be collected from medical files. Usual medical costs (to compare to combination treatment costs) will be calculated based on a treatment protocol as it would have been without desmopressin use, taking into account patient's body weight, baseline FVIII:C and type of surgery. The reduction in costs will be represented by the usual medical costs (units of FVIII concentrates) minus the actual medical costs for combined treatment (units of FVIII concentrates, µg of desmopressin, extra FVIII:C measurements). An additional sensitivity analysis will be performed to assess the stability of the results to changes in costs and effectiveness parameters. Primary effectiveness parameter is the proportion of patients reaching FVIII target levels with desmopressin and FVIII concentrate combination treatment. Secondary effectiveness parameter is the frequency of adverse events during combination treatment.

Patent and Public Involvement

During the development of this study we worked closely together with the Netherlands Haemophilia Patient Society (NVHP). One representative of the NVHP is member of the project committee and helped us with the study design. Moreover, 5 members of the NVHP were invited to comment on the patient information. The design and information were adjusted according to their opinions and questions. The final results of the DAVID study will be communicated trough scientific international journals and at international conferences. One major conference may be that of the World Federation of Haemophilia, attended by both physicians, researchers and patients from all over the world. In addition, the results will be communicated in the magazine of the NVHP. Finally, results will also be implemented in the treatment guidelines and patient information will be adjusted accordingly.

Proof of concept

As a proof of concept, one patient was treated with desmopressin and FVIII concentrate combination treatment for the duration of 24 hours. The patient was a 53 year old male with moderate severe haemophilia A (FVIII:C 0.04 IU/ml). His body weight was 100 Kg. He was in need of dental surgery and needed treatment to prevent bleeding. He received an infusion of desmopressin (0.3 µg/kg) over 30 minutes, followed 30 minutes later by FVIII concentrate in a dose of 25 IU/kg (2500 IU). He received a second dose of FVIII concentrate (20 IU/kg; i.e. 2000 IU) in the evening to maintain his FVIII:C above target values. FVIII concentrate dosages were determined using the PK model and based on the patient's response to a previous desmopressin dose and a previous FVIII concentrate administration. Figure 2 shows the measured FVIII:C and the FVIII:C as predicted by the integrated PK model. The measured FVIII:C trough level and peak levels were within 10% of the predicted levels (Figure 2). The patient did not suffer from any other than mild side effects of desmopressin, e.g. flushing and mild tachycardia (101 bpm). To prevent bleeding, he was also treated with tranexamic acid for multiple days. No bleeding was reported before and after the surgery. However, because of a possible infection of the wound he was treated with antibiotics.

Ethics and dissemination

The study was approved by the Medical Ethics Committee of the Erasmus University Medical Centre Rotterdam, the Netherlands. The study will be conducted according to GCP guidelines and the Declaration of Helsinki. Results of the study will be communicated to the (inter)national medical and scientific community trough publication in high-ranking peer reviewed international journals and at (inter)national medical scientific conferences. Results of the study will also be implemented in the Dutch Haemophilia Treatment Guidelines.

For peer review only - http://bmjopen.bmj.com/site/about/guidelines.xhtml

Hopefully the international society of Haemophilia Treatment Centres will adapt the results in their guidelines as well.

Registration

The trial is registered in the Dutch Trial Registry, number NTR5383 (www.trialregister.nl).

Discussion

This prospective trial will allow us to evaluate the safety and efficacy of combination treatment of desmopressin and FVIII concentrate in reaching target FVIII:C during bleeding episodes and in the perioperative setting. Dosing of FVIII concentrate will be determined by an integrated population PK model developed specifically for non-severe haemophilia A patients to be treated with combination treatment. Fifty non-severe haemophilia A patients will be included from all haemophilia treatment centres in the Netherlands to reach our aim.

The DAVID study has some limitations. First, this study is not designed as a randomized controlled trial; it does not include a control group. Therefore, no direct comparison to standard treatment will be possible. However, we performed an extensive retrospective cohort study to determine the effectiveness of current clinical practice in which 37 patients undergoing 52 surgeries, were evaluated.[3] Moreover, the amount of FVIII concentrate that would have been administered to the patients included in the DAVID study as if desmopressin was not administered and as if no PK-guided dosing was applied, will be calculated.

The second limitation is the heterogeneity of the study population. All types of surgery, unless not compatible with desmopressin treatment, may be included in the study. To limit the effects of this heterogeneity, only patients with an expected treatment duration of at least 48 hours will be included. Moreover, the data that were used to develop the integrated population PK model also included data from various types of surgery.

Conclusion

In the DAVID study, efficacy and safety of desmopressin and FVIII concentrate combination treatment in non-severe haemophilia A patients will be determined. Using this innovative approach treatment of non-severe haemophilia A patients both during bleeding episodes and in the perioperative setting may be improved.

For peer review only

Authors' contribution

MK, MCn, FL and RM designed the study and critically revised the manuscript. LS, SP and RH wrote the manuscript and refined the study design. MD, KF, EB, MCo, JE, BL-G, KM, LN and EM critically revised the manuscript and refined the study design.
We would like to specially thank MD for her contribution on behalf of the NVHP and the five NVHP members who helped to improve the patient information. In addition we would like to thank the pilot patient for his cooperation and for letting us use his case as an example.

Funding statement

This work was supported by ZonMw, (grant number 836031003), the Dutch organisation for Health Research and Development.

Competing interest statement

L.M. Schütte; received reimbursement from CSL-Behring for attending a symposium, not related to this study.

M.H. Cnossen; received unrestricted research/educational funding for various projects as well as travel fees from the following institutions and companies: ZonMW, Innovatiefonds, Pfizer, Baxalta/Shire, Bayer Schering Pharma, Novo Nordisk, Novartis, Roche and CSL Behring, all not related to this study.

R.M. van Hest, E.A.M. Beckers, M. Coppens, M. Driessens, L. Nieuwenhuizen, S.Polinder: nothing to disclose relevant to the DAVID study.

K. Fijnvandraat: is a member of the European Hemophilia Treatment and Standardization Board sponsored by Baxter, has received unrestricted research grants from CSL Behring and Bayer, and has given lectures at educational symposiums organized by Pfizer, Bayer and Baxter.

J. Eikenboom: received research funding from CSL Behring and honorarium for educational activity from Roche, not related to this study.

B.A.P. Laros-van Gorkom: received unrestricted educational grants from Baxter and CSL Behring, and speaker fees from Sanquin**.**

K. Meijer: research support from Bayer, Sanquin and Pfizer; speaker fees from Bayer, Sanquin, Boehringer Ingelheim, BMS and Aspen; consulting fees from Uniqure, not related to this study

F.W.G. Leebeek; received unrestricted research grants from CSL-Behring and Baxalta/Shire not related to this study. He is consulant for Shire, NovoNordisk and UniQure. Fees go to the university.

R.A.A. Mathôt: received personal fees from Merck Sharp & Dohme and Zeria and grants from Bayer, UCB Pharma and Hoffman La Roche with no involvement in this study.

For peer review only

M.J.H.A.Kruip; received unrestricted research grants from Pfizer, Innovatiefonds and Ferring with no involvement in this study.

References

1. Hazendonk HC, Lock J, Mathot RA, et al. Under dosing and overdosing of factor VIII in the perioperative setting in haemophilia a: A need for optimization of dosing. Haemophilia 2015;**21**((Hazendonk H.C.; Lock J.; Bouzariouh H.; Cnossen M.H.) Department of Pediatric Hematology, Erasmus University Medical Centre, Sophia Childrens Hospital, Rotterdam, Netherlands):81-82.
2. Hazendonk HC, Lock J, Mathot RA, et al. Perioperative treatment of hemophilia A patients: blood group O patients are at risk of bleeding complications. J Thromb Haemost 2016;**14**(3):468-78.
3. Schütte LM, de Rooij N, Hazendonk HCAM, et al. Perioperative FVIII Concentrate Treatment in Mild Hemophilia a Patients Shows a High Rate of Overdosing - David/Opti-Clot Studies. Blood 2015;**126**(23):3510-10.
4. Ritchie B, Woodman RC, Poon MC. Deep venous thrombosis in hemophilia A. Am J Med 1992;**93**(6):699-700.
5. Escuriola Ettingshausen C, Martinez Saguer I, Kreuz W. Portal vein thrombosis in a patient with severe haemophilia A and F V G1691A mutation during continuous infusion of F VIII after intramural jejunal bleeding - Successful thrombolysis under heparin therapy. European Journal of Pediatrics, Supplement 1999;**158**(3):S180-S82.
6. Russell Z, Riconda D, Pollack L, et al. Thrombosis in a pregnant hemophilia A carrier after intrapartum recombinant factor VIII. Obstet Gynecol 2005;**105**(4):875-6.
7. Eckhardt CL, Mauser-Bunschoten EP, Peters M, et al. Inhibitor incidence after intensive FVIII replacement for surgery in mild and moderate haemophilia A: a prospective national study in the Netherlands. Br J Haematol 2012;**157**(6):747-52.
8. Astermark J. FVIII inhibitors: pathogenesis and avoidance. Blood 2015;**125**(13):2045-51.
9. d'Oiron R, Pipe SW, Jacquemin M. Mild/moderate haemophilia A: new insights into molecular mechanisms and inhibitor development. Haemophilia 2008;**14 Suppl 3**:138-46.
10. Kaufmann JE, Vischer UM. Cellular mechanisms of the hemostatic effects of desmopressin (DDAVP). J Thromb Haemost 2003;**1**(4):682-9.
11. Mannucci. Desmopressin (DDAVP) in the treatment of bleeding disorders. World federation of hemophilia 2012.
12. Pan J, Dinh TT, Rajaraman A, et al. Patterns of expression of factor VIII and von Willebrand factor by endothelial cell subsets in vivo. Blood 2016.
13. Stoof SC, Sanders YV, Petrij F, et al. Response to desmopressin is strongly dependent on F8 gene mutation type in mild and moderate haemophilia A. Thromb Haemost 2013;**109**(3):440-9.
14. Nance D, Fletcher SN, Bolgiano DC, et al. Factor VIII mutation and desmopressin-responsiveness in 62 patients with mild haemophilia A. Haemophilia 2013;**19**(5):720-6.
15. Castaman G, Mancuso ME, Giacomelli SH, et al. Molecular and phenotypic determinants of the response to desmopressin in adult patients with mild hemophilia A. J Thromb Haemost 2009;**7**(11):1824-31.
16. Stoof SC, Cnossen MH, de Maat MP, et al. Side effects of desmopressin in patients with bleeding disorders. Haemophilia 2016;**22**(1):39-45.
17. Mannucci PM, Bettega D, Cattaneo M. Patterns of development of tachyphylaxis in patients with haemophilia and von Willebrand disease after repeated doses of desmopressin (DDAVP). Br J Haematol 1992;**82**(1):87-93.
18. Björkman S. Limited blood sampling for pharmacokinetic dose tailoring of FVIII in the prophylactic treatment of haemophilia A. Haemophilia 2010;**16**(4):597-605.
19. Carlsson M, Berntorp E, Bjorkman S, et al. Improved cost-effectiveness by pharmacokinetic dosing of factor VIII in prophylactic treatment of haemophilia A. Haemophilia 1997;**3**(2):96-101.

For peer review only

20. Hazendonk H, Fijnvandraat K, Lock J, et al. A population pharmacokinetic model for perioperative dosing of factor VIII in hemophilia A patients. Haematologica 2016.
21. Hazendonk HC, van Moort I, Fijnvandraat K, et al. The "OPTI-CLOT" trial. A randomised controlled trial on periOperative PharmacokineTIc-guided dosing of CLOTting factor concentrate in haemophilia A. Thromb Haemost 2015;**114**(3):639-44.
22. Kaatz S, Ahmad D, Spyropoulos AC, et al. Definition of clinically relevant non-major bleeding in studies of anticoagulants in atrial fibrillation and venous thromboembolic disease in non-surgical patients: communication from the SSC of the ISTH. J Thromb Haemost 2015;**13**(11):2119-26.
23. Schulman S, Kearon C, Subcommittee on Control of Anticoagulation of the S, et al. Definition of major bleeding in clinical investigations of antihemostatic medicinal products in non-surgical patients. J Thromb Haemost 2005;**3**(4):692-4.
24. Kwaliteitsinstituut voor de Gezondheidszorg, C. B. O. *Diagnostiek, preventie en behandeling van veneuze trombo-embolie en secundaire preventie van arteriële trombose*. Alphen aan den Rijn: Van Zuiden Communications, 2008.
25. Kwaliteitsinstituut voor de Gezondheidszorg CBO. *Diagnostiek, behandeling en zorg voor patienten met een beroerte : richtlijn*. Utrecht: Nederlandse Vereniging voor Neurologie; Utrecht: Kwaliteitsinstituut voor de Gezondheidszorg CBO, 2008.
26. Leebeek FWG, Mauser-Bunschoten EP. *Diagnostiek en behandeling van hemofilie en aanverwante hemostasestoornissen : richtlijn*. Alphen aan den Rijn: Van Zuiden Communications, 2009.

For peer review only

Legends to tables and figures

Table 1: Overview of blood sampling for FVIII:C measurements

Grey: obligatory measurements

† only in case of elective surgery, otherwise before first desmopressin administration

‡ pre = before desmopressin, post = after desmopressin, peak = after FVIII-concentrate, post-treat = after surgery; in case of bleeding; 2-6 hours after desmopressin, trough = before next dose of FVIII-concentrate

‡ pre = before desmopressin, post = after desmopressin, peak = after loading dose FVIII-concentrate, post-treat = after surgery; in case of bleeding: 2-6 hours after desmopressin, pre-adjust = before dosage , steady state = FVIII-measurement at random time point

FVIII measurements are shown here until day 3 after start of combination treatment. Monitoring will continue if treatment is still necessary.

Table 2: Target ranges for FVIII:C in IU/ml in the perioperative setting[26]

Figure 1: Flowchart of study

Figure 2: FVIII:C course after desmopressin and FVIII concentrate combination treatment in a pilot patient with moderate haemophilia A (FVIII:C 0.04 IU/ml). T_0 = preoperative desmopressin infusion. Lines are the predicted FVIII:C. Predictions were based on a previous desmopressin test dose and FVIII concentrate administration, prior to study inclusion. Solid line is total FVIII:C and can be measured. Open circles are measured FVIII:C. Infusions of desmopressin and FVIII concentrate (FVIII con.) are depicted with arrows.

For peer review only

Table 1: Overview of blood sampling for FVIII:C measurements

	Within 4 weeks before surgery†	D0 = Day of first desmopressin infusion				D1				D2		D3	**4-8 weeks after FVIII treatment**
Time – bolus infusions‡	baseline	pre	post	peak	post-treat	pre	post	peak	pre	post	peak	trough	
Time – continuous infusion‡	baseline	pre	post	peak	post-treat	pre	post	pre-adjust	pre	post	pre-adjust	steady state	
Primary endpoint						X			X			X	
Sodium	X	X				X			X			X	
Neutralizing antibodies	X												X

Grey: obligatory measurements

† only in case of elective surgery, otherwise before first desmopressin administration

‡ pre = before desmopressin, post = after desmopressin, peak = after FVIII-concentrate, post-treat = after surgery; in case of bleeding; 2-6 hours after desmopressin, trough = before next dose of FVIII-concentrate

‡ pre = before desmopressin, post = after desmopressin, peak = after loading dose FVIII-concentrate, post-treat = after surgery; in case of bleeding: 2-6 hours after desmopressin, pre-adjust = before dosage , steady state = FVIII-measurement at random time point

FVIII measurements are shown here until day 3 after start of combination treatment. Monitoring will continue if treatment is still necessary.

Table 2: Target ranges for FVIII:C in IU/ml in the perioperative setting[26]

**Time**	**FVIII target level (IU/ml)**
**Day 0 (hour 0-24)**	0.8-1.0 (peak)
**Day 1-4 (hour 24-96)**	0.5-0.8 (trough)
**Day ≥ 5 (hour > 96)**	0.3-0.5 (trough)

For peer review only

For peer review only - http://bmjopen.bmj.com/site/about/guidelines.xhtml

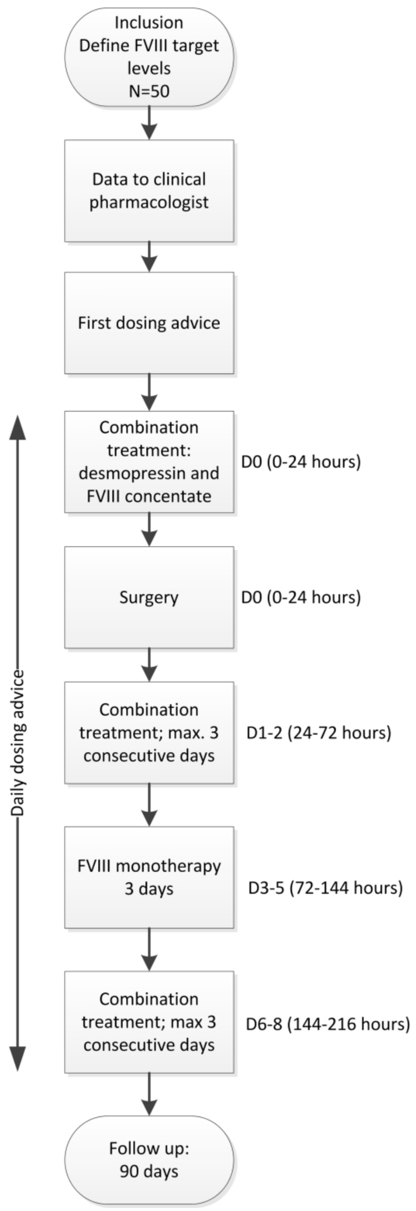

Figure 1: Flowchart of study

177x519mm (300 x 300 DPI)

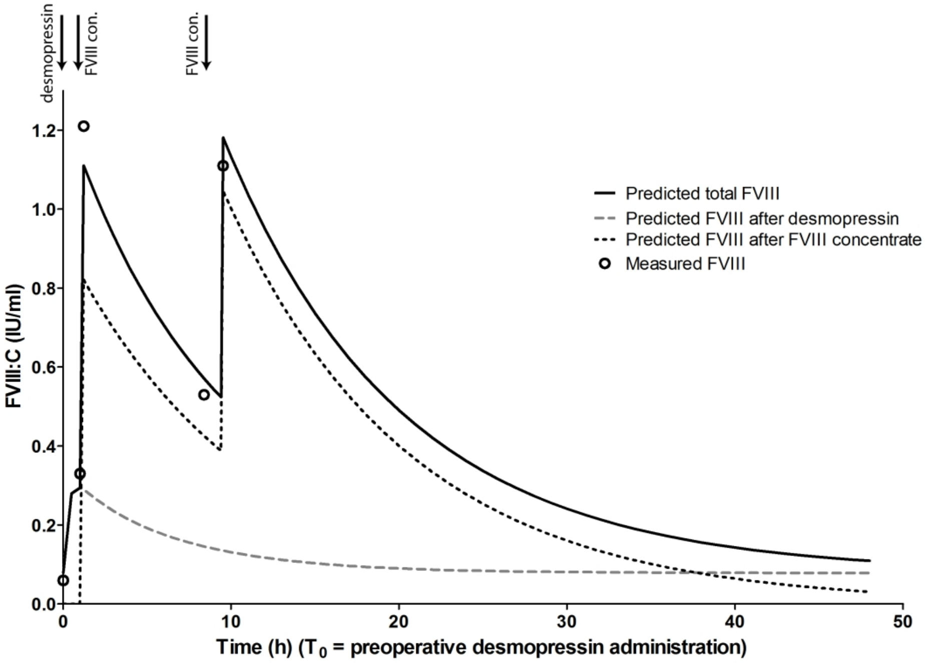

Figure 2: FVIII:C course after desmopressin and FVIII concentrate combination treatment in a pilot patient with moderate haemophilia A (FVIII:C 0.04 IU/ml). T0 = preoperative desmopressin infusion. Lines are the predicted FVIII:C. Predictions were based on a previous desmopressin test dose and FVIII concentrate administration, prior to study inclusion. Solid line is total FVIII:C and can be measured. Open circles are measured FVIII:C. Infusions of desmopressin and FVIII concentrate (FVIII con.) are depicted with arrows.

246x171mm (300 x 300 DPI)

DAVID - Study

Revised Standards for Quality Improvement Reporting Excellence (SQUIRE 2.0)
September 15, 2015

Text Section and Item Name	Section or Item Description
**Notes to authors**	• The SQUIRE guidelines provide a framework for reporting new knowledge about how to improve healthcare<br>• The SQUIRE guidelines are intended for reports that describe <u>system</u> level work to improve the quality, safety, and value of healthcare, and used methods to establish that observed outcomes were due to the <u>intervention(s)</u>.<br>• A range of approaches exists for improving healthcare. SQUIRE may be adapted for reporting any of these.<br>• Authors should consider every SQUIRE item, but it may be inappropriate or unnecessary to include every SQUIRE element in a particular manuscript.<br>• The SQUIRE Glossary contains definitions of many of the key words in SQUIRE.<br>• The Explanation and Elaboration document provides specific examples of well-written SQUIRE items, and an in-depth explanation of each item.<br>• Please cite SQUIRE when it is used to write a manuscript.
**Title and Abstract**	
**1. Title**	Indicate that the manuscript concerns an <u>initiative</u> to improve healthcare (broadly defined to include the quality, safety, effectiveness, patient-centeredness, timeliness, cost, efficiency, and equity of healthcare) ✓
**2. Abstract**	a. Provide adequate information to aid in searching and indexing ✓<br>b. Summarize all key information from various sections of the text using the abstract format of the intended publication or a structured summary such as: background, local <u>problem</u>, methods, interventions, results, conclusions ✓
**Introduction**	*Why did you start?*
**3. <u>Problem</u> <u>Description</u>**	Nature and significance of the local <u>problem</u> ✓
**4. Available knowledge**	Summary of what is currently known about the <u>problem</u>, including relevant previous studies ✓

5. Rationale	Informal or formal frameworks, models, concepts, and/or theories used to explain the problem, any reasons or assumptions that were used to develop the intervention(s), and reasons why the intervention(s) was expected to work ✓
6. Specific aims	Purpose of the project and of this report ✓
**Methods**	*What did you do?*
7. Context	Contextual elements considered important at the outset of introducing the intervention(s) ✓
8. Intervention(s)	a. Description of the intervention(s) in sufficient detail that others could reproduce it ✓<br>b. Specifics of the team involved in the work ✓
9. Study of the Intervention(s)	a. Approach chosen for assessing the impact of the intervention(s) ✓<br>b. Approach used to establish whether the observed outcomes were due to the intervention(s) ✓
10. Measures	a. Measures chosen for studying processes and outcomes of the intervention(s), including rationale for choosing them, their operational definitions, and their validity and reliability ✓<br>b. Description of the approach to the ongoing assessment of contextual elements that contributed to the success, failure, efficiency, and cost na<br>c. Methods employed for assessing completeness and accuracy of data ✓
11. Analysis	a. Qualitative and quantitative methods used to draw inferences from the data ✓<br>b. Methods for understanding variation within the data, including the effects of time as a variable ✓
12. Ethical Considerations	Ethical aspects of implementing and studying the intervention(s) and how they were addressed, including, but not limited to, formal ethics review and potential conflict(s) of interest ✓
**Results**	*What did you find?* NA
13. Results	a. Initial steps of the intervention(s) and their evolution over time (*e.g.*, time-line diagram, flow chart, or table), including modifications made to the intervention during the project na<br>b. Details of the process measures and outcome ✓<br>c. Contextual elements that interacted with the intervention(s)<br>d. Observed associations between outcomes, interventions, and relevant contextual elements na<br>e. Unintended consequences such as unexpected benefits, problems, failures, or costs associated with the intervention(s). na<br>f. Details about missing data na
**Discussion**	*What does it mean?*
14. Summary	a. Key findings, including relevance to the rationale and specific aims na<br>b. Particular strengths of the project ✓

**15. Interpretation**	a. Nature of the association between the intervention(s) and the outcomes na<br>b. Comparison of results with findings from other publications na<br>c. Impact of the project on people and systems ✓<br>d. Reasons for any differences between observed and anticipated outcomes, including the influence of context na<br>e. Costs and strategic trade-offs, including opportunity costs na
**16. Limitations**	a. Limits to the generalizability of the work ✓<br>b. Factors that might have limited internal validity such as confounding, bias, or imprecision in the design, methods, measurement, or analysis ✓<br>c. Efforts made to minimize and adjust for limitations ✓
**17. Conclusions**	a. Usefulness of the work ✓<br>b. Sustainability ✓<br>c. Potential for spread to other contexts na<br>d. Implications for practice and for further study in the field na<br>e. Suggested next steps na
**Other information**	
**18. Funding**	Sources of funding that supported this work. Role, if any, of the funding organization in the design, implementation, interpretation, and reporting ✓

For peer review only

BMJ Open

Desmopressin treatment combined with clotting factor VIII concentrates in patients with non-severe haemophilia A: protocol for a multicentre single-armed trial, the DAVID study

Journal:	*BMJ Open*
Manuscript ID	bmjopen-2018-022719.R1
Article Type:	Protocol
Date Submitted by the Author:	16-Jul-2018

Complete List of Authors:	Schütte, Lisette; Erasmus MC, Haematology<br>Cnossen, Marjon; Erasmus MC Sophia, Pediatric Hematology<br>Van Hest, Reinier; AMC, Hospital Pharmacy<br>Driessens, Mariette; Netherlands Haemophilia Patient Society<br>Fijnvandraat, Karin; Emma Kinderziekenhuis AMC, Pediatric Hematology; Sanquin Research, Plasma Proteins<br>Polinder, Suzanne; Erasmus MC, Public Health<br>Beckers, Erik; Maastricht University Medical Centre, Haematology<br>Coppens, Michiel; Academic Medical Center, Vascular Medicine<br>Eikenboom, Jeroen; Leids Universitair Medisch Centrum, Thrombosis and Haemostasis<br>Laros-van Gorkom, Britta; Radboudumc, Haematology<br>Meijer, Karina; Universitair Medisch Centrum Groningen, Department of Haematology<br>Nieuwenhuizen, Laurens; Maxima Medisch Centrum, Haematology<br>Mauser-Bunschoten, Evelien; University Medical Centre Utrecht, Van Creveldclinic<br>Leebeek, Frank; Erasmus MC, Hematology<br>Mathôt, Ron; Academic Medical Center, Hospital Pharmacy<br>Kruip, Marieke; Erasmus MC, Hematology

**Primary Subject Heading**:	Haematology (incl blood transfusion)
Secondary Subject Heading:	Patient-centred medicine, Pharmacology and therapeutics, Surgery
Keywords:	Haemophilia A, Desmopressin, FVIII concentrate, SURGERY, Pharmacokinetic modelling

SCHOLARONE™
Manuscripts

Desmopressin treatment combined with clotting factor VIII concentrates in patients with non-severe haemophilia A: protocol for a multicentre single-armed trial, the DAVID study

L.M. Schütte[1], M.H. Cnossen[2], R.M. van Hest[3], M.H.E. Driessens[4], K. Fijnvandraat[5], S. Polinder[6], E.A.M. Beckers[7], M. Coppens[8], J. Eikenboom[9], B.A.P. Laros-van Gorkom[10], K. Meijer[11], L. Nieuwenhuizen[12], E.P. Mauser-Bunschoten[13], F.W.G. Leebeek[1], R.A.A. Mathôt[3], M.J.H.A. Kruip[1]

Corresponding Author:
Dr. Marieke J.H.A. Kruip
Room Na-823
P.O. Box 2040
3000 CA Rotterdam
The Netherlands
e-mail: m.kruip@erasmusmc.nl
telephone number: +31 10 7033123

Affiliations
[1]Department of Haematology, Erasmus University Medical Centre, Rotterdam, the Netherlands; [2]Department of Paediatric Haematology, Erasmus University Medical Centre - Sophia Children's Hospital, Rotterdam, the Netherlands; [3]Department of Hospital Pharmacy, Academic Medical Centre, Amsterdam, the Netherlands; [4]Netherlands Haemophilia Patient Society, Nijkerk, the Netherlands; [5]Department of Paediatric Haematology, Academic Medical Centre, Amsterdam, The Netherlands; Department of Plasma Proteins, Sanquin Research, Amsterdam, The Netherlands; [6]Department of Public Health, Erasmus University Medical Centre, Rotterdam, the Netherlands; [7]Department of Haematology, Maastricht University Medical Centre, Maastricht, the Netherlands; [8]Department of Vascular Medicine, Academic Medical Centre, Amsterdam, the Netherlands; [9]Department of Thrombosis and Haemostasis, Leiden University Medical Centre, Leiden, the Netherlands; [10]Department of Haematology, Radboud University Medical Centre, Nijmegen, the Netherlands; [11]Department of Haematology, University Medical Centre Groningen, Groningen, the Netherlands; [12]Department of Haematology, Maxima Medical Centre, Eindhoven, the Netherlands; [13]Van Creveldkliniek, Utrecht, the Netherlands

Word count: 3710

For peer review only

Abstract

Introduction:

Haemophilia A is an inherited bleeding disorder characterised by factor VIII (FVIII) deficiency. In non-severe haemophilia A patients, surgery and bleeding are the main indications for treatment with FVIII concentrate. A recent study reported that standard dosing frequently results in FVIII levels (FVIII:C) below or above FVIII target ranges, leading to respectively a bleeding risk or excessive costs. In addition, FVIII concentrate treatment carries a risk of development of neutralizing antibodies.

An alternative is desmopressin, which releases endogenous FVIII and von Willebrand factor. In most non-severe haemophilia A patients, desmopressin alone is not enough to achieve FVIII target levels during surgery or bleeding. We hypothesize that combined pharmacokinetic (PK) guided administration of desmopressin and FVIII concentrate may improve dosing accuracy and reduces FVIII concentrate consumption.

Methods and analysis:

In the DAVID study, fifty non-severe haemophilia A patients (FVIII:C ≥0.01 IU/ml) with a bleeding episode or undergoing surgery will receive desmopressin and FVIII concentrate combination treatment. The necessary dose of FVIII concentrate to reach FVIII target levels after desmopressin administration, will be calculated with a population PK model. The primary endpoint is the proportion of patients reaching FVIII target levels during the first 72 hours after start of the combination treatment. This approach was successfully tested in one pilot patient who received perioperative combination treatment.

Ethics and dissemination:

The DAVID study was approved by the medical ethics committee of the Erasmus MC. Results of the study will be communicated trough publication in international scientific journals and presentation at (inter)national conferences.

Trial registration: NTR5383 (www.trialregister.nl), EudraCT: 2014-00535-14

For peer review only

Strengths and limitations of this study

- The DAVID-study is a multicentre, prospective trial including patients from all Haemophilia Treatment Centres in the Netherlands.
- Desmopressin and FVIII concentrate combination treatment is an innovative treatment option for non-severe haemophilia A patients.
- By using maximum *aposteriori* Bayesian estimation based on an integrated population pharmacokinetic model and measured FVIII levels, the dosing of FVIII concentrate may be improved, with less FVIII levels above and below target.
- The DAVID study is a single-armed trial.
- The study population is heterogeneous as all types of surgery and bleeding episodes may be included.

For peer review only

Introduction

Haemophilia A is an X-linked bleeding disorder characterized by a factor VIII (FVIII) deficiency. Non-severe haemophilia A patients (FVIII:C ≥ 0.01-0.40 IU/ml) suffer from bleeding in the perioperative setting and after (minor) trauma. Treatment consists of either desmopressin or FVIII concentrate.

FVIII concentrate is an effective, but expensive treatment option. With current dosing based on body weight, baseline FVIII:C and target FVIII:C, FVIII:C both below (7-45%) and above FVIII (32-81%) target levels have been observed.[1-3] FVIII:C below target may lead to an increased bleeding risk, whereas levels above target increase the costs. Excessively high FVIII:C may also be associated with thrombosis.[4-6] In addition, high FVIII concentrate doses may induce the development of FVIII neutralizing antibodies, causing ineffectiveness of treatment with FVIII concentrate. In cases where antibodies cross-react with patient's endogenous FVIII they can even cause a severe phenotype with spontaneous bleeding.[7-9] Therefore, dosing within the target range with restriction of FVIII concentrate use is important.

An alternative to FVIII concentrate is desmopressin, a synthetic analogue of vasopressin. It releases von Willebrand factor (VWF) and FVIII from the endothelium and thereby improves haemostasis.[10-12] Although treatment is often effective, FVIII:C response to desmopressin exhibits a high variability between haemophilia A patients. Contributing factors reported in literature are age, baseline FVIII:C and the *F8*-gene mutation.[13-15] However, these factors do not explain the observed variability entirely. In addition, in most patients FVIII:C does not increase sufficiently to prevent perioperative bleeding.

Other limitations of desmopressin are the experienced side effects and tachyphylaxis. Most side effects are mild and transient, such as vasodilation. More severe side-effects like hyponatremia are rare and can usually be prevented by a fluid restriction.[16] Tachyphylaxis occurs when repeated dosages of desmopressin are given with short time intervals (12-24 hours). The decrease in FVIII:C response is approximately 30% from the second dose onwards and is believed to be caused by a temporary depletion of VWF and FVIII from the endothelium.[17]

Combined administration of desmopressin and FVIII concentrate may be able to overcome several of the drawbacks of both separate treatment options. However, there is a lack of experience and knowledge with regard to the efficacy and safety of combination treatment. Moreover, optimization of dosing is necessary to get and keep FVIII:C within the target range.

A valuable approach could be pharmacokinetic (PK) guided dosing, where FVIII:C responses of future dosages are predicted based on a population pharmacokinetic model in

For peer review only

combination with a limited number (2-3) of FVIII:C measurements, obtained after administration of desmopressin and/or FVIII concentrate. This technique was previously shown to be effective in both the prophylactic and perioperative setting in severe and moderate haemophilia A patients.[18-21]

Therefore, we hypothesize that combined PK guided dosing of desmopressin and FVIII concentrate may be a feasible treatment option in non-severe haemophilia A patients with a bleeding episode or undergoing surgery. Our aim is to show that the proportion of patients with FVIII:C within the FVIII target ranges can be increased by the use of the combination treatment and PK-guided dosing. In addition, the consumption of FVIII concentrate will be reduced. This innovative approach in haemophilia treatment may be a promising alternative with an increase in quality of care and a concomitant cost reduction.

Methods and analysis

<u>Objectives</u>

The primary objective of the DAVID study is to evaluate if combination treatment of desmopressin and PK-guided dosing of FVIII concentrate is able to increase the proportion of non-severe haemophilia A patients with FVIII:C within the target range during the first 72 hours after start of combination treatment compared to historical controls.

Secondary objectives:

1. To assess FVIII concentrate consumption in all patients.
2. To acquire data to improve the integrated population PK model for desmopressin and FVIII concentrate combination treatment
3. To establish (possible) adverse events of combination treatment; e.g. side effects of desmopressin, bleeding episodes, development of neutralizing antibodies, and thrombotic events.
4. To evaluate the extent of tachyphylaxis after desmopressin treatment.
5. To perform an economical evaluation to quantify the potential cost reduction of the combination treatment.
6. To evaluate the experienced quality of patient care.

<u>Design</u>

The DAVID study is a multicentre single-arm trial in non-severe haemophilia A patients with a bleeding episode or if they need a surgical procedure. Patients will receive combination

For peer review only

treatment of desmopressin and FVIII concentrate instead of the conventional treatment which consists of FVIII concentrate monotherapy.

Patient selection and recruitment

Non-severe haemophilia A patients ≥ 12 years of age with a bleeding episode or undergoing a surgical procedure, requiring FVIII replacement therapy and having a sufficient response to desmopressin, will be included. Patients will be recruited from one of the following haemophilia treatment centres in the Netherlands: Erasmus University Medical Centre Rotterdam, Academic Medical Centre Amsterdam, Leiden University Medical Centre, University Medical Centre Groningen, Radboud university medical centre Nijmegen, University Medical Centre Utrecht, Maxima Medical Centre Eindhoven and Maastricht University Medical Centre. They will be approached by telephone or during a visit to the (outpatient)_clinic when they present with a bleeding or need a surgical procedure.

Inclusion criteria:

- Non-severe haemophilia A patients (FVIII:C ≥0.01 IU/ml)
- Requiring a surgical procedure or having a bleeding episode
- Requiring replacement therapy with FVIII concentrate for at least 48 hours
- Age between 12 and 70 years at study inclusion date
- (Parental) informed consent
- Results of a desmopressin test available (minimal absolute FVIII:C increase >0.2 IU/ml)

Exclusion criteria:

- Patients with other congenital or acquired haemostatic abnormalities
- Inadequate response to desmopressin (absolute increase in FVIII:C <0.2 IU/ml) during a previous desmopressin test
- FVIII neutralizing antibodies (in medical history), unless successfully treated with immunotolerance induction therapy
- Initiation of FVIII concentrate treatment >24 hours before study inclusion
- Patients not eligible for desmopressin treatment due to contraindications, e.g.: intolerance, interactions with co-medication or due to type of surgery

For peer review only

Interventions and study procedures

All patients will receive combination treatment of desmopressin and FVIII concentrate during a bleeding episode or in the perioperative setting for at least 48 hours. All patients will receive a standard dose of desmopressin intravenously (0.3 μg/kg; no maximum dose). In order to combine both medication regimens, an individualized dosing advice for FVIII concentrate will be provided by the clinical pharmacologist. The initial dosing advice will be based on the FVIII:C response observed after a test administration of desmopressin (see below) and previously collected patient and population pharmacokinetic data after administration of desmopressin (endogenous FVIII) and FVIII concentrate (exogenous FVIII). The treating physician states the duration of combination treatment, the mode of administration of FVIII concentrate (continuous or intermittent administration) and determines FVIII target ranges. During combination treatment FVIII:C will be assessed regularly. Accordingly dose adjustments for FVIII concentrate will be made iteratively based on the results of a desmopressin test administration and using Bayesian analysis. Bayesian analysis will be performed with the NONMEM® software using a dedicated integrated population model describing the PK of FVIII following both the administration of desmopressin and FVII concentrate. As bleeding or acute surgery calls for immediate treatment and constructing a dosing advice takes time, a patient may be included in the DAVID-study until 24 hours after the start of FVIII concentrate monotherapy.

Adherence to the study protocol will be improved by the use of a separate script per included patient, in which an approximated timeline, contact details and all responsibilities of the involved research and treatment team are written down.

Concomitant treatment

As patients receive desmopressin, they will have a fluid restriction of 1.5 L per 24 hours, till 24 hours after the last desmopressin administration. Furthermore, all concomitant treatment and therapeutics are allowed, except for the therapeutics specified in the exclusion criteria. This includes other treatment than desmopressin or factor concentrate necessary to prevent or treat bleeding, such as tranexamic acid. The same will apply for prophylactic treatment to prevent thrombosis. These treatment options will be applied at the discretion of the treating physician and will be documented in all patients.

Desmopressin test administration

All patients must have undergone a desmopressin test with at least three FVIII:C measurements. If performed during childhood (<18 years), the test is only admissible when

For peer review only

performed ≤4 years before study inclusion. When no desmopressin test has been performed meeting these criteria, the test should be performed with a standard intravenous desmopressin dose of 0.3 µg/kg infused over 30 minutes with a minimum of three FVIII:C measurements: before the administration of desmopressin, 1 hour after desmopressin administration for peak measurement and at least one sample thereafter, for example after 4 hours. All time points of blood sampling and end of desmopressin administration should be documented precisely, as well as exact desmopressin dose and duration of infusion.

Pharmacokinetic (PK) guided dosing

In order to provide an individualized dosing advice, a Bayesian analysis will be performed on the basis of an integrated population PK model. This population model has been constructed based on two population pharmacokinetic models: 1) a population PK model of FVIII:C response after desmopressin administration [22] and 2) a population PK model of FVIII:C after administration of FVIII concentrate.[22] This latter model was constructed based on perioperative data from 29 non-severe, adult haemophilia A patients from whom 245 FVIII:C measurements were available The final model estimated the baseline FVIII:C to which estimated FVIII:C, that followed administration of FVIII concentrate, were added according to a one-compartment model with first-order elimination. Two covariates could be identified after multivariate regression analysis: VWF:Ag had a negative association with clearance and the most recently measured FVIII:C had a positive association with the estimated baseline. The within-patient variability (variability between different treatment episodes with FVIII concentrate) was 51.2% on baseline FVIII:C. In table 1 the most important model characteristics are shown.

The integrated population PK model, used in this study, describes the average PK, including the variability of the baseline FVIII:C and FVIII:C response between and within patients following both the administration of desmopressin and the administration of FVIII concentrate. This model will be used for both the perioperative setting and around bleeding episodes. The individualized dosing advice for the first dose of FVIII concentrate will be provided based on this model along with the results of the desmopressin test administration and the perioperative FVIII target levels. Further dosing of FVIII concentrates will be adjusted daily by iterative maximum *aposteriori* Bayesian analysis based on the population PK model in conjunction with perioperatively measured FVIII:C. In this analysis the following information will be included: 1) FVIII release in response to desmopressin after the test dose 2) baseline FVIII:C and 3) measured FVIII:C (both trough and peak levels)(figure 1 and table 2). Dose adjustments based on this iterative

Bayesian analysis will be performed in NONMEM® software. All dosing advices will be given one day in advance.

Experienced quality of patient care

To assess experienced quality of care during this innovative intervention, two questionnaires will be given to the patients. Side effects will be assessed using the questionnaire previously developed and used by Stoof et al..[16] This questionnaire includes the occurrence of ten different self-reported side effects on a five-point scale. Side effects will be evaluated at two time points: before surgery and three days after surgery. In the second questionnaire (three days after surgery) experienced quality of care will be evaluated with the addition of three questions dedicated to desmopressin and FVIII concentrate combination treatment. Patients will report their satisfaction with the combination treatment on a scale of 1 to 100. They will have the opportunity to explain what is needed to improve the given grade. Finally, they can state their preference for one of the treatment options: FVIII concentrate monotherapy or combination treatment. The last question will be only asked to patients who have a previous experience with FVIII concentrate monotherapy.

Sample size

In the DAVID study, the proportion of patients that reach FVIII target levels with combination treatment in the first 72 hours postoperatively (without adding off-protocol FVIII concentrates) will be assessed. Historical data on current FVIII treatment show a proportion of 0.3 of non-severe haemophilia A patients with FVIII:C within target ranges in this time period.[3] A doubling of this proportion, leading to a proportion of patients of 0.6, is believed to be clinically relevant. To study this with a power of 90% and a two-sided significance level of 0.05, a sample size of minimally 25 patients is needed. As different surgical procedures, both major and minor, may be performed on included patients and the baseline FVIII:C level may have a wide range (approximately 0.01-0.60 IU/mL), the included patient population may be heterogeneous. In addition, patients may drop-out during the perioperative treatment. This may for example be the case if patients are no longer eligible to receive desmopressin due to changed clinical status. However, no specific stopping criteria are in state. To overcome both the heterogeneity and possible drop-out, we aim to include 50 patients.

To reach our target sample size an ensure we do not miss any patients, all participating centers will be updated regularly by e-mail, newsletters and during meetings.

For peer review only

Outcome measures

Primary outcome:

The proportion of patients with FVIII trough levels within the FVIII target range during the first 72 hours after start of combination treatment. If a patient has one trough level outside the target range or needs off-protocol FVIII concentrate within the first 72 hours of combination the treatment, the primary endpoint is not reached in that patient. Off-protocol FVIII concentrate is defined as all administered FVIII concentrate outside the PK-guided dosing advice as given by the clinical pharmacologist. FVIII:C will be targeted according to the Dutch Treatment Guideline (Table 3).[23] The treating physician may deviate from the guideline and set different target ranges, based on bleeding phenotype/history or type of surgery.

FVIII:C measurements will be performed with the one-stage assay. Each participating may use its own assay. However, all centers are certified and accredited.

Secondary outcomes:

- FVIII concentrate consumption, expressed as the total amount of administered units of FVIII concentrate per kilogram per patient;
- Number and nature of adverse events during combined treatment;
- Incidence and severity of bleeding, where the severity of bleeding will be graded according to the ISTH criteria for major and minor bleeding;[24 25]
- Incidence of FVIII neutralizing antibodies; measured with the Bethesda assay
- Incidence of thrombosis, where thrombosis will be defined according to the Dutch guidelines on thrombosis, myocardial infarctions and strokes;[26 27]
- Incidence and extent of tachyphylaxis, defined as a reduction in the absolute increase in FVIII:C after the second and third desmopressin infusion;
- Medical costs and an economic evaluation;
- Experience quality of patient care, measured by a questionnaire

Data analysis plan

All baseline characteristics will be described as means and standard deviations or medians with interquartile ranges, dependent on whether the parameter is normally distributed. The primary outcome, the proportion of patients within FVIII target ranges, will be analysed by a chi-square test. Retrospective data will be used as a reference, as the DAVID-study only has one study arm.[3] Pharmacokinetic data will be analysed using the NONMEM® software package. Tachyphylaxis of FVIII:C response to desmopressin will be analysed with a paired t-test. Other

For peer review only

secondary outcomes will be documented in a descriptive manner, except for the economic analysis.

Economic analysis

An economic evaluation will be performed from a health care perspective from the day of surgery to 90 days postoperatively. The cost-effectiveness of combined treatment will be assessed by calculating the incremental cost-effectiveness ratio (ICER), defined as the difference in costs of combined treatment, compared to usual care, divided by the average change in effectiveness. Actual medical costs will be calculated by multiplying the volumes of healthcare use with the corresponding unit prices. Perioperative data resource use (desmopressin, FVIII concentrates, additional FVIII:C and VWF measurements, PK-profiling) will be collected from medical files. Usual medical costs (to compare to combination treatment costs) will be calculated based on a treatment protocol as it would have been without desmopressin use, taking into account patient's body weight, baseline FVIII:C and type of surgery. The reduction in costs will be represented by the usual medical costs (units of FVIII concentrates) minus the actual medical costs for combined treatment (units of FVIII concentrates, µg of desmopressin, extra FVIII:C measurements). An additional sensitivity analysis will be performed to assess the stability of the results to changes in costs and effectiveness parameters. Primary effectiveness parameter is the proportion of patients reaching FVIII target levels with desmopressin and FVIII concentrate combination treatment. Secondary effectiveness parameter is the frequency of adverse events during combination treatment.

Patient and Public Involvement

During the development of this study we worked closely together with the Netherlands Haemophilia Patient Society (NVHP). One representative of the NVHP is member of the project committee and helped us with the study design. Moreover, 5 members of the NVHP were invited to comment on the patient information. The design and information were adjusted according to their opinions and questions. The final results of the DAVID study will be communicated trough scientific international journals and at international conferences. One major conference may be that of the World Federation of Haemophilia, attended by both physicians, researchers and patients from all over the world. In addition, the results will be communicated in the magazine of the NVHP. Finally, results will also be implemented in the treatment guidelines and patient information will be adjusted accordingly.

For peer review only - http://bmjopen.bmj.com/site/about/guidelines.xhtml

Proof of concept

As a proof of concept, we present one case of a patient that was treated with desmopressin and FVIII concentrate combination treatment for the duration of 24 hours. The patient was a 53 year old male with moderate severe haemophilia A (FVIII:C 0.04 IU/ml). His body weight was 100 kg. He was in need of dental surgery and needed treatment to prevent bleeding. He received an infusion of desmopressin (0.3 µg/kg) over 30 minutes, followed 30 minutes later by FVIII concentrate in a dose of 25 IU/kg (2500 IU). He received a second dose of FVIII concentrate (20 IU/kg; i.e. 2000 IU) in the evening to maintain his FVIII:C above target values. Both FVIII concentrate dosages were determined using the integrated PK model and based on the patient's response to a previous desmopressin dose and a previous FVIII concentrate administration. Figure 2 shows the measured FVIII:C and the FVIII:C as predicted by the integrated PK model. The measured FVIII:C trough level and peak levels were within 10% of the predicted levels (Figure 2). The patient only had mild side effects of desmopressin, i.e. flushing and mild tachycardia (101 bpm). To prevent bleeding, he was also treated with tranexamic acid for multiple days. No bleeding was reported before and after the surgery. However, because of a possible infection of the wound he was treated with antibiotics.

If this patient would not have received combination treatment, he would have been treated with FVIII concentrate monotherapy both before and after the dental surgery. As his baseline FVIII:C was 0.04 IU/mL, his body weight 100 kg and the target FVIII peak level was 0.80-1.00 IU/mL, the FVIII loading dose would be (1.00-0.04)/0.02*100=4750 IU (rounded to entire vials). In the evening the patient would have received2500 IU to maintain the FVIII:C between the target FVIII. Therefore, combination treatment hypothetically saved 2750 IU FVIII concentrate in this patient.

Ethics and dissemination

The study was approved by the Medical Ethics Committee of the Erasmus University Medical Centre Rotterdam, the Netherlands. The study will be conducted according to GCP guidelines and the Declaration of Helsinki. Written informed consent will be obtained from all patients by a member of the research team. Results of the study will be communicated to the (inter)national medical and scientific community trough publication in high-ranking peer reviewed international journals and at (inter)national medical scientific conferences. Results of the study will also be implemented in the Dutch Haemophilia Treatment Guidelines. Hopefully the international society of Haemophilia Treatment Centres will adapt the results in their guidelines as well.

For peer review only

Data monitoring committee and serious adverse events

This study does not carry any large safety risks as both FVIII concentrates and desmopressin are registered therapeutics for haemophilia treatment. In addition, to guarantee safety for all patients in this study, FVIII:C levels will be closely monitored to prevent any additional bleeding risks. Therefore a data safety monitoring board is not needed.

Serious adverse events (SAE) will be communicated to the sponsor within 24 hours. The sponsor will register the SAE within 15 days on *ToetsingOnline*, the Dutch registration system for SAEs.

Registration

The trial is registered in the Dutch Trial Registry, number NTR5383 (www.trialregister.nl) and in EudraCT: 2014-00535-14.

Discussion

This prospective trial will allow us to evaluate the safety and efficacy of combination treatment of desmopressin and FVIII concentrate in reaching target FVIII:C during bleeding episodes and in the perioperative setting. Dosing of FVIII concentrate will be determined by an integrated population PK model developed specifically for non-severe haemophilia A patients to be treated with combination treatment. Fifty non-severe haemophilia A patients will be included from all haemophilia treatment centres in the Netherlands to reach our aim.

The DAVID study has some limitations. First, this study is not designed as a randomized controlled trial; it does not include a control group. Therefore, no direct comparison to standard treatment will be possible. However, we performed an extensive retrospective cohort study to determine the effectiveness of current clinical practice in which 37 patients undergoing 52 surgeries, were evaluated.[3] Moreover, the amount of FVIII concentrate that would have been administered to the patients included in the DAVID study as if desmopressin was not administered and as if no PK-guided dosing was applied, will be calculated.

The second limitation is the heterogeneity of the study population. All types of surgery, unless not compatible with desmopressin treatment, may be included in the study. To limit the effects of this heterogeneity, only patients with an expected treatment duration of at least 48 hours will be included. Moreover, the data that were used to develop the integrated population PK model also included data from various types of surgery.

For peer review only

Conclusion

In the DAVID study, efficacy and safety of desmopressin and FVIII concentrate combination treatment in non-severe haemophilia A patients will be determined. Using this innovative approach treatment of non-severe haemophilia A patients both during bleeding episodes and in the perioperative setting may be improved.

Authors' contribution

MK, MCn, FL and RM designed the study and critically revised the manuscript. LS, SP and RH wrote the manuscript and refined the study design. MD, KF, EB, MCo, JE, BL-G, KM, LN and EM critically revised the manuscript and refined the study design.

We would like to specially thank MD for her contribution on behalf of the NVHP and the five NVHP members who helped to improve the patient information. In addition we would like to thank the pilot patient for his cooperation and for letting us use his case as an example.

Funding statement

This work was supported by ZonMw, (grant number 836031003), the Dutch organisation for Health Research and Development.

Competing interest statement

L.M. Schütte; received reimbursement from CSL-Behring for attending a symposium, not related to this study.

M.H. Cnossen; received unrestricted research/educational funding for various projects as well as travel fees from the following institutions and companies: ZonMW, Innovatiefonds, Pfizer, Baxalta/Shire, Bayer Schering Pharma, Novo Nordisk, Novartis, Roche and CSL Behring, all not related to this study.

R.M. van Hest, E.A.M. Beckers, M. Coppens, M. Driessens, L. Nieuwenhuizen, S.Polinder: nothing to disclose relevant to the DAVID study.

K. Fijnvandraat: is a member of the European Hemophilia Treatment and Standardization Board sponsored by Baxter, has received unrestricted research grants from CSL Behring and Bayer, and has given lectures at educational symposiums organized by Pfizer, Bayer and Baxter.

J. Eikenboom: received research funding from CSL Behring and honorarium for educational activity from Roche, not related to this study.

B.A.P. Laros-van Gorkom: received unrestricted educational grants from Baxter and CSL Behring, and speaker fees from Sanquin.

For peer review only

K. Meijer: research support from Bayer, Sanquin and Pfizer; speaker fees from Bayer, Sanquin, Boehringer Ingelheim, BMS and Aspen; consulting fees from Uniqure, not related to this study

F.W.G. Leebeek; received unrestricted research grants from CSL-Behring and Baxalta/Shire not related to this study. He is consulant for Shire, NovoNordisk and UniQure. Fees go to the university.

R.A.A. Mathôt: received personal fees from Merck Sharp & Dohme and Zeria and grants from Bayer, UCB Pharma and Hoffman La Roche with no involvement in this study.

M.J.H.A.Kruip; received unrestricted research grants from Pfizer, Innovatiefonds and Ferring with no involvement in this study.

References

1. Hazendonk HC, Lock J, Mathot RA, et al. Under dosing and overdosing of factor VIII in the perioperative setting in haemophilia a: A need for optimization of dosing. Haemophilia 2015;**21**((Hazendonk H.C.; Lock J.; Bouzariouh H.; Cnossen M.H.) Department of Pediatric Hematology, Erasmus University Medical Centre, Sophia Childrens Hospital, Rotterdam, Netherlands):81-82.
2. Hazendonk HC, Lock J, Mathot RA, et al. Perioperative treatment of hemophilia A patients: blood group O patients are at risk of bleeding complications. J Thromb Haemost 2016;**14**(3):468-78.
3. Schütte LM, de Rooij N, Hazendonk HCAM, et al. Perioperative FVIII Concentrate Treatment in Mild Hemophilia a Patients Shows a High Rate of Overdosing - David/Opti-Clot Studies. Blood 2015;**126**(23):3510-10.
4. Ritchie B, Woodman RC, Poon MC. Deep venous thrombosis in hemophilia A. Am J Med 1992;**93**(6):699-700.
5. Escuriola Ettingshausen C, Martinez Saguer I, Kreuz W. Portal vein thrombosis in a patient with severe haemophilia A and F V G1691A mutation during continuous infusion of F VIII after intramural jejunal bleeding - Successful thrombolysis under heparin therapy. European Journal of Pediatrics, Supplement 1999;**158**(3):S180-S82.
6. Russell Z, Riconda D, Pollack L, et al. Thrombosis in a pregnant hemophilia A carrier after intrapartum recombinant factor VIII. Obstet Gynecol 2005;**105**(4):875-6.
7. Eckhardt CL, Mauser-Bunschoten EP, Peters M, et al. Inhibitor incidence after intensive FVIII replacement for surgery in mild and moderate haemophilia A: a prospective national study in the Netherlands. Br J Haematol 2012;**157**(6):747-52.
8. Astermark J. FVIII inhibitors: pathogenesis and avoidance. Blood 2015;**125**(13):2045-51.
9. d'Oiron R, Pipe SW, Jacquemin M. Mild/moderate haemophilia A: new insights into molecular mechanisms and inhibitor development. Haemophilia 2008;**14 Suppl 3**:138-46.
10. Kaufmann JE, Vischer UM. Cellular mechanisms of the hemostatic effects of desmopressin (DDAVP). J Thromb Haemost 2003;**1**(4):682-9.
11. Mannucci. Desmopressin (DDAVP) in the treatment of bleeding disorders. World federation of hemophilia 2012.
12. Pan J, Dinh TT, Rajaraman A, et al. Patterns of expression of factor VIII and von Willebrand factor by endothelial cell subsets in vivo. Blood 2016.
13. Stoof SC, Sanders YV, Petrij F, et al. Response to desmopressin is strongly dependent on F8 gene mutation type in mild and moderate haemophilia A. Thromb Haemost 2013;**109**(3):440-9.
14. Nance D, Fletcher SN, Bolgiano DC, et al. Factor VIII mutation and desmopressin-responsiveness in 62 patients with mild haemophilia A. Haemophilia 2013;**19**(5):720-6.

For peer review only

15. Castaman G, Mancuso ME, Giacomelli SH, et al. Molecular and phenotypic determinants of the response to desmopressin in adult patients with mild hemophilia A. J Thromb Haemost 2009;**7**(11):1824-31.
16. Stoof SC, Cnossen MH, de Maat MP, et al. Side effects of desmopressin in patients with bleeding disorders. Haemophilia 2016;**22**(1):39-45.
17. Mannucci PM, Bettega D, Cattaneo M. Patterns of development of tachyphylaxis in patients with haemophilia and von Willebrand disease after repeated doses of desmopressin (DDAVP). Br J Haematol 1992;**82**(1):87-93.
18. Björkman S. Limited blood sampling for pharmacokinetic dose tailoring of FVIII in the prophylactic treatment of haemophilia A. Haemophilia 2010;**16**(4):597-605.
19. Carlsson M, Berntorp E, Bjorkman S, et al. Improved cost-effectiveness by pharmacokinetic dosing of factor VIII in prophylactic treatment of haemophilia A. Haemophilia 1997;**3**(2):96-101.
20. Hazendonk H, Fijnvandraat K, Lock J, et al. A population pharmacokinetic model for perioperative dosing of factor VIII in hemophilia A patients. Haematologica 2016.
21. Hazendonk HC, van Moort I, Fijnvandraat K, et al. The "OPTI-CLOT" trial. A randomised controlled trial on periOperative PharmacokineTIc-guided dosing of CLOTting factor concentrate in haemophilia A. Thromb Haemost 2015;**114**(3):639-44.
22. Schutte LM, van Hest RM, Stoof SCM, et al. Pharmacokinetic Modelling to Predict FVIII:C Response to Desmopressin and Its Reproducibility in Nonsevere Haemophilia A Patients. Thromb Haemost 2018.
23. Leebeek FWG, Mauser-Bunschoten EP. *Diagnostiek en behandeling van hemofilie en aanverwante hemostasestoornissen : richtlijn*. Alphen aan den Rijn: Van Zuiden Communications, 2009.
24. Kaatz S, Ahmad D, Spyropoulos AC, et al. Definition of clinically relevant non-major bleeding in studies of anticoagulants in atrial fibrillation and venous thromboembolic disease in non-surgical patients: communication from the SSC of the ISTH. J Thromb Haemost 2015;**13**(11):2119-26.
25. Schulman S, Kearon C, Subcommittee on Control of Anticoagulation of the S, et al. Definition of major bleeding in clinical investigations of antihemostatic medicinal products in non-surgical patients. J Thromb Haemost 2005;**3**(4):692-4.
26. Kwaliteitsinstituut voor de Gezondheidszorg, C. B. O. *Diagnostiek, preventie en behandeling van veneuze trombo-embolie en secundaire preventie van arteriële trombose*. Alphen aan den Rijn: Van Zuiden Communications, 2008.
27. Kwaliteitsinstituut voor de Gezondheidszorg CBO. *Diagnostiek, behandeling en zorg voor patienten met een beroerte : richtlijn*. Utrecht: Nederlandse Vereniging voor Neurologie; Utrecht: Kwaliteitsinstituut voor de Gezondheidszorg CBO, 2008.

For peer review only

Legends to tables and figures

Table 1: Model characteristics of the population PK model for FVIII concentrates

The covariate associations between VWF:Ag on clearance was modeled as a nonlinear function, while the association between most recent FVIII:C and baseline FVIII:C was modeled as a linear function. The numbers for the covariate associations (-0.285 and 0.891) describe both the shape and magnitude of the covariate effect: the more the number deviates from 0 the larger the effect of a covariate on the PK parameter. RSE = relative standard error; VWF:Ag = von Willebrand factor antigen; IIV = Inter-individual variability

Table 2: Overview of blood sampling for FVIII:C measurements

Grey: obligatory measurements

† only in case of elective surgery, otherwise before first desmopressin administration

‡ pre = before desmopressin, post = after desmopressin, peak = after FVIII-concentrate, post-treat = after surgery; in case of bleeding; 2-6 hours after desmopressin, trough = before next dose of FVIII-concentrate

‡ pre = before desmopressin, post = after desmopressin, peak = after loading dose FVIII-concentrate, post-treat = after surgery; in case of bleeding: 2-6 hours after desmopressin, pre-adjust = before dosage , steady state = FVIII-measurement at random time point

FVIII measurements are shown here until day 3 after start of combination treatment. Monitoring will continue if treatment is still necessary.

Table 3: Target ranges for FVIII:C in IU/ml in the perioperative setting[23]

Figure 1: Flowchart of study

Figure 2: FVIII:C course after desmopressin and FVIII concentrate combination treatment in a pilot patient with moderate haemophilia A (FVIII:C 0.04 IU/ml). T_0 = preoperative desmopressin infusion. Lines are the predicted FVIII:C. Predictions were based on a previous desmopressin test dose and FVIII concentrate administration, prior to study inclusion. Solid line is total FVIII:C and can be measured. Open circles are measured FVIII:C. Infusions of desmopressin and FVIII concentrate (FVIII con.) are depicted with arrows.

For peer review only

Table 1: Model characteristics of the population PK model for FVIII concentrates

Parameter	Population estimate	RSE (%)
Baseline FVIII:C (IU/mL)	0.211	10.9
Clearance (mL/h)	208	10
Volume of distribution (mL)	3400	4.9
Proportional error (%)	17.3	8.4
VWF:Ag on clearance	-0.285	15.8
Most recent FVIII:C on baseline FVIII:C	0.891	5.4
IIV of baseline FVIII:C (%)	54.7	11.0
IIV of clearance (%)	37.4	19.7
IIV of volume of distribution (%)	20.8	26.8

The covariate associations between VWF:Ag on clearance was modeled as a nonlinear function, while the association between most recent FVIII:C and baseline FVIII:C was modeled as a linear function. The numbers for the covariate associations (-0.285 and 0.891) describe both the shape and magnitude of the covariate effect: the more the number deviates from 0 the larger the effect of a covariate on the PK parameter. RSE = relative standard error; VWF:Ag = von Willebrand factor antigen; IIV = Inter-individual variability

For peer review only

Table 2: Overview of blood sampling for FVIII:C measurements

	Within 4 weeks before surgery†	D0 = Day of first desmopressin infusion				D1				D2		D3	**4-8 weeks after FVIII treatment**
Time – bolus infusions‡	baseline	pre	post	peak	post-treat	pre	post	peak	pre	post	peak	trough	
Time – continuous infusion‡	baseline	pre	post	peak	post-treat	pre	post	pre-adjust	pre	post	pre-adjust	steady state	
Primary endpoint						X			X			X	
Sodium	X	X				X			X			X	
Neutralizing antibodies	X												X

Grey: obligatory measurements

† only in case of elective surgery, otherwise before first desmopressin administration

‡ pre = before desmopressin, post = after desmopressin, peak = after FVIII-concentrate, post-treat = after surgery; in case of bleeding; 2-6 hours after desmopressin, trough = before next dose of FVIII-concentrate

‡ pre = before desmopressin, post = after desmopressin, peak = after loading dose FVIII-concentrate, post-treat = after surgery; in case of bleeding: 2-6 hours after desmopressin, pre-adjust = before dosage , steady state = FVIII-measurement at random time point

FVIII measurements are shown here until day 3 after start of combination treatment. Monitoring will continue if treatment is still necessary.

Table 3: Target ranges for FVIII:C in IU/ml in the perioperative setting[23]

Time	FVIII target level (IU/ml)
**Day 0 (hour 0-24)**	0.8-1.0 (peak)
**Day 1-4 (hour 24-96)**	0.5-0.8 (trough)
**Day ≥ 5 (hour > 96)**	0.3-0.5 (trough)

For peer review only - http://bmjopen.bmj.com/site/about/guidelines.xhtml

For peer review only

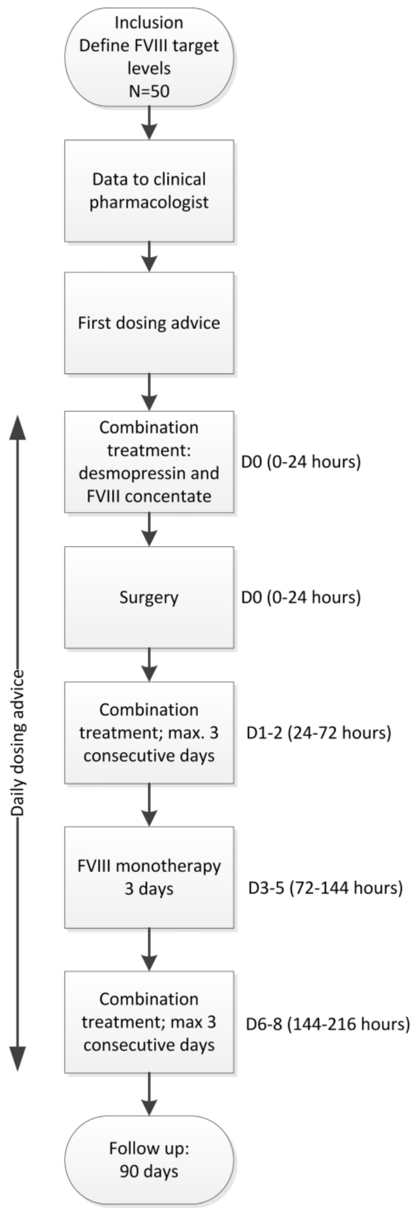

Figure 1: Flowchart of study

263x773mm (300 x 300 DPI)

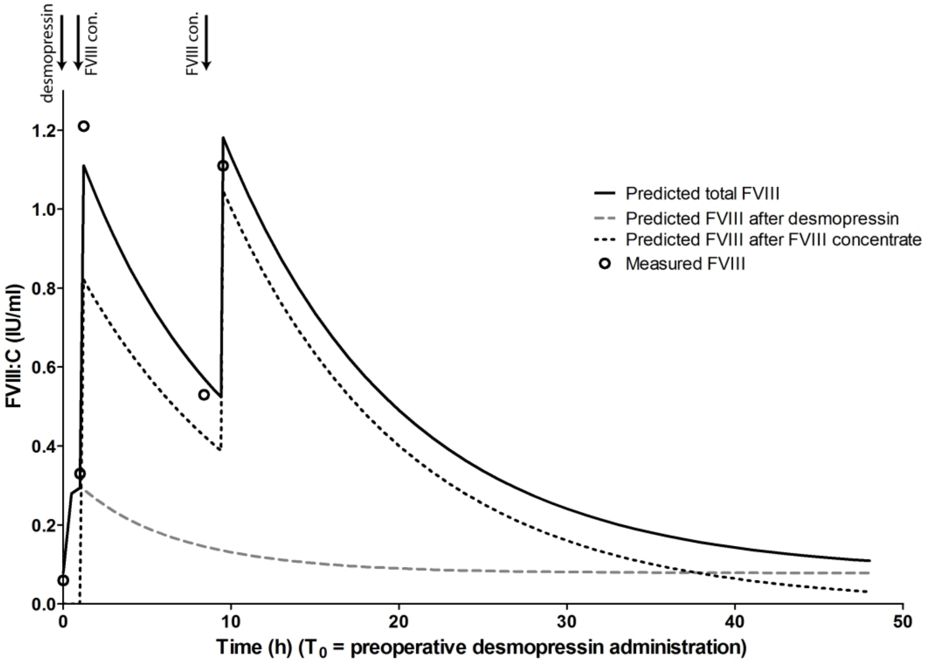

Figure 2: FVIII:C course after desmopressin and FVIII concentrate combination treatment in a pilot patient with moderate haemophilia A (FVIII:C 0.04 IU/ml). T0 = preoperative desmopressin infusion. Lines are the predicted FVIII:C. Predictions were based on a previous desmopressin test dose and FVIII concentrate administration, prior to study inclusion. Solid line is total FVIII:C and can be measured. Open circles are measured FVIII:C. Infusions of desmopressin and FVIII concentrate (FVIII con.) are depicted with arrows.

246x171mm (300 x 300 DPI)

SPIRIT
STANDARD PROTOCOL ITEMS: RECOMMENDATIONS FOR INTERVENTIONAL TRIALS

SPIRIT 2013 Checklist: Recommended items to address in a clinical trial protocol and related documents*

Section/item	Item No	Description	Addressed on page number
**Administrative information**			
Title	1	Descriptive title identifying the study design, population, interventions, and, if applicable, trial acronym	1_____________
Trial registration	2a	Trial identifier and registry name. If not yet registered, name of intended registry	2_& 13______
	2b	All items from the World Health Organization Trial Registration Data Set	Only in full protocol; available on request_
Protocol version	3	Date and version identifier	NA_________
Funding	4	Sources and types of financial, material, and other support	14_____________
Roles and responsibilities	5a	Names, affiliations, and roles of protocol contributors	_1 & 14
	5b	Name and contact information for the trial sponsor	1
	5c	Role of study sponsor and funders, if any, in study design; collection, management, analysis, and interpretation of data; writing of the report; and the decision to submit the report for publication, including whether they will have ultimate authority over any of these activities	14
	5d	Composition, roles, and responsibilities of the coordinating centre, steering committee, endpoint adjudication committee, data management team, and other individuals or groups overseeing the trial, if applicable (see Item 21a for data monitoring committee)	Only in full protocol; available on request___
**Introduction**			

For peer review only

Background and rationale	6a	Description of research question and justification for undertaking the trial, including summary of relevant studies (published and unpublished) examining benefits and harms for each intervention	4 & 5
	6b	Explanation for choice of comparators	NA
Objectives	7	Specific objectives or hypotheses	5
Trial design	8	Description of trial design including type of trial (eg, parallel group, crossover, factorial, single group), allocation ratio, and framework (eg, superiority, equivalence, noninferiority, exploratory)	6
**Methods: Participants, interventions, and outcomes**			
Study setting	9	Description of study settings (eg, community clinic, academic hospital) and list of countries where data will be collected. Reference to where list of study sites can be obtained	6
Eligibility criteria	10	Inclusion and exclusion criteria for participants. If applicable, eligibility criteria for study centres and individuals who will perform the interventions (eg, surgeons, psychotherapists)	6
Interventions	11a	Interventions for each group with sufficient detail to allow replication, including how and when they will be administered	7
	11b	Criteria for discontinuing or modifying allocated interventions for a given trial participant (eg, drug dose change in response to harms, participant request, or improving/worsening disease)	NA
	11c	Strategies to improve adherence to intervention protocols, and any procedures for monitoring adherence (eg, drug tablet return, laboratory tests)	7
	11d	Relevant concomitant care and interventions that are permitted or prohibited during the trial	7
Outcomes	12	Primary, secondary, and other outcomes, including the specific measurement variable (eg, systolic blood pressure), analysis metric (eg, change from baseline, final value, time to event), method of aggregation (eg, median, proportion), and time point for each outcome. Explanation of the clinical relevance of chosen efficacy and harm outcomes is strongly recommended	10
Participant timeline	13	Time schedule of enrolment, interventions (including any run-ins and washouts), assessments, and visits for participants. A schematic diagram is highly recommended (see Figure)	Table 2

Sample size	14	Estimated number of participants needed to achieve study objectives and how it was determined, including clinical and statistical assumptions supporting any sample size calculations	9
Recruitment	15	Strategies for achieving adequate participant enrolment to reach target sample size	9
**Methods: Assignment of interventions (for controlled trials)**			
Allocation:			
Sequence generation	16a	Method of generating the allocation sequence (eg, computer-generated random numbers), and list of any factors for stratification. To reduce predictability of a random sequence, details of any planned restriction (eg, blocking) should be provided in a separate document that is unavailable to those who enrol participants or assign interventions	NA
Allocation concealment mechanism	16b	Mechanism of implementing the allocation sequence (eg, central telephone; sequentially numbered, opaque, sealed envelopes), describing any steps to conceal the sequence until interventions are assigned	NA
Implementation	16c	Who will generate the allocation sequence, who will enrol participants, and who will assign participants to interventions	NA
Blinding (masking)	17a	Who will be blinded after assignment to interventions (eg, trial participants, care providers, outcome assessors, data analysts), and how	NA
	17b	If blinded, circumstances under which unblinding is permissible, and procedure for revealing a participant's allocated intervention during the trial	NA
**Methods: Data collection, management, and analysis**			
Data collection methods	18a	Plans for assessment and collection of outcome, baseline, and other trial data, including any related processes to promote data quality (eg, duplicate measurements, training of assessors) and a description of study instruments (eg, questionnaires, laboratory tests) along with their reliability and validity, if known. Reference to where data collection forms can be found, if not in the protocol	8-10
	18b	Plans to promote participant retention and complete follow-up, including list of any outcome data to be collected for participants who discontinue or deviate from intervention protocols	10-11

Data management	19	Plans for data entry, coding, security, and storage, including any related processes to promote data quality (eg, double data entry; range checks for data values). Reference to where details of data management procedures can be found, if not in the protocol	Only in full protocol; available on request
Statistical methods	20a	Statistical methods for analysing primary and secondary outcomes. Reference to where other details of the statistical analysis plan can be found, if not in the protocol	10
	20b	Methods for any additional analyses (eg, subgroup and adjusted analyses)	10+11
	20c	Definition of analysis population relating to protocol non-adherence (eg, as randomised analysis), and any statistical methods to handle missing data (eg, multiple imputation)	NA
**Methods: Monitoring**			
Data monitoring	21a	Composition of data monitoring committee (DMC); summary of its role and reporting structure; statement of whether it is independent from the sponsor and competing interests; and reference to where further details about its charter can be found, if not in the protocol. Alternatively, an explanation of why a DMC is not needed	13
	21b	Description of any interim analyses and stopping guidelines, including who will have access to these interim results and make the final decision to terminate the trial	NA
Harms	22	Plans for collecting, assessing, reporting, and managing solicited and spontaneously reported adverse events and other unintended effects of trial interventions or trial conduct	13 + in full protocol; available on request
Auditing	23	Frequency and procedures for auditing trial conduct, if any, and whether the process will be independent from investigators and the sponsor	NA
**Ethics and dissemination**			
Research ethics approval	24	Plans for seeking research ethics committee/institutional review board (REC/IRB) approval	12
Protocol amendments	25	Plans for communicating important protocol modifications (eg, changes to eligibility criteria, outcomes, analyses) to relevant parties (eg, investigators, REC/IRBs, trial participants, trial registries, journals, regulators)	Only in full protocol; available on request

Consent or assent	26a	Who will obtain informed consent or assent from potential trial participants or authorised surrogates, and how (see Item 32)	12
	26b	Additional consent provisions for collection and use of participant data and biological specimens in ancillary studies, if applicable	Only in full protocol; available on request
Confidentiality	27	How personal information about potential and enrolled participants will be collected, shared, and maintained in order to protect confidentiality before, during, and after the trial	Only in full protocol; available on request
Declaration of interests	28	Financial and other competing interests for principal investigators for the overall trial and each study site	14+15
Access to data	29	Statement of who will have access to the final trial dataset, and disclosure of contractual agreements that limit such access for investigators	Only in full protocol; available on request
Ancillary and post-trial care	30	Provisions, if any, for ancillary and post-trial care, and for compensation to those who suffer harm from trial participation	Only in full protocol; available on request
Dissemination policy	31a	Plans for investigators and sponsor to communicate trial results to participants, healthcare professionals, the public, and other relevant groups (eg, via publication, reporting in results databases, or other data sharing arrangements), including any publication restrictions	12
	31b	Authorship eligibility guidelines and any intended use of professional writers	NA
	31c	Plans, if any, for granting public access to the full protocol, participant-level dataset, and statistical code	NA
**Appendices**			
Informed consent materials	32	Model consent form and other related documentation given to participants and authorised surrogates	Only in full protocol; available on request

For peer review only

For peer review only - http://bmjopen.bmj.com/site/about/guidelines.xhtml

Biological specimens	33	Plans for collection, laboratory evaluation, and storage of biological specimens for genetic or molecular analysis in the current trial and for future use in ancillary studies, if applicable	Only in full protocol; available on request

*It is strongly recommended that this checklist be read in conjunction with the SPIRIT 2013 Explanation & Elaboration for important clarification on the items. Amendments to the protocol should be tracked and dated. The SPIRIT checklist is copyrighted by the SPIRIT Group under the Creative Commons "Attribution-NonCommercial-NoDerivs 3.0 Unported" license.

For peer review only

BMJ Open

Desmopressin treatment combined with clotting factor VIII concentrates in patients with non-severe haemophilia A: protocol for a multicentre single-armed trial, the DAVID study

Journal:	*BMJ Open*
Manuscript ID	bmjopen-2018-022719.R2
Article Type:	Protocol
Date Submitted by the Author:	08-Oct-2018
Complete List of Authors:	Schütte, Lisette; Erasmus MC, Haematology<br>Cnossen, Marjon; Erasmus MC Sophia, Pediatric Hematology<br>Van Hest, Reinier; Amsterdam UMC, Department of Hospital Pharmacy – Clinical Pharmacology Unit<br>Driessens, Mariette; Netherlands Haemophilia Patient Society<br>Fijnvandraat, Karin; Amsterdam UMC, Emma Children's hospital, Pediatric Haematology; Sanquin Research, Plasma Proteins<br>Polinder, Suzanne; Erasmus MC, Public Health<br>Beckers, Erik; Maastricht University Medical Centre, Haematology<br>Coppens, Michiel; Amsterdam UMC, Vascular Medicine<br>Eikenboom, Jeroen; Leids Universitair Medisch Centrum, Thrombosis and Haemostasis<br>Laros-van Gorkom, Britta; Radboudumc, Haematology<br>Meijer, Karina; Universitair Medisch Centrum Groningen, Department of Haematology<br>Nieuwenhuizen, Laurens; Maxima Medisch Centrum, Haematology<br>Mauser-Bunschoten, Evelien; University Medical Centre Utrecht, Van Creveldclinic<br>Leebeek, Frank; Erasmus MC, Hematology<br>Mathôt, Ron; Amsterdam UMC, Department of Hospital Pharmacy – Clinical Pharmacology Unit<br>Kruip, Marieke; Erasmus MC, Hematology
**Primary Subject Heading**:	Haematology (incl blood transfusion)
Secondary Subject Heading:	Patient-centred medicine, Pharmacology and therapeutics, Surgery
Keywords:	Haemophilia A, Desmopressin, FVIII concentrate, SURGERY, Pharmacokinetic modelling

SCHOLARONE™
Manuscripts

Desmopressin treatment combined with clotting factor VIII concentrates in patients with non-severe haemophilia A: protocol for a multicentre single-armed trial, the DAVID study

L.M. Schütte[1], M.H. Cnossen[2], R.M. van Hest[3], M.H.E. Driessens[4], K. Fijnvandraat[5], S. Polinder[6], E.A.M. Beckers[7], M. Coppens[8], J. Eikenboom[9], B.A.P. Laros-van Gorkom[10], K. Meijer[11], L. Nieuwenhuizen[12], E.P. Mauser-Bunschoten[13], F.W.G. Leebeek[1], R.A.A. Mathôt[3], M.J.H.A. Kruip[1]

Corresponding Author:
Dr. Marieke J.H.A. Kruip
Room Na-823
P.O. Box 2040
3000 CA Rotterdam
The Netherlands
e-mail: m.kruip@erasmusmc.nl
telephone number: +31 10 7033123

Affiliations
[1]Department of Haematology, Erasmus University Medical Centre, Rotterdam, the Netherlands; [2]Department of Paediatric Haematology, Erasmus University Medical Centre - Sophia Children's Hospital, Rotterdam, the Netherlands; [3]Department of Hospital Pharmacy – Clinical Pharmacology Unit, Amsterdam UMC, University of Amsterdam, the Netherlands; [4]Netherlands Haemophilia Patient Society, Nijkerk, the Netherlands; [5]Department of Paediatric Haematology, Amsterdam UMC, University of Amsterdam , The Netherlands; Department of Plasma Proteins, Sanquin Research, Amsterdam, The Netherlands; [6]Department of Public Health, Erasmus University Medical Centre, Rotterdam, the Netherlands; [7]Department of Haematology, Maastricht University Medical Centre, Maastricht, the Netherlands; [8]Department of Vascular Medicine, Amsterdam UMC, University of Amsterdam, the Netherlands; [9]Department of Thrombosis and Haemostasis, Leiden University Medical Centre, Leiden, the Netherlands; [10]Department of Haematology, Radboud University Medical Centre, Nijmegen, the Netherlands; [11]Department of Haematology, University Medical Centre Groningen, Groningen, the Netherlands; [12]Department of Haematology, Maxima Medical Centre, Eindhoven, the Netherlands; [13]Van Creveldkliniek, Utrecht, the Netherlands

For peer review only

For peer review only - http://bmjopen.bmj.com/site/about/guidelines.xhtml

Word count: 3793

Abstract

Introduction:

Haemophilia A is an inherited bleeding disorder characterised by factor VIII (FVIII) deficiency. In non-severe haemophilia A patients, surgery and bleeding are the main indications for treatment with FVIII concentrate. A recent study reported that standard dosing frequently results in FVIII levels (FVIII:C) below or above FVIII target ranges, leading to respectively a bleeding risk or excessive costs. In addition, FVIII concentrate treatment carries a risk of development of neutralizing antibodies.

An alternative is desmopressin, which releases endogenous FVIII and von Willebrand factor. In most non-severe haemophilia A patients, desmopressin alone is not enough to achieve FVIII target levels during surgery or bleeding. We hypothesize that combined pharmacokinetic (PK) guided administration of desmopressin and FVIII concentrate may improve dosing accuracy and reduces FVIII concentrate consumption.

Methods and analysis:

In the DAVID study, fifty non-severe haemophilia A patients (FVIII:C ≥0.01 IU/ml) with a bleeding episode or undergoing surgery will receive desmopressin and FVIII concentrate combination treatment. The necessary dose of FVIII concentrate to reach FVIII target levels after desmopressin administration, will be calculated with a population PK model. The primary endpoint is the proportion of patients reaching FVIII target levels during the first 72 hours after start of the combination treatment. This approach was successfully tested in one pilot patient who received perioperative combination treatment.

Ethics and dissemination:

The DAVID study was approved by the medical ethics committee of the Erasmus MC. Results of the study will be communicated trough publication in international scientific journals and presentation at (inter)national conferences.

Trial registration: NTR5383 (www.trialregister.nl), EudraCT: 2014-00535-14

For peer review only - http://bmjopen.bmj.com/site/about/guidelines.xhtml

Strengths and limitations of this study

- The DAVID-study is a multicentre, prospective trial including patients from eight Haemophilia Treatment Centres in the Netherlands.
- Desmopressin and FVIII concentrate combination treatment is an innovative treatment option for non-severe haemophilia A patients.
- By using maximum *a posteriori* Bayesian estimation based on an integrated population pharmacokinetic model and measured FVIII levels, the dosing of FVIII concentrate may be improved, with less FVIII levels above and below target.
- The DAVID study is a single-armed trial.
- The study population is heterogeneous as all types of surgery and bleeding episodes may be included.

For peer review only

Introduction

Haemophilia A is an X-linked bleeding disorder characterized by a factor VIII (FVIII) deficiency. Non-severe haemophilia A patients (FVIII:C ≥ 0.01 IU/ml) suffer from bleeding in the perioperative setting and after (minor) trauma. Treatment consists of either desmopressin or FVIII concentrate.

FVIII concentrate is an effective, but expensive treatment option. With current dosing based on body weight, baseline FVIII:C and target FVIII:C, FVIII:C both below (7-45%) and above (32-81%) FVIII target levels have been observed.[1 2] FVIII:C below target may lead to an increased bleeding risk, whereas levels above target increase the costs. Excessively high FVIII:C may also be associated with thrombosis.[3-5] In addition, high FVIII concentrate doses may induce the development of FVIII neutralizing antibodies, causing ineffectiveness of treatment with FVIII concentrate. In cases where antibodies cross-react with patient's endogenous FVIII they can even cause a severe phenotype with spontaneous bleeding.[6-8] Therefore, dosing within the target range with restriction of FVIII concentrate use is important.

An alternative to FVIII concentrate is desmopressin, a synthetic analogue of vasopressin. It releases von Willebrand factor (VWF) from the endothelium and FVIII, thereby improving haemostasis.[9-11] Although treatment is often effective, FVIII:C response to desmopressin exhibits a high variability between haemophilia A patients. Contributing factors reported in literature are age, baseline FVIII:C and the *F8*-gene mutation.[12-14] However, these factors do not explain the observed variability entirely. In addition, in most patients FVIII:C does not increase sufficiently to prevent perioperative bleeding.

Other limitations of desmopressin are the experienced side effects and tachyphylaxis. Most side effects are mild and transient, such as vasodilation. More severe side-effects like hyponatremia are rare and can usually be prevented by a fluid restriction.[15] Tachyphylaxis occurs when repeated dosages of desmopressin are given with short time intervals (12-24 hours). The decrease in FVIII:C response is approximately 30% from the second dose onwards in case of a 24 hour interval and is believed to be caused by a temporary depletion of VWF and FVIII from the endothelium.[16]

Combined administration of desmopressin and FVIII concentrate may be able to overcome several of the drawbacks of both separate treatment options. However, there is a lack of experience and knowledge with regard to the efficacy and safety of combination treatment. Moreover, optimization of dosing is necessary to get and keep FVIII:C within the target range.

A valuable approach could be pharmacokinetic (PK) guided dosing, where FVIII:C responses of future dosages are predicted based on a population pharmacokinetic model in

For peer review only - http://bmjopen.bmj.com/site/about/guidelines.xhtml

combination with a limited number (2-3) of FVIII:C measurements, obtained after administration of desmopressin and/or FVIII concentrate. This technique was previously shown to be effective in both the prophylactic and perioperative setting in severe and moderate haemophilia A patients.[17 18{Hazendonk, 2016 #546]. A prospective trial using PK guided dosing in the perioperative treatment of moderate and severe haemophilia A patients is ongoing.[19]

Therefore, we hypothesize that combined PK guided dosing of desmopressin and FVIII concentrate may be a feasible treatment option in non-severe haemophilia A patients with a bleeding episode or undergoing surgery. Our aim is to show that the proportion of patients with FVIII:C within the FVIII target ranges can be increased by the use of the combination treatment and PK-guided dosing. In addition, the consumption of FVIII concentrate will be reduced. This innovative approach in haemophilia treatment may be a promising alternative with an increase in quality of care and a concomitant cost reduction.

Methods and analysis

Objectives

The primary objective of the DAVID study is to evaluate if combination treatment of desmopressin and PK-guided dosing of FVIII concentrate is able to increase the proportion of non-severe haemophilia A patients with FVIII:C within the target range during the first 72 hours after start of combination treatment compared to historical controls.

Secondary objectives:

1. To assess FVIII concentrate consumption in all patients.
2. To acquire data to improve the integrated population PK model for desmopressin and FVIII concentrate combination treatment
3. To establish (possible) adverse events of combination treatment; e.g. side effects of desmopressin, bleeding episodes, development of neutralizing antibodies, and thrombotic events.
4. To evaluate the extent of tachyphylaxis after desmopressin treatment.
5. To perform an economical evaluation to quantify the potential cost reduction of the combination treatment.
6. To evaluate the experienced quality of patient care.

For peer review only - http://bmjopen.bmj.com/site/about/guidelines.xhtml

Design

The DAVID study is a multicentre single-arm trial in non-severe haemophilia A patients with a bleeding episode or in need of a surgical procedure. The study was designed by a project committee existing of members of all participating sites (see Supplementary data 1 and 2). Patients will receive combination treatment of desmopressin and FVIII concentrate instead of the conventional treatment which consists of FVIII concentrate monotherapy.

Patient selection and recruitment

Non-severe haemophilia A patients ≥ 12 years of age with a bleeding episode or undergoing a surgical procedure, requiring FVIII replacement therapy and having a sufficient response to desmopressin, will be included. Patients will be recruited from one of the following haemophilia treatment centres in the Netherlands: Erasmus University Medical Centre Rotterdam, Academic Medical Centre Amsterdam, Leiden University Medical Centre, University Medical Centre Groningen, Radboud university medical centre Nijmegen, University Medical Centre Utrecht, Maxima Medical Centre Eindhoven and Maastricht University Medical Centre (see also Supplementary data 2). They will be approached by telephone or during a visit to the (outpatient) clinic if they present with a bleeding or need a surgical procedure.

Inclusion criteria:

- Non-severe haemophilia A patients (FVIII:C ≥0.01 IU/ml)
- Requiring a surgical procedure or having a bleeding episode
- Requiring replacement therapy with FVIII concentrate for at least 48 hours
- Age between 12 and 70 years at study inclusion date
- (Parental) informed consent
- Results of a desmopressin test available (minimal absolute FVIII:C increase >0.2 IU/ml)

Exclusion criteria:

- Patients with other congenital or acquired haemostatic abnormalities
- Inadequate response to desmopressin (absolute increase in FVIII:C <0.2 IU/ml) during a previous desmopressin test
- FVIII neutralizing antibodies (in medical history), unless successfully treated with immunotolerance induction therapy
- Initiation of FVIII concentrate treatment >24 hours before study inclusion

For peer review only - http://bmjopen.bmj.com/site/about/guidelines.xhtml

- Patients not eligible for desmopressin treatment due to contraindications, e.g.: intolerance, interactions with co-medication or due to type of surgery

Interventions and study procedures

All patients will receive combination treatment of desmopressin and FVIII concentrate during a bleeding episode or in the perioperative setting for at least 48 hours. All patients will receive a standard dose of desmopressin intravenously (0.3 µg/kg; no maximum dose). In order to combine both medication regimens, an individualized dosing advice for FVIII concentrate will be provided by the clinical pharmacologist. The initial dosing advice will be based on the FVIII:C response observed after a test administration of desmopressin (see below) and previously collected patient and population pharmacokinetic data after administration of desmopressin (endogenous FVIII) and FVIII concentrate (exogenous FVIII). The treating physician states the duration of combination treatment, the mode of administration of FVIII concentrate (continuous or intermittent administration) and determines FVIII target ranges. During combination treatment FVIII:C will be assessed regularly. Accordingly dose adjustments for FVIII concentrate will be made iteratively based on the results of a desmopressin test administration and using Bayesian analysis. Bayesian analysis will be performed with the NONMEM® software using a dedicated integrated population model describing the PK of FVIII following both the administration of desmopressin and FVII concentrate. As bleeding or acute surgery calls for immediate treatment and constructing a dosing advice takes time, a patient may be included in the DAVID-study until 24 hours after the start of FVIII concentrate monotherapy.

Adherence to the study protocol will be improved by the use of a separate script per included patient, in which an approximated timeline, contact details and all responsibilities of the involved research and treatment team are written down.

Concomitant treatment

As patients receive desmopressin, they will have a fluid restriction of 1.5 L per 24 hours, till 24 hours after the last desmopressin administration. Furthermore, all concomitant treatment and therapeutics are allowed, except for the therapeutics specified in the exclusion criteria. This means other treatment than desmopressin or factor concentrate necessary to prevent or treat bleeding, such as tranexamic acid, is allowed as well. The same will apply for prophylactic treatment to prevent thrombosis. These treatment options will be applied at the discretion of the treating physician and will be documented for all patients.

For peer review only - http://bmjopen.bmj.com/site/about/guidelines.xhtml

Desmopressin test administration

All patients must have undergone a desmopressin test with at least three FVIII:C measurements. If performed during childhood (<18 years), the test is only admissible when performed ≤4 years before study inclusion. When no desmopressin test has been performed meeting these criteria, the test should be performed with a standard intravenous desmopressin dose of 0.3 µg/kg infused over 30 minutes with a minimum of three FVIII:C measurements: before the administration of desmopressin, around 1 hour after desmopressin administration for peak measurement and at least one sample thereafter, for example after 4 hours. All time points of blood sampling and end of desmopressin administration should be documented precisely, as well as exact desmopressin dose and duration of infusion.

Pharmacokinetic (PK) guided dosing

In order to provide an individualized dosing advice, a Bayesian analysis will be performed on the basis of an integrated population PK model. This population model has been constructed based on two population pharmacokinetic models: 1) a population PK model of FVIII:C response after desmopressin administration [20] and 2) a population PK model of FVIII:C after administration of FVIII concentrate.[20] This latter model was constructed based on perioperative data from 29 non-severe, adult haemophilia A patients from whom 245 FVIII:C measurements were available. The final model estimated the baseline FVIII:C to which estimated FVIII:C, that followed administration of FVIII concentrate, were added according to a one-compartment model with first-order elimination. Two covariates could be identified after multivariate regression analysis: VWF:Ag had a negative association with clearance and the most recently measured FVIII:C had a positive association with the estimated baseline. In table 1 the most important model characteristics are shown.

The integrated population PK model, used in this study, describes the average PK, including the variability of the baseline FVIII:C and FVIII:C response between and within patients following both the administration of desmopressin and the administration of FVIII concentrate. This model will be used for both the perioperative setting and around bleeding episodes. The individualized dosing advice for the first dose of FVIII concentrate will be provided based on this model along with the results of the desmopressin test administration and the perioperative FVIII target levels. Further dosing of FVIII concentrates will be adjusted daily by iterative maximum *a posteriori* Bayesian analysis based on the population PK model in conjunction with perioperatively measured FVIII:C. In this analysis the following information will be included: 1) FVIII release in response to desmopressin after the test dose 2) baseline FVIII:C and 3) measured FVIII:C (both

For peer review only - http://bmjopen.bmj.com/site/about/guidelines.xhtml

trough and peak levels)(figure 1 and table 2). Dose adjustments based on this iterative Bayesian analysis will be performed in NONMEM® software. All dosing advices will be given one day in advance.

Experienced quality of patient care

To assess experienced quality of care during this innovative intervention, two questionnaires will be given to the patients. Side effects will be assessed using the questionnaire previously developed and used by Stoof et al..[15] This questionnaire includes the occurrence of seven different self-reported side effects on a five-point scale and two on a ten-point scale. Side effects will be evaluated at two time points: before surgery and three days after surgery. In the second questionnaire (three days after surgery) experienced quality of care will be evaluated with the addition of three questions dedicated to desmopressin and FVIII concentrate combination treatment. Patients will report their satisfaction with the combination treatment on a scale of 1 to 100. They will have the opportunity to explain what is needed to improve the given grade. Finally, they can state their preference for one of the treatment options: FVIII concentrate monotherapy or combination treatment. The last question will be only asked to patients who have a previous experience with FVIII concentrate monotherapy.

<u>Sample size</u>

In the DAVID study, the proportion of patients that reach FVIII target levels with combination treatment in the first 72 hours postoperatively (without adding off-protocol FVIII concentrates) will be assessed. Historical data on current FVIII treatment show a proportion of 0.3 of non-severe haemophilia A patients with FVIII:C within target ranges in this time period.[2] A doubling of this proportion, leading to a proportion of patients of 0.6, is believed to be clinically relevant. To study this with a power of 90% and a two-sided significance level of 0.05, a sample size of minimally 25 patients is needed. As different surgical procedures, both major and minor, may be performed on included patients and the baseline FVIII:C level may have a wide range (approximately 0.01-0.60 IU/mL), the included patient population may be heterogeneous. In addition, patients may drop-out during the perioperative treatment. This may for example be the case if patients are no longer eligible to receive desmopressin due to changed clinical status. However, no specific stopping criteria are present. To overcome both the heterogeneity and possible drop-out, we aim to include 50 patients.

To reach our target sample size and ensure we do not miss any patients, all participating centers will be updated regularly by e-mail, newsletters and during meetings.

For peer review only - http://bmjopen.bmj.com/site/about/guidelines.xhtml

Outcome measures

Primary outcome:

The primary outcome will be the proportion of patients with FVIII trough levels within the FVIII target range during the first 72 hours after start of combination treatment. If a patient has one trough level outside the target range or needs off-protocol FVIII concentrate within the first 72 hours of combination the treatment, the primary endpoint is not reached in that patient. Off-protocol FVIII concentrate is defined as all administered FVIII concentrate outside the PK-guided dosing advice as given by the clinical pharmacologist. FVIII:C will be targeted according to the Dutch Treatment Guideline (Table 3).[21] The treating physician may deviate from the guideline and set different target ranges, based on bleeding phenotype/history or type of surgery.

FVIII:C measurements will be performed with the one-stage assay. Each participating may use its own assay. However, all centers are certified and accredited.

Secondary outcomes:

- FVIII concentrate consumption, expressed as the total amount of administered units of FVIII concentrate per kilogram per patient;
- Number and nature of adverse events during combined treatment;
- Incidence and severity of bleeding, where the severity of bleeding will be graded according to the ISTH criteria for major and minor bleeding;[22 23]
- Incidence of FVIII neutralizing antibodies; measured with the Bethesda assay
- Incidence of thrombosis, where thrombosis will be defined according to the Dutch guidelines on thrombosis, myocardial infarctions and strokes;[24 25]
- Incidence and extent of tachyphylaxis, defined as a reduction in the absolute increase in FVIII:C after the second and third desmopressin infusion;
- Medical costs and an economic evaluation;
- Experience quality of patient care, measured by a questionnaire

Data analysis plan

All baseline characteristics will be described as means and standard deviations or medians with interquartile ranges, dependent on whether the parameter is normally distributed. The primary outcome, the proportion of patients within FVIII target ranges, will be analysed by a chi-square test. Retrospective data will be used as a reference, as the DAVID-study only has one study arm.[2] Pharmacokinetic data will be analysed using the NONMEM® software package.

For peer review only - http://bmjopen.bmj.com/site/about/guidelines.xhtml

Tachyphylaxis of FVIII:C response to desmopressin will be analysed with a paired t-test. Other secondary outcomes will be documented in a descriptive manner, except for the economic analysis.

Economic analysis

An economic evaluation will be performed from a health care perspective from the day of surgery to 90 days postoperatively. The cost-effectiveness of combined treatment will be assessed by calculating the incremental cost-effectiveness ratio (ICER), defined as the difference in costs of combined treatment, compared to usual care, divided by the average change in effectiveness. Actual medical costs will be calculated by multiplying the volumes of healthcare use with the corresponding unit prices. Perioperative data resource use (desmopressin, FVIII concentrates, additional FVIII:C and VWF measurements, PK-profiling) will be collected from medical files. Usual medical costs (to compare to combination treatment costs) will be calculated based on a treatment protocol as it would have been without desmopressin use, taking into account patient's body weight, baseline FVIII:C and type of surgery. The reduction in costs will be represented by the usual medical costs (units of FVIII concentrates) minus the actual medical costs for combined treatment (units of FVIII concentrates, μg of desmopressin, extra FVIII:C measurements). An additional sensitivity analysis will be performed to assess the stability of the results to changes in costs and effectiveness parameters. The primary effectiveness parameter is the proportion of patients reaching FVIII target levels with desmopressin and FVIII concentrate combination treatment. The secondary effectiveness parameter is the frequency of adverse events during combination treatment.

Patient and Public Involvement

During the development of this study we worked closely together with the Netherlands Haemophilia Patient Society (NVHP). One representative of the NVHP is member of the project committee and helped us with the study design. Moreover, 5 members of the NVHP were invited to comment on the patient information. The design and information were adjusted according to their opinions and questions. The final results of the DAVID study will be communicated trough scientific international journals and at international conferences. One major conference may be that of the World Federation of Haemophilia, attended by both physicians, researchers and patients from all over the world. In addition, the results will be

For peer review only - http://bmjopen.bmj.com/site/about/guidelines.xhtml

communicated in the magazine of the NVHP. Finally, results will also be implemented in the treatment guidelines and patient information will be adjusted accordingly.

Proof of concept

As a proof of concept, we present one case of a patient that was treated with desmopressin and FVIII concentrate combination treatment for the duration of 24 hours. This patient has signed a BMJ consent form to publish his information in this manuscript. The patient was a 53 year old male with moderate severe haemophilia A (FVIII:C 0.04 IU/ml). His body weight was 100 kg. He was in need of dental surgery and needed treatment to prevent bleeding. He received an infusion of desmopressin (0.3 µg/kg) over 30 minutes, followed 30 minutes later by FVIII concentrate in a dose of 25 IU/kg (2500 IU). He received a second dose of FVIII concentrate (20 IU/kg; i.e. 2000 IU) in the evening to maintain his FVIII:C above target values. Both FVIII concentrate dosages were determined using the integrated PK model and based on the patient's response to a previous desmopressin dose and a previous FVIII concentrate administration. Figure 2 shows the measured FVIII:C and the FVIII:C as predicted by the integrated PK model. All measured FVIII:C levels were within 0.10 IU/ml of the predicted levels (Figure 2). The patient only had mild side effects of desmopressin, i.e. flushing and mild tachycardia (101 bpm). To prevent bleeding, he was also treated with tranexamic acid for multiple days. No bleeding was reported before and after the surgery. However, because of a possible infection of the wound he was treated with antibiotics.

If this patient would not have received combination treatment, he would have been treated with FVIII concentrate monotherapy both before and after the dental surgery. As his baseline FVIII:C was 0.04 IU/mL, his body weight 100 kg and the target FVIII peak level was 0.80-1.00 IU/mL, the FVIII loading dose would be (1.00-0.04)/0.02*100=4750 IU (rounded to entire vials). In the evening the patient would have received 2500 IU to maintain the FVIII:C between the target FVIII. Therefore, combination treatment hypothetically saved 2750 IU FVIII concentrate in this patient.

Ethics and dissemination

The study was approved by the Medical Ethics Committee of the Erasmus University Medical Centre Rotterdam, the Netherlands. The study will be conducted according to GCP guidelines and the Declaration of Helsinki. Also see Supplementary data 3-6 for our regulations for data storage, amendments and compensation for injury. Written informed consent will be obtained from all patients by a member of the research team (See Supplementary data 7 for the patient information, Dutch only). Results of the study will be communicated to the (inter)national

For peer review only - http://bmjopen.bmj.com/site/about/guidelines.xhtml

medical and scientific community trough publication in high-ranking peer reviewed international journals and at (inter)national medical scientific conferences. Results of the study will also be implemented in the Dutch Haemophilia Treatment Guidelines. Hopefully the international society of Haemophilia Treatment Centres will adapt the results in their guidelines as well.

Data monitoring committee and serious adverse events

This study does not carry any large safety risks as both FVIII concentrates and desmopressin are registered therapeutics for haemophilia treatment. In addition, to guarantee safety for all patients in this study, FVIII:C levels will be closely monitored to prevent any additional bleeding risks. Therefore a data safety monitoring board is not needed.

Serious adverse events (SAE) will be communicated to the sponsor within 24 hours. The sponsor will register the SAE within 15 days on *ToetsingOnline*, the Dutch registration system for SAEs.

Registration

The trial is registered in the Dutch Trial Registry, number NTR5383 (www.trialregister.nl) and in EudraCT: 2014-00535-14.

Discussion

This prospective trial will allow us to evaluate the safety and efficacy of combination treatment of desmopressin and FVIII concentrate in reaching target FVIII:C during bleeding episodes and in the perioperative setting. Dosing of FVIII concentrate will be determined by an integrated population PK model developed specifically for non-severe haemophilia A patients to be treated with combination treatment. Fifty non-severe haemophilia A patients will be included from eight haemophilia treatment centres in the Netherlands to reach our aim.

The DAVID study has some limitations. First, this study is not designed as a randomized controlled trial; it does not include a control group. Therefore, no direct comparison to standard treatment will be possible. However, we performed an extensive retrospective cohort study to determine the effectiveness of current clinical practice in which 37 patients undergoing 52 surgeries, were evaluated.[2] Moreover, the amount of FVIII concentrate that would have been administered to the patients included in the DAVID study as if desmopressin was not administered and as if no PK-guided dosing was applied, will be calculated.

The second limitation is the heterogeneity of the study population. All types of surgery, unless not compatible with desmopressin treatment, may be included in the study. To limit the

For peer review only - http://bmjopen.bmj.com/site/about/guidelines.xhtml

effects of this heterogeneity, only patients with an expected treatment duration of at least 48 hours will be included. Moreover, the data that were used to develop the integrated population PK model also included data from various types of surgery.

Conclusion

In the DAVID study, efficacy and safety of desmopressin and FVIII concentrate combination treatment in non-severe haemophilia A patients will be determined. Using this innovative approach treatment of non-severe haemophilia A patients both during bleeding episodes and in the perioperative setting may be improved.

Authors' contribution

MK, MCn, FL and RM designed the study and critically revised the manuscript. LS, SP and RH wrote the manuscript and refined the study design. MD, KF, EB, MCo, JE, BL-G, KM, LN and EM critically revised the manuscript and refined the study design.

We would like to specially thank MD for her contribution on behalf of the NVHP and the five NVHP members who helped to improve the patient information. In addition we would like to thank the pilot patient for his cooperation and for letting us use his case as an example.

Funding statement

This work was supported by ZonMw, (grant number 836031003), the Dutch organisation for Health Research and Development.

Competing interest statement

L.M. Schütte; received reimbursement from CSL-Behring for attending a symposium, not related to this study.

M.H. Cnossen; received unrestricted research/educational funding for various projects as well as travel fees from the following institutions and companies: ZonMW, Innovatiefonds, Pfizer, Baxalta/Shire, Bayer Schering Pharma, Novo Nordisk, Novartis, Roche and CSL Behring, all not related to this study.

R.M. van Hest, E.A.M. Beckers, M. Coppens, M. Driessens, L. Nieuwenhuizen, S.Polinder: nothing to disclose relevant to the DAVID study.

K. Fijnvandraat: is a member of the European Hemophilia Treatment and Standardization Board sponsored by Baxter, has received unrestricted research grants from CSL Behring and Bayer, and has given lectures at educational symposiums organized by Pfizer, Bayer and Baxter.

J. Eikenboom: received research funding from CSL Behring and honorarium for educational activity from Roche, not related to this study.

For peer review only - http://bmjopen.bmj.com/site/about/guidelines.xhtml

B.A.P. Laros-van Gorkom: received unrestricted educational grants from Baxter and CSL Behring, and speaker fees from Sanquin.

K. Meijer: research support from Bayer, Sanquin and Pfizer; speaker fees from Bayer, Sanquin, Boehringer Ingelheim, BMS and Aspen; consulting fees from Uniqure, not related to this study

F.W.G. Leebeek; received unrestricted research grants from CSL-Behring and Baxalta/Shire not related to this study. He is consulant for Shire, NovoNordisk and UniQure. Fees go to the university.

R.A.A. Mathôt: received personal fees from Merck Sharp & Dohme and Zeria and grants from Bayer, UCB Pharma and Hoffman La Roche with no involvement in this study.

M.J.H.A.Kruip; received unrestricted research grants from Pfizer, Innovatiefonds and Ferring with no involvement in this study.

References

1. Hazendonk HC, Lock J, Mathot RA, et al. Perioperative treatment of hemophilia A patients: blood group O patients are at risk of bleeding complications. J Thromb Haemost 2016;**14**(3):468-78.
2. Schütte LM, de Rooij N, Hazendonk HCAM, et al. Perioperative FVIII Concentrate Treatment in Mild Hemophilia a Patients Shows a High Rate of Overdosing - David/Opti-Clot Studies. Blood 2015;**126**(23):3510-10.
3. Ritchie B, Woodman RC, Poon MC. Deep venous thrombosis in hemophilia A. Am J Med 1992;**93**(6):699-700.
4. Escuriola Ettingshausen C, Martinez Saguer I, Kreuz W. Portal vein thrombosis in a patient with severe haemophilia A and F V G1691A mutation during continuous infusion of F VIII after intramural jejunal bleeding - Successful thrombolysis under heparin therapy. European Journal of Pediatrics, Supplement 1999;**158**(3):S180-S82.
5. Russell Z, Riconda D, Pollack L, et al. Thrombosis in a pregnant hemophilia A carrier after intrapartum recombinant factor VIII. Obstet Gynecol 2005;**105**(4):875-6.
6. Eckhardt CL, Menke LA, van Ommen CH, et al. Intensive peri-operative use of factor VIII and the Arg593-->Cys mutation are risk factors for inhibitor development in mild/moderate hemophilia A. J Thromb Haemost 2009;**7**(6):930-7.
7. Astermark J. FVIII inhibitors: pathogenesis and avoidance. Blood 2015;**125**(13):2045-51.
8. d'Oiron R, Pipe SW, Jacquemin M. Mild/moderate haemophilia A: new insights into molecular mechanisms and inhibitor development. Haemophilia 2008;**14 Suppl 3**:138-46.
9. Kaufmann JE, Vischer UM. Cellular mechanisms of the hemostatic effects of desmopressin (DDAVP). J Thromb Haemost 2003;**1**(4):682-9.
10. Mannucci. Desmopressin (DDAVP) in the treatment of bleeding disorders. World federation of hemophilia 2012.
11. Pan J, Dinh TT, Rajaraman A, et al. Patterns of expression of factor VIII and von Willebrand factor by endothelial cell subsets in vivo. Blood 2016.
12. Stoof SC, Sanders YV, Petrij F, et al. Response to desmopressin is strongly dependent on F8 gene mutation type in mild and moderate haemophilia A. Thromb Haemost 2013;**109**(3):440-9.
13. Nance D, Fletcher SN, Bolgiano DC, et al. Factor VIII mutation and desmopressin-responsiveness in 62 patients with mild haemophilia A. Haemophilia 2013;**19**(5):720-6.

For peer review only - http://bmjopen.bmj.com/site/about/guidelines.xhtml

14. Castaman G, Mancuso ME, Giacomelli SH, et al. Molecular and phenotypic determinants of the response to desmopressin in adult patients with mild hemophilia A. J Thromb Haemost 2009;**7**(11):1824-31.
15. Stoof SC, Cnossen MH, de Maat MP, et al. Side effects of desmopressin in patients with bleeding disorders. Haemophilia 2016;**22**(1):39-45.
16. Mannucci PM, Bettega D, Cattaneo M. Patterns of development of tachyphylaxis in patients with haemophilia and von Willebrand disease after repeated doses of desmopressin (DDAVP). Br J Haematol 1992;**82**(1):87-93.
17. Björkman S. Limited blood sampling for pharmacokinetic dose tailoring of FVIII in the prophylactic treatment of haemophilia A. Haemophilia 2010;**16**(4):597-605.
18. Hazendonk H, Fijnvandraat K, Lock J, et al. A population pharmacokinetic model for perioperative dosing of factor VIII in hemophilia A patients. Haematologica 2016.
19. Hazendonk HC, van Moort I, Fijnvandraat K, et al. The "OPTI-CLOT" trial. A randomised controlled trial on periOperative PharmacokineTIc-guided dosing of CLOTting factor concentrate in haemophilia A. Thromb Haemost 2015;**114**(3):639-44.
20. Schutte LM, van Hest RM, Stoof SCM, et al. Pharmacokinetic Modelling to Predict FVIII:C Response to Desmopressin and Its Reproducibility in Nonsevere Haemophilia A Patients. Thromb Haemost 2018.
21. Leebeek FWG, Mauser-Bunschoten EP. *Diagnostiek en behandeling van hemofilie en aanverwante hemostasestoornissen : richtlijn*. Alphen aan den Rijn: Van Zuiden Communications, 2009.
22. Kaatz S, Ahmad D, Spyropoulos AC, et al. Definition of clinically relevant non-major bleeding in studies of anticoagulants in atrial fibrillation and venous thromboembolic disease in non-surgical patients: communication from the SSC of the ISTH. J Thromb Haemost 2015;**13**(11):2119-26.
23. Schulman S, Kearon C, Subcommittee on Control of Anticoagulation of the S, et al. Definition of major bleeding in clinical investigations of antihemostatic medicinal products in non-surgical patients. J Thromb Haemost 2005;**3**(4):692-4.
24. Kwaliteitsinstituut voor de Gezondheidszorg, C. B. O. *Diagnostiek, preventie en behandeling van veneuze trombo-embolie en secundaire preventie van arteriële trombose*. Alphen aan den Rijn: Van Zuiden Communications, 2008.
25. Kwaliteitsinstituut voor de Gezondheidszorg CBO. *Diagnostiek, behandeling en zorg voor patienten met een beroerte : richtlijn*. Utrecht: Nederlandse Vereniging voor Neurologie; Utrecht: Kwaliteitsinstituut voor de Gezondheidszorg CBO, 2008.

For peer review only

For peer review only - http://bmjopen.bmj.com/site/about/guidelines.xhtml

Legends to tables and figures

Table 1: Model characteristics of the population PK model for FVIII concentrates

The covariate associations between VWF:Ag and clearance were modeled as a nonlinear function, while the association between most recent FVIII:C and baseline FVIII:C was modeled as a linear function. The numbers for the covariate associations (-0.285 and 0.891) describe both the shape and magnitude of the covariate effect: the more the number deviates from 0 the larger the effect of a covariate on the PK parameter. RSE = relative standard error; VWF:Ag = von Willebrand factor antigen; IOV = Inter-occasion variability

Table 2: Overview of blood sampling for FVIII:C measurements

Grey: obligatory measurements

† only in case of elective surgery, otherwise before first desmopressin administration

‡ pre = before desmopressin, post = after desmopressin, peak = after FVIII-concentrate, post-treat = after surgery; in case of bleeding; 2-6 hours after desmopressin, trough = before next dose of FVIII-concentrate

ǂ pre = before desmopressin, post = after desmopressin, peak = after loading dose FVIII-concentrate, post-treat = after surgery; in case of bleeding: 2-6 hours after desmopressin, pre-adjust = before dosage, steady state = FVIII-measurement at random time point

FVIII measurements are shown here until day 3 after start of combination treatment. Monitoring will continue if treatment is still necessary.

Table 3: Target ranges for FVIII:C in IU/ml in the perioperative setting[21]

Figure 1: Flowchart of study

Figure 2: FVIII:C course after desmopressin and FVIII concentrate combination treatment in a pilot patient with moderate haemophilia A (FVIII:C 0.04 IU/ml). T_0 = preoperative desmopressin infusion. Lines are the predicted FVIII:C. Predictions were based on a previous desmopressin test dose and FVIII concentrate administration, prior to study inclusion. Solid line is total FVIII:C and can be measured. Open circles are measured FVIII:C. Infusions of desmopressin and FVIII concentrate (FVIII con.) are depicted with arrows.

For peer review only - http://bmjopen.bmj.com/site/about/guidelines.xhtml

Table 1: Model characteristics of the population PK model for FVIII concentrates

Parameter	Population estimate	RSE (%)
Baseline FVIII:C (IU/ml)	0.211	10.9
Clearance (ml/h)	208	10
Volume of distribution (ml)	3400	4.9
Proportional error (%)	17.3	8.4
VWF:Ag on clearance	-0.285	5.4
Most recent FVIII:C on baseline FVIII:C	0.891	15.8
IOV of baseline FVIII:C (%)	54.7	11.0
IIV of clearance (%)	37.4	19.7
IIV of volume of distribution (%)	20.8	26.8

The covariate associations between VWF:Ag and clearance was modeled as a nonlinear function, while the association between most recent FVIII:C and baseline FVIII:C was modeled as a linear function. The numbers for the covariate associations (-0.285 and 0.891) describe both the shape and magnitude of the covariate effect: the more the number deviates from 0 the larger the effect of a covariate on the PK parameter. RSE = relative standard error; VWF:Ag = von Willebrand factor antigen; IOV = inter-occasion variability; IIV = Inter-individual variability

For peer review only

Table 2: Overview of blood sampling for FVIII:C measurements

	Within 4 weeks before surgery†	D0 = Day of first desmopressin infusion				D1				D2		D3	**4-8 weeks after FVIII treatment**
Time – bolus infusions‡	baseline	pre	post	peak	post-treat	pre	post	peak	pre	post	peak	trough	
Time – continuous infusion‡	baseline	pre	post	peak	post-treat	pre	post	pre-adjust	pre	post	pre-adjust	steady state	
Primary endpoint						X			X			X	
Sodium	X	X				X			X			X	
Neutralizing antibodies	X												X

Grey: obligatory measurements

† only in case of elective surgery, otherwise before first desmopressin administration

‡ pre = before desmopressin, post = after desmopressin, peak = after FVIII-concentrate, post-treat = after surgery; in case of bleeding; 2-6 hours after desmopressin, trough = before next dose of FVIII-concentrate

‡ pre = before desmopressin, post = after desmopressin, peak = after loading dose FVIII-concentrate, post-treat = after surgery; in case of bleeding: 2-6 hours after desmopressin, pre-adjust = before dosage, steady state = FVIII-measurement at random time point

FVIII measurements are shown here until day 3 after start of combination treatment. Monitoring will continue if treatment is still necessary.

Table 3: Target ranges for FVIII:C in IU/ml in the perioperative setting[21]

**Time**	**FVIII target level (IU/ml)**
**Day 0 (hour 0-24)**	0.8-1.0 (peak)
**Day 1-4 (hour 24-96)**	0.5-0.8 (trough)
**Day ≥ 5 (hour > 96)**	0.3-0.5 (trough)

For peer review only - http://bmjopen.bmj.com/site/about/guidelines.xhtml

For peer review only

For peer review only - http://bmjopen.bmj.com/site/about/guidelines.xhtml

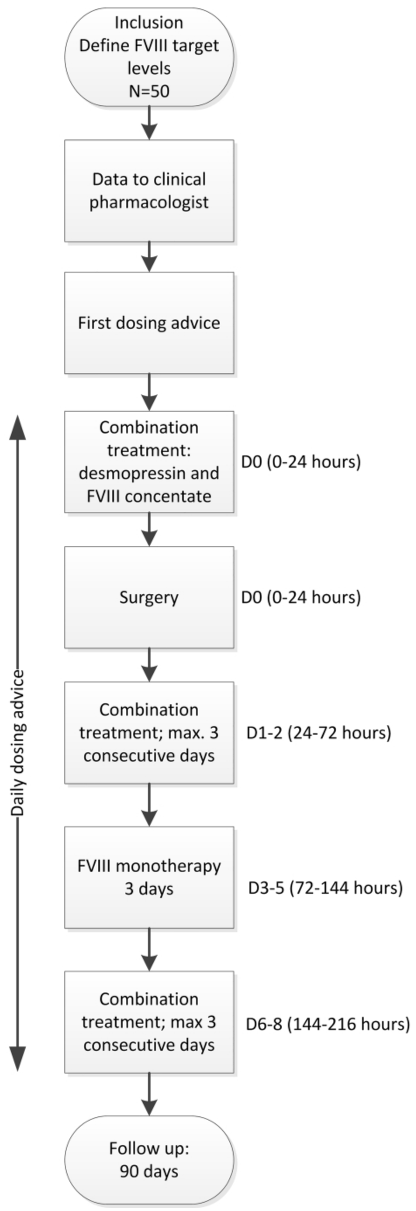

Figure 1: Flowchart of study

263x773mm (300 x 300 DPI)

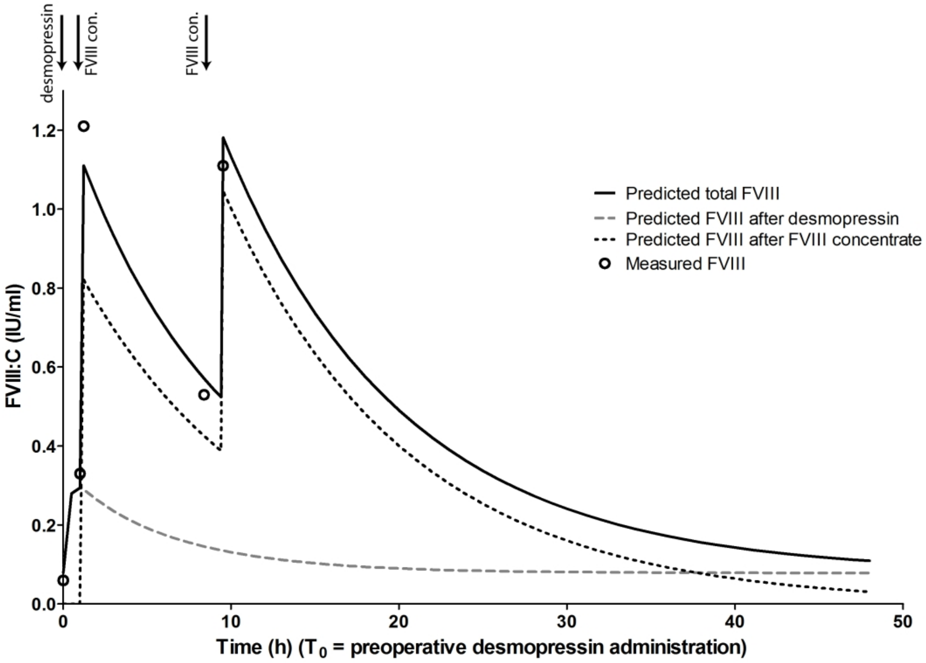

Figure 2: FVIII:C course after desmopressin and FVIII concentrate combination treatment in a pilot patient with moderate haemophilia A (FVIII:C 0.04 IU/ml). T0 = preoperative desmopressin infusion. Lines are the predicted FVIII:C. Predictions were based on a previous desmopressin test dose and FVIII concentrate administration, prior to study inclusion. Solid line is total FVIII:C and can be measured. Open circles are measured FVIII:C. Infusions of desmopressin and FVIII concentrate (FVIII con.) are depicted with arrows.

246x171mm (300 x 300 DPI)

Supplementary data

1) Steering committee
2) Participating centres
3) Handling and storage of data and documents
4) Amendments
5) Compensation for injury
6) Access to final trial dataset
7) Model for patient information and informed consent form (Dutch only)

For peer review only

1) Steering committee

Function	Contact data
Project Leader	Dr. M.J.H.A. Kruip, haematologist<br>T: 010-7033123, E: m.kruip@erasmusmc.nl<br>Department of Haematology, Erasmus University Medical Centre<br>Postbus 2040, 3000 CA Rotterdam
Sponsor	Prof. Dr. F.W.G. Leebeek, haematologist<br>T:010-7033123, E:f.leebeek@erasmusmc.nl<br>Department of Haematology, Erasmus University Medical Centre
Co-project leader	Dr. M.H. Cnossen, paediatric haematologist<br>T:010-7036691, E: m.cnossen@erasmusmc.nl<br>Department of Paediatric Haematology, Erasmus University Medical Centre
Co-project leader	Prof.dr. R.A.A. Mathôt, clinical pharmacologist<br>T:020-5663474, E:r.mathot@amc.uva.nl<br>Pharmacy, Amsterdam UMC
Project Advisor	Dr. M.H.E. Driessens<br>T:06-12192825020-6599021, E: m.driessens@nvhp.nl<br>Dutch Society of Haemophilia Patients
Promotor	Prof. dr. F.W.G. Leebeek, haematologist<br>T:010-7033123, E:f.leebeek@erasmus.nl<br>Department of Haematology, Erasmus University Medical Centre
Member of the steering committee	Dr. C.J. Fijn Van Draat<br>T: 020-5669111, E: c.j.fijnvandraat@amc.uva.nl<br>Department of Paediatric Haematology, Amsterdam UMC
Member of the steering committee	Dr. S. Polinder<br>T: 010-7043954, E:s.polinder@erasmusmc.nl<br>Department of Public Health, Erasmus University Medical Centre

For peer review only

2) List of participating centres

Medical Centre	Member and contact data
**Erasmus University Medical Centre**	Dr. M.J.H.A. Kruip, hematologist<br>T: 010-7033123, E: m.kruip@erasmusmc.nl<br>Department of Haematology<br>Postbus 2040, 3000 CA Rotterdam
**Amsterdam University Medical Centres**	Dr. M. Coppens<br>Department of haematology<br>m.coppens@amc.uva.nl
**Leiden University Medical Centre**	Prof. Dr. H.C.J. Eikenboom<br>Department of internal medicine, division of Thrombosis and Haemostasis<br>h.c.j.eikenboom@lumc.nl
**University Medical Centre Groningen**	Prof. dr. K. Meijer<br>Department of Haematology<br>k.meijer@umcg.nl
**Radboud University Medical Centre, Nijmegen**	Dr. B.A.P. Laros-van Gorkom<br>Department of Haematology<br>britta.laros-vangorkom@radboudumc.nl
**Maxima Medical Centre, Eindhoven**	Dr. L. Nieuwenhuizen<br>Department of Haematology<br>l.nieuwenhuizen@mmc.nl
**University Medical Centre Utrecht**	Dr. E.P. Mauser-Bunschoten<br>Van Creveldkliniek, Department of Haematology<br>e.mauserbunschoten@umcutrecht.nl
**Maastricht University Medical Centre**	Dr. E.A.M. Beckers<br>Department of Haematology<br>eam.beckers@mumc.nl

For peer review only

3) Handling and storage of data and documents

All data will be handled confidentially. To assure anonymity of all participating subjects, data will be coded. Only the different investigators at the participating sites and the treating physician will be able to match the study number to the patient's file by matching this number to the patient's hospital registration number. Handling of data will be done according to the Dutch Personal Data Protection Act. DNA and blood samples will be handled confidentially and stored in the hemostasis laboratory at the Erasmus MC. The material will be stored for a maximum of fifteen years.

For peer review only

4) Amendments

Amendments are changes made to the research after a favourable opinion by the accredited METC has been given. All substantial amendments will be notified to the METC and to the competent authority. Non-substantial amendments will not be notified to the accredited METC and the competent authority, but will be recorded and filed by the sponsor. All amendments will be communicated to all participating centres by the trial coordinator. Changes regarding inclusion and exclusion criteria or study design, will also be communicated to www.trialregister.nl (NTR 5383).

For peer review only

5) Compensation for injury

The sponsor/investigator has a liability insurance which is in accordance with article 7, subsection 9 of the WMO. The sponsor/investigator has a liability insurance which is in accordance with article 7 of the WMO. The sponsor (also) has an insurance which is in accordance with the legal requirements in the Netherlands (Article 7 WMO). This insurance provides cover for damage to research subjects through injury or death caused by the study. The insurance applies to the damage that becomes apparent during the study or within 4 years after the end of the study.

For peer review only

6) Access to final trial dataset

The sponsor of the DAVID study will have access to the final dataset and will be the owner of all study data. However, all participating centres have access to their own data and will have permission to use these data for non-commercial research within their own centre and to improve patient care. All publications regarding the DAVID study will be coordinated by the project leader. Other centres than the sponsor site may only publish data after permission from the sponsor and if the sponsor is not publishing the data within a fair amount of time after the study and data analysis has ended. All participating centres have contractual agreements with the sponsor regarding these matters.

In addition the sponsor has an agreement with the funder. The funder will be co-owner of the final dataset. All data has to be available for future research. The sponsor is obliged to publish all study data unless publication does not serve any public interest.

For peer review only

7) Model for patient information and informed consent form (Dutch only)

Informatiebrief DAVID-studie
Geachte patiënt,

Graag willen wij u met deze brief informeren over de DAVID-studie. DAVID staat voor *D**DAV**P treatment comb**I**ne**D** with factor VIII clotting factor concentrates in patients with mild hemophilia A*. Dit betekent: combinatiebehandeling van DDAVP en factor VIII-concentraten in patiënten met milde en matige hemofilie A.

U krijgt deze brief, omdat we willen vragen of u deel wilt nemen aan de DAVID-studie.
Lees deze brief rustig door en neem de tijd om na te denken over het al dan niet deelnemen aan de DAVID-studie. Bespreek het eventueel ook met uw partner, vrienden of familie. Lees ook de algemene brochure. Daarin staat veel algemene informatie over medisch-wetenschappelijk onderzoek. Heeft u na het lezen nog vragen? Dan kunt u altijd terecht bij uw behandelend arts of de onderzoeker. Ook is er een onafhankelijke arts, die veel weet van het onderzoek. Aan het einde van deze brief vindt u de contactgegevens.

Inleiding
U bent onder behandeling in verband met milde of matige hemofilie A, een zeldzame, erfelijke stoornis in de bloedstolling. Hierbij heeft u te weinig stollingsfactor VIII (factor VIII). Daardoor loopt u meer risico op bloedingen, zoals bij een kleine verwonding of tijdens een operatie. De behandeling hiervoor bestaat meestal uit het geven van factor VIII-concentraat, de ontbrekende stollingsfactor. Factor VIII-concentraat gedraagt zich in ieder lichaam anders. Dit betekent dat de ene patiënt meer factor VIII-concentraat nodig heeft dan de andere patiënt. Daar wordt nu niet genoeg rekening mee gehouden. Hierdoor krijgen sommige patiënten mogelijk te veel factor VIII-concentraat toegediend, terwijl andere patiënten juist te weinig krijgen.
Patiënten met milde en matige hemofilie A hebben nog een kleine hoeveelheid eigen factor VIII in het bloed en worden in sommige gevallen met DDAVP behandeld. Dit medicijn zorgt ervoor dat het eigen, opgeslagen factor VIII vrijkomt in het bloed. DDAVP is vaak niet voldoende om het factor VIII-gehalte in het bloed hoog genoeg te krijgen bij operaties. Een oplossing is om DDAVP te combineren met factor VIII-concentraat. Het factor VIII-gehalte stijgt dan in eerste instantie door de DDAVP. Vervolgens wordt er zo veel factor VIII-concentraat bijgegeven dat er voldoende factor VIII in het bloed zit. In deze studie willen wij bestuderen of de combinatiebehandeling goed werkt.

1. Wat is het doel van het onderzoek?
We willen onderzoeken of patiënten rondom operaties en bloedingen de juiste factor VIII-waarden in het bloed bereiken wanneer ze behandeld worden met een combinatiebehandeling van DDAVP en factor VIII-concentraat. Als dit het geval is, dan kan de combinatiebehandeling in de toekomst standaard gebruikt gaan worden. Dit zal er hopelijk toe leiden dat er minder factor VIII-concentraat nodig is. Wij willen dit bereiken zonder dat de behandeling slechter of minder veilig wordt.
Daarnaast willen we nagaan of de benodigde hoeveelheid factor VIII-concentraat per patiënt kan worden bepaald door van alle deelnemers aan de DAVID-studie verschillende gegevens te verzamelen.

2. Welke behandeling wordt onderzocht?
Tijdens dit onderzoek wordt de combinatiebehandeling van DDAVP en factor VIII-concentraat onderzocht. Beide medicijnen worden al gebruikt bij milde en matige hemofilie A-patiënten en u heeft ze mogelijk al eens gehad.

For peer review only

3. Hoe wordt het onderzoek uitgevoerd?

Iedereen die deelneemt aan dit onderzoek krijgt minimaal 2 dagen een combinatiebehandeling van DDAVP en factor VIII-concentraat rondom een operatie of een bloeding.

De combinatiebehandeling begint bij voorkeur vlak voor de ingreep. In het geval van een spoedingreep of bloeding kan het zijn dat u eerst maximaal 24 uur behandeld wordt met FVIII-concentraat voordat de combinatiebehandeling begint. De combinatiebehandeling duurt in eerste instantie maximaal drie aaneengesloten dagen. Dagelijks krijgt u eenmaal DDAVP toegediend via een infuus. Aansluitend krijgt u factor VIII-concentraat toegediend via een infuus, om de juiste factor VIII-waarden te bereiken. Afhankelijk van hoe lang u behandeling nodig heeft, beslist uw eigen arts of u van dag 6 t/m 8 opnieuw de combinatiebehandeling krijgt. Op alle overige behandeldagen ontvangt u alleen factor VIII-concentraat.

<u>DDAVP</u>

DDAVP krijgt u in de standaarddosering via een infuus. Om er zeker van te zijn dat dit middel bij u werkt, hebben we de resultaten van een DDAVP-test nodig. Dit kunnen resultaten zijn van een test die eerder is uitgevoerd. Indien er voor een geplande operatie geen testresultaten beschikbaar zijn of zijn ze niet geschikt, dan zal uw arts met u een nieuwe test afspreken voorafgaand aan de operatie. Tijdens de test zal u 1 dosis DDAVP toegediend krijgen via een infuus. Zowel voor als 2-3 keer na de toediening van DDAVP zal er bloed worden afgenomen om uw reactie op DDAVP te meten. Meer uitleg over de gang van zaken tijdens de test ontvangt u van uw eigen arts.

Hierboven heeft u kunnen lezen dat u maximaal 3 aaneengesloten dagen DDAVP krijgt. Dit doen we, omdat de voorraad van factor VIII na meerdere DDAVP-toedieningen minder wordt. Daarom is het belangrijk dat uw lichaam na 3 aaneengesloten dagen, 3 dagen ‘rust’ krijgt, waarin u geen DDAVP krijgt. Na 3 ‘rust’-dagen kunt u weer DDAVP ontvangen.

<u>Factor VIII-concentraten</u>

Gedurende het onderzoek krijgt u hetzelfde factor VIII-concentraat (bijvoorbeeld Novoeight of Kogenate) dat u anders ook zou krijgen. Op basis van de resultaten van de DDAVP-test en de gemeten factor VIII-waarden wordt er rondom de operatie of bloeding dagelijks een persoonlijke dosering bepaald en doorgegeven aan uw eigen arts. Uw eigen arts beslist hoe lang u factor VIII-concentraat toegediend krijgt en of het factor VIII-concentraat continu of in losse giften per dag gegeven wordt.

<u>Vragenlijst</u>

U krijgt voor de start van de combinatiebehandeling en 3 dagen erna een korte vragenlijst met 14 vragen over hoe u de zorg rondom de operatie of bloeding hebt ervaren. Deze vragenlijst richt zich op de behandeling van uw hemofilie A.

4. Wat wordt er van u verwacht?

Tijdens het onderzoek vragen wij u de voorschriften van uw arts te volgen. Dit houdt onder andere in dat u gedurende de behandeling met DDAVP niet meer vocht binnenkrijgt dan 1,5 liter per dag.

Verder is er een extra ziekenhuisbezoek nodig, indien u een DDAVP-test nodig hebt. Dit hoort u van uw eigen arts.

5. Wat is er meer of anders dan de reguliere behandeling?

De reguliere behandeling rondom operaties en bloedingen bij patiënten met hemofilie A bestaat uit het geven van factor VIII-concentraat. Tijdens het onderzoek krijgt u daarnaast DDAVP. U ontvangt de combinatiebehandeling maximaal twee perioden van 3 aaneengesloten dagen. Tussen deze twee

For peer review only

perioden, zitten 3 dagen zonder DDAVP. Indien nodig krijgt u voor een geplande operatie een DDAVP-test om te zien hoe u op deze medicatie reageert.
Daarnaast wordt onderzocht of u voldoende reageert op de combinatiebehandeling en of u de juiste dosering krijgt. Hiervoor nemen we tijdens de combinatiebehandeling, afhankelijk van de dag, 1-2 keer per dag vaker bloed af, 15 milliliter per keer. Zie hiervoor bijlage 2.
Verder onderzoeken we of veranderingen of verschillen in het DNA mogelijk samenhangen met de manier waarop u op de behandeling reageert. Hiervoor wordt eenmalig bloed afgenomen. Dit gebeurt alleen als u hiervoor toestemming geeft.
In bijlage 2 vindt u een schematisch overzicht van de onderzoeken. Hierop staat aangegeven welke behandelingen en onderzoeken extra zijn ten opzichte van de reguliere behandeling.

6. Wat zijn de mogelijke andere behandelingen?
Wanneer u besluit niet deel te nemen aan het onderzoek, wordt u behandeld met factor VIII-concentraat in de standaarddosering.

7. Welke bijwerkingen kunt u verwachten?
Bijwerkingen van DDAVP zijn van voorbijgaande aard en over het algemeen mild. De meest voorkomende bijwerkingen zijn een tijdelijke daling van de bloeddruk, een stijging van de hartslag, blozen, hoofdpijn en jeukende ogen. In zeldzame gevallen houdt het lichaam te veel vocht vast, waardoor het zoutgehalte in het bloed te laag kan worden.
De belangrijkste bijwerking van factor VIII-concentraten is een verhoogd risico op het ontwikkelen van remmers tegen factor VIII. Remmers zijn stoffen waardoor mogelijk de werkzaamheid van factor VIII-concentraten bij u in het vervolg verminderd kan zijn.

Niet alle bijwerkingen die bekend zijn, worden hier vermeld. Het is ook niet zo dat alle genoemde bijwerkingen bij elke patiënt zullen optreden. Wanneer u klachten krijgt, vragen wij u dit altijd aan uw eigen arts te melden.

8. Wat zijn de mogelijke voor- en nadelen van deelname?
Aan deelname zijn voor u geen directe voordelen verbonden. Met uw deelname kunt u wel de behandeling van milde en matige hemofilie A-patiënten helpen verbeteren.
Waarschijnlijk heeft u minder factor VIII-concentraat nodig dan u buiten de studie nodig zou hebben, omdat u ook DDAVP zal krijgen. Mogelijk kan dit het risico op het ontwikkelen van remmers tegen factor VIII verminderen. De ontwikkeling van remmers zorgt ervoor dat de behandeling met FVIII-concentraat minder effectief zal worden.
Het nadeel van deelname is dat er ten opzichte van de reguliere behandeling dagelijks 1 tot 2 extra bloedafnames plaatsvinden gedurende de ziekenhuisopname. Daarnaast moet u een DDAVP-test ondergaan als er geen geschikte eerdere resultaten beschikbaar zijn. Deze resultaten kunnen ook buiten de studie van nut zijn voor uw behandeling. Tot slot kunt u mogelijk bijwerkingen krijgen van de DDAVP.

9. Wat gebeurt er als u niet wenst deel te nemen aan het onderzoek?
Het is uw beslissing om wel of niet aan deze studie deel te nemen. U kunt besluiten om niet deel te nemen of uw toestemming tot deelname aan de studie op elk moment intrekken, zonder dat dit de zorg die u van artsen of verpleging in het ziekenhuis krijgt, beïnvloedt.

10. Wat gebeurt er als het onderzoek is afgelopen?
Het kan voorkomen dat u of uw behandelend arts besluit uw deelname aan het onderzoek te stoppen. U kunt dat zonder opgave van redenen en op ieder moment beslissen. U zal dan overgaan op de reguliere

For peer review only

behandeling die bestaat uit het toedienen van uitsluitend factor VIII-concentraat. Uw behandelend arts zal uw deelname aan het onderzoek alleen stoppen als dat noodzakelijk wordt geacht, bijvoorbeeld vanwege uw gezondheid.

11. Bent u verzekerd wanneer u aan het onderzoek meedoet?

Voor iedereen die aan dit onderzoek deelneemt, is een verzekering afgesloten. De verzekering dekt de schade als gevolg van het onderzoek. Dit geldt voor schade die tijdens het onderzoek optreedt. In bijlage 3 vindt u de verzekeringsgegevens.

12. Wordt u geïnformeerd als er tussentijds voor u relevante informatie over de studie bekend wordt?

Het onderzoek zal zo nauwkeurig mogelijk volgens plan verlopen. De situatie kan echter veranderen, bijvoorbeeld doordat er nieuwe informatie wordt verkregen. In dat geval wordt dat met u besproken. U beslist dan zelf of u met het onderzoek wilt stoppen of doorgaan. Als uw veiligheid of welbevinden in gevaar is, stoppen we direct met het onderzoek.

13. Wat gebeurt er met uw gegevens en lichaamsmateriaal?

In de algemene brochure is uitgelegd dat de onderzoeker gegevens over u verzamelt en deze vertrouwelijk behandelt. Dit betekent dat een aantal personen uw medische status en de gegevens van het onderzoek mogen inzien. Deze personen mogen de gegevens gebruiken voor dit onderzoek, maar zij mogen deze gegevens alleen bekend maken zonder daarbij uw naam of andere persoonlijke gegevens te vermelden. Uw identiteit blijft dus altijd geheim. De onderzoeker bewaart de gegevens met een code. Dit betekent dat op de studie- documenten in plaats van uw naam alleen een letter-cijfercode staat. Alleen de onderzoeker houdt een lijst bij waarop staat welke letter- cijfercode bij welke naam hoort.

Normaal gesproken heeft alleen uw behandelend arts en zijn/ haar team inzage in uw gegevens. Als u meedoet aan deze studie krijgen meer mensen inzage in uw medische gegevens en studiegegevens. De personen die inzage kunnen krijgen in uw gegevens zijn:

- de medewerkers van het onderzoeksteam,
- de leden van de toetsingscommissie die de studie heeft goedgekeurd,
- de bevoegde medewerkers van de Inspectie voor de Gezondheidszorg

Na de studie worden de gecodeerde gegevens gedurende 15 jaar bewaard. Dit is nodig om alles goed te kunnen controleren. Bovendien willen wij graag uw gegevens gebruiken voor andere onderzoeken die worden uitgevoerd naar milde en matige hemofilie A.
Deze onderzoeken hebben dus eenzelfde doel als het onderzoek waarvoor u nu wordt gevraagd. Het is dus niet zo dat uw gegevens ook zullen worden gebruikt voor onderzoek naar een geheel andere aandoening of een heel ander probleem. Vanzelfsprekend blijft de vertrouwelijkheid die we hierboven hebben beschreven altijd gelden. Vindt u het goed als wij uw gegevens gebruiken? Als u dat niet wilt, respecteren wij dat natuurlijk. U kunt uw keuze op het toestemmingsformulier aangeven.
Na de studie wordt uw lichaamsmateriaal (bloedmonsters en eventueel DNA) in gecodeerde vorm gedurende 15 jaar bewaard. Na een tijdelijke opslag in uw eigen ziekenhuis, zal het materiaal worden opgeslagen in het Erasmus Universitair Medisch Centrum Rotterdam. Wij willen dit materiaal graag gebruiken voor andere onderzoeken die worden uitgevoerd naar milde en matige hemofilie A. Deze onderzoeken hebben dus eenzelfde doel als het onderzoek waarvoor u nu wordt gevraagd. Het is dus niet zo dat uw materiaal ook zal worden gebruikt voor onderzoek naar een geheel andere aandoening of een heel ander probleem. Vanzelfsprekend blijft de vertrouwelijkheid die we hierboven hebben

For peer review only

beschreven altijd gelden. Vindt u het goed als wij uw materiaal bewaren en gebruiken? Als u dat niet wilt, respecteren wij dat natuurlijk. U kunt uw keuze op het toestemmingsformulier aangeven.

14. Wordt uw huisarts en/of behandelend specialist geïnformeerd bij deelname?
Wij laten uw huisarts schriftelijk weten dat u meedoet aan het onderzoek. Dit is voor uw eigen veiligheid. U moet hiervoor toestemming geven op het toestemmingsformulier. Als u geen toestemming geeft, kan u niet meedoen aan het onderzoek.

15. Krijgt u een vergoeding voor deelname?
Indien u voorafgaand aan de deelname aan de studie nog geen DDAVP-test gehad en u hiervoor een extra keer naar het hemofiliebehandelcentrum moet komen, dan krijgt u een vergoeding voor de reis- en eventuele parkeerkosten. Verder is er aan deelname aan de studie geen vergoeding verbonden.

16. Welke medisch-ethische toestingscommissie heeft dit onderzoek goedgekeurd?
Toetsingscommissie METC Erasmus MC heeft dit onderzoek goedgekeurd. Meer informatie over de goedkeuring vindt u in de algemene brochure.

17. Waar kunt u met vragen over het onderzoek terecht?
Met vragen over het onderzoek kunt u terecht bij uw hemofiliebehandelcentrum. In bijlage 1 vindt u de contactgegevens. Tevens vindt u daar de gegevens van een onafhankelijk arts aan wie u ook vragen kunt stellen over deelname aan het onderzoek.
Als u niet tevreden bent over het onderzoek of de behandeling, kunt u een klacht indienen bij de klachtencommissie. In bijlage 1 vindt u de benodigde contactgegevens.

Indien u na zorgvuldige overweging besluit deel te nemen aan dit wetenschappelijk onderzoek, dan vragen we u om samen met de onderzoeker het toestemmingsformulier te ondertekenen en te dateren.

Met vriendelijke groet,

Namens het onderzoeksteam,

Dr. Marieke J.H.A. Kruip en drs. Lisette M. Schütte

For peer review only

Bijlagen
1. Contactgegevens
2. Schema met onderzoeken
3. Informatie over de verzekering
4. Toestemmingsverklaring
5. Algemene brochure medisch-wetenschappelijk onderzoek met mensen

Bijlage 1 – contactgegevens
U kunt meer informatie over het onderzoek krijgen bij de onderzoeker of bij één van uw behandelaren:

Hematologen:
<Namen >

Verpleegkundig specialist hemofilie:
<Namen >

Hemofilieverpleegkundigen:
<Namen >

Contactgegevens:
Hemofiliebehandelcentrum <naam zh>:
<adres HBC>
e-mail: <emailadres HBC >
david@erasmusmc.nl

Secretariaat hematologie: <tel>

Onderzoekers:
Dr. Marieke J.H.A. Kruip
Drs. L.M. Schütte

Onafhankelijk deskundige
Als u twijfelt over deelname aan dit onderzoek, kunt u ook een onafhankelijk arts raadplegen, die zelf niet bij het onderzoek is betrokken, maar wel deskundig is op dit gebied:
Dr. P.A.W. te Boekhorst, internist-hematoloog, tel: 010-7033123
Ook als u vragen voor of tijdens het onderzoek hebt, die u liever niet aan de onderzoekers stelt, kunt u contact opnemen met de onafhankelijke arts.

Klachten
Een klacht kunt u indienen bij
<Onafhankelijke klachtencommissie of andere procedure>

Bijlage 2 – Schema met onderzoeken; zodra de behandeling is afgerond, zijn bloedafnames niet meer nodig
In het geval van een geplande ingreep

	Screening	Opnameperiode					Vervolg periode
Onderzoek	Voor de operatie	Dag van de operatie	Dag 1 na de operatie	Dag 2 na de operatie indien nodig	Dag 3-5 na de operatie indien nodig	Dag 6-8 na de operatie indien nodig	8-10 weken na operatie
DNA-afname*	V						
DDAVP-test*	V						
Bloedonderzo	V	X-V-X-X	X-V-V	X-V-V	X	X-V-V	

For peer review only

ek**/***							
DDAVP-toediening**		V	V	V		V	
factor VIII-concentraat volgens doseeradvies**		X	X	X	X	X	
Vragenlijst	V				V		
Bloedonderzoek remmers							X

*alleen indien nodig; zie eerdere informatie in de brief
**alleen indien behandeling nog noodzakelijk is; dit hoort u van uw eigen arts
***hier zijn alle bloedafnamen genoemd, ook de standaard afnames buiten de studie.
X: Deze onderzoeken en behandelingen zijn standaard.
V: Deze onderzoeken en behandelingen zijn extra voor de studie.

In het geval van een spoedingreep of een bloeding

	Opnameperiode					Vervolg periode
Onderzoek	Dag van start DDAVP	Dag 1 na start DDAVP	Dag 2 na start DDAVP indien nodig	Dag 3-5 na start DDAVP indien nodig	Dag 6-8 na start DDAVP indien nodig	8-10 weken na operatie /bloeding
DNA-afname*	V					
Bloedonderzoek**/***	X-V-X-X	X-V-V	X-V-V	X	X-V-V	
DDAVP-toediening**	X	X	X		X	
factor VIII-concentraten volgens doseeradvies**	X	X	X	X	X	
Vragenlijst	V			V		
Bloedonderzoek remmers	V					X

*alleen indien nodig; zie eerdere informatie in de brief
** alleen indien behandeling nog noodzakelijk is; dit hoort diegene die u vertegenwoordigt, van zijn eigen arts
*** hier zijn alle bloedonderzoeken genoemd, ook de bloedonderzoeken die behoren tot de standaardzorg.
X: Deze onderzoeken en behandelingen zijn standaard.
V: Deze onderzoeken en behandelingen zijn extra voor de studie

For peer review only

Bijlage 3 – Informatie over de verzekering

Voor iedereen die meedoet aan dit onderzoek, heeft het Erasmus MC een verzekering afgesloten. De verzekering dekt schade door deelname aan het onderzoek. Dit geldt voor schade tijdens het onderzoek of binnen vier jaar na het einde ervan. Schade moet u binnen die vier jaar aan de verzekeraar hebben gemeld.

De verzekering dekt niet alle schade. Onderaan deze tekst staat in het kort welke schade niet wordt gedekt.

Deze bepalingen staan in het Besluit verplichte verzekering bij medisch-wetenschappelijk onderzoek met mensen. Dit besluit staat op www.ccmo.nl, de website van de Centrale Commissie Mensgebonden Onderzoek (zie 'Bibliotheek' en dan 'Wet- en regelgeving').

Bij schade kunt u direct contact leggen met:

Van Ameyde
Ter attentie van mw. K. Roberts
Postbus 3038
2280 GA Rijswijk
T +31 (0) 70 413 7300
marketform@vanameyde.com

De verzekering biedt een dekking van € 650.000 per proefpersoon en € 5.000.000 voor het hele onderzoek (en € 7.500.000 per jaar voor alle onderzoeken van dezelfde opdrachtgever).

De verzekering dekt de volgende schade niet:

- schade door een risico waarover u in de schriftelijke informatie bent ingelicht. Dit geldt niet als het risico zich ernstiger voordoet dan was voorzien of als het risico heel onwaarschijnlijk was;
- schade aan uw gezondheid die ook zou zijn ontstaan als u niet aan het onderzoek had meegedaan;
- schade door het niet (volledig) opvolgen van aanwijzingen of instructies;
- schade aan uw nakomelingen, als gevolg van een negatief effect van het onderzoek op u of uw nakomelingen;
- schade door een bestaande behandelmethode bij onderzoek naar bestaande behandelmethoden.

For peer review only

Betreft: DAVID-studie

Toestemmingsformulier voor volwassen deelnemers

Naam: .... Geboortedatum: __ / __ / __

Ik bevestig dat ik de informatiebrief voor deelnemers heb gelezen. Ik begrijp de informatie. Ik heb de gelegenheid gehad om aanvullende vragen te stellen. Deze vragen zijn naar tevredenheid beantwoord. Ik heb voldoende tijd gehad om over deelname na te denken.

Ik weet dat mijn deelname geheel vrijwillig is en dat ik mijn toestemming op ieder moment kan intrekken, zonder dat ik daarvoor een reden hoef te geven.

Ik geef toestemming voor deelname aan bovengenoemd onderzoek.

Ik geef toestemming om mijn huisarts te vertellen dat ik meedoe aan dit onderzoek.

Ik geef toestemming aan bevoegde personen van het onderzoeksteam, medewerkers van de Inspectie voor de Gezondheidszorg en leden van de medisch-ethische toetsingscommissie voor inzage in mijn medische gegevens en onderzoeksgegevens.

Ik geef toestemming om de gegevens te verwerken voor de doeleinden zoals beschreven in de informatiebrief.

Ik geef toestemming voor het bewaren van de onderzoeksgegevens na afloop van het onderzoek voor een periode van 15 jaar.

Ik geef toestemming om mijn huisarts in te lichten over deelname aan dit onderzoek.

U wordt gevraagd in onderstaande zinnen door te halen wat niet van toepassing is.

Ik geef **WEL/GEEN** toestemming om de onderzoeksgegevens te gebruiken voor toekomstig onderzoek.

Ik geef **WEL/GEEN** toestemming om lichaamsmateriaal te gebruiken voor toekomstig onderzoek en te bewaren voor een periode van maximaal 15 jaar.

Ik geef **WEL/GEEN** toestemming om DNA-materiaal te gebruiken voor toekomstig onderzoek en te bewaren voor een periode van maximaal 15 jaar.

Ik wil meedoen aan dit onderzoek.

Naam deelnemer :

Handtekening : Datum : __ / __ / __

Hierbij verklaar ik, de onderzoeker, dat ik de deelnemer in de gelegenheid heb gesteld om aanvullende vragen te stellen, welke ik alle naar waarheid heb beantwoord.
Alle gegevens die mij inzake het onderzoek ter inzage zullen komen, zal ik geheimhouden en met respect behandelen.

Naam onderzoeker (of diens vertegenwoordiger) :

Handtekening : Datum : __ / __ / __

For peer review only

STANDARD PROTOCOL ITEMS: RECOMMENDATIONS FOR INTERVENTIONAL TRIALS

SPIRIT 2013 Checklist: Recommended items to address in a clinical trial protocol and related documents*

Section/item	Item No	Description	Addressed on page number
**Administrative information**			
Title	1	Descriptive title identifying the study design, population, interventions, and, if applicable, trial acronym	1____________
Trial registration	2a	Trial identifier and registry name. If not yet registered, name of intended registry	2_& 13______
	2b	All items from the World Health Organization Trial Registration Data Set	Manuscript+ supplementary data
Protocol version	3	Date and version identifier	NA_________
Funding	4	Sources and types of financial, material, and other support	14____________
Roles and responsibilities	5a	Names, affiliations, and roles of protocol contributors	_1 & 14
	5b	Name and contact information for the trial sponsor	1
	5c	Role of study sponsor and funders, if any, in study design; collection, management, analysis, and interpretation of data; writing of the report; and the decision to submit the report for publication, including whether they will have ultimate authority over any of these activities	14
	5d	Composition, roles, and responsibilities of the coordinating centre, steering committee, endpoint adjudication committee, data management team, and other individuals or groups overseeing the trial, if applicable (see Item 21a for data monitoring committee)	supplementary data 1+2
**Introduction**			

For peer review only - http://bmjopen.bmj.com/site/about/guidelines.xhtml

Background and rationale	6a	Description of research question and justification for undertaking the trial, including summary of relevant studies (published and unpublished) examining benefits and harms for each intervention	4 & 5
	6b	Explanation for choice of comparators	NA
Objectives	7	Specific objectives or hypotheses	5
Trial design	8	Description of trial design including type of trial (eg, parallel group, crossover, factorial, single group), allocation ratio, and framework (eg, superiority, equivalence, noninferiority, exploratory)	6
**Methods: Participants, interventions, and outcomes**			
Study setting	9	Description of study settings (eg, community clinic, academic hospital) and list of countries where data will be collected. Reference to where list of study sites can be obtained	6
Eligibility criteria	10	Inclusion and exclusion criteria for participants. If applicable, eligibility criteria for study centres and individuals who will perform the interventions (eg, surgeons, psychotherapists)	6
Interventions	11a	Interventions for each group with sufficient detail to allow replication, including how and when they will be administered	7
	11b	Criteria for discontinuing or modifying allocated interventions for a given trial participant (eg, drug dose change in response to harms, participant request, or improving/worsening disease)	NA
	11c	Strategies to improve adherence to intervention protocols, and any procedures for monitoring adherence (eg, drug tablet return, laboratory tests)	7
	11d	Relevant concomitant care and interventions that are permitted or prohibited during the trial	7
Outcomes	12	Primary, secondary, and other outcomes, including the specific measurement variable (eg, systolic blood pressure), analysis metric (eg, change from baseline, final value, time to event), method of aggregation (eg, median, proportion), and time point for each outcome. Explanation of the clinical relevance of chosen efficacy and harm outcomes is strongly recommended	9+10
Participant timeline	13	Time schedule of enrolment, interventions (including any run-ins and washouts), assessments, and visits for participants. A schematic diagram is highly recommended (see Figure)	Table 2

Sample size	14	Estimated number of participants needed to achieve study objectives and how it was determined, including clinical and statistical assumptions supporting any sample size calculations	9
Recruitment	15	Strategies for achieving adequate participant enrolment to reach target sample size	9

Methods: Assignment of interventions (for controlled trials)

Allocation:

Sequence generation	16a	Method of generating the allocation sequence (eg, computer-generated random numbers), and list of any factors for stratification. To reduce predictability of a random sequence, details of any planned restriction (eg, blocking) should be provided in a separate document that is unavailable to those who enrol participants or assign interventions	NA
Allocation concealment mechanism	16b	Mechanism of implementing the allocation sequence (eg, central telephone; sequentially numbered, opaque, sealed envelopes), describing any steps to conceal the sequence until interventions are assigned	NA
Implementation	16c	Who will generate the allocation sequence, who will enrol participants, and who will assign participants to interventions	NA
Blinding (masking)	17a	Who will be blinded after assignment to interventions (eg, trial participants, care providers, outcome assessors, data analysts), and how	NA
	17b	If blinded, circumstances under which unblinding is permissible, and procedure for revealing a participant's allocated intervention during the trial	NA

Methods: Data collection, management, and analysis

Data collection methods	18a	Plans for assessment and collection of outcome, baseline, and other trial data, including any related processes to promote data quality (eg, duplicate measurements, training of assessors) and a description of study instruments (eg, questionnaires, laboratory tests) along with their reliability and validity, if known. Reference to where data collection forms can be found, if not in the protocol	8-10
	18b	Plans to promote participant retention and complete follow-up, including list of any outcome data to be collected for participants who discontinue or deviate from intervention protocols	10-11

For peer review only - http://bmjopen.bmj.com/site/about/guidelines.xhtml

Data management	19	Plans for data entry, coding, security, and storage, including any related processes to promote data quality (eg, double data entry; range checks for data values). Reference to where details of data management procedures can be found, if not in the protocol	Supplementary data 3
Statistical methods	20a	Statistical methods for analysing primary and secondary outcomes. Reference to where other details of the statistical analysis plan can be found, if not in the protocol	10
	20b	Methods for any additional analyses (eg, subgroup and adjusted analyses)	10+11
	20c	Definition of analysis population relating to protocol non-adherence (eg, as randomised analysis), and any statistical methods to handle missing data (eg, multiple imputation)	NA
**Methods: Monitoring**			
Data monitoring	21a	Composition of data monitoring committee (DMC); summary of its role and reporting structure; statement of whether it is independent from the sponsor and competing interests; and reference to where further details about its charter can be found, if not in the protocol. Alternatively, an explanation of why a DMC is not needed	13
	21b	Description of any interim analyses and stopping guidelines, including who will have access to these interim results and make the final decision to terminate the trial	NA
Harms	22	Plans for collecting, assessing, reporting, and managing solicited and spontaneously reported adverse events and other unintended effects of trial interventions or trial conduct	13
Auditing	23	Frequency and procedures for auditing trial conduct, if any, and whether the process will be independent from investigators and the sponsor	NA
**Ethics and dissemination**			
Research ethics approval	24	Plans for seeking research ethics committee/institutional review board (REC/IRB) approval	12
Protocol amendments	25	Plans for communicating important protocol modifications (eg, changes to eligibility criteria, outcomes, analyses) to relevant parties (eg, investigators, REC/IRBs, trial participants, trial registries, journals, regulators)	Supplementary data 4

For peer review only - http://bmjopen.bmj.com/site/about/guidelines.xhtml

Consent or assent	26a	Who will obtain informed consent or assent from potential trial participants or authorised surrogates, and how (see Item 32)	12
	26b	Additional consent provisions for collection and use of participant data and biological specimens in ancillary studies, if applicable	Supplementary data 3+7 (Dutch only)
Confidentiality	27	How personal information about potential and enrolled participants will be collected, shared, and maintained in order to protect confidentiality before, during, and after the trial	Supplementary data 3
Declaration of interests	28	Financial and other competing interests for principal investigators for the overall trial and each study site	14+15
Access to data	29	Statement of who will have access to the final trial dataset, and disclosure of contractual agreements that limit such access for investigators	Supplementary data 6
Ancillary and post-trial care	30	Provisions, if any, for ancillary and post-trial care, and for compensation to those who suffer harm from trial participation	Supplementary data 5
Dissemination policy	31a	Plans for investigators and sponsor to communicate trial results to participants, healthcare professionals, the public, and other relevant groups (eg, via publication, reporting in results databases, or other data sharing arrangements), including any publication restrictions	12
	31b	Authorship eligibility guidelines and any intended use of professional writers	NA
	31c	Plans, if any, for granting public access to the full protocol, participant-level dataset, and statistical code	NA
**Appendices**			
Informed consent materials	32	Model consent form and other related documentation given to participants and authorised surrogates	Supplementary data 7 (Dutch only)
Biological specimens	33	Plans for collection, laboratory evaluation, and storage of biological specimens for genetic or molecular analysis in the current trial and for future use in ancillary studies, if applicable	Supplementary data 3

*It is strongly recommended that this checklist be read in conjunction with the SPIRIT 2013 Explanation & Elaboration for important clarification on the items. Amendments to the protocol should be tracked and dated. The SPIRIT checklist is copyrighted by the SPIRIT Group under the Creative Commons "Attribution-NonCommercial-NoDerivs 3.0 Unported" license.

For peer review only

For peer review only - http://bmjopen.bmj.com/site/about/guidelines.xhtml
